# Solventless Catalytic C–H and C–X Functionalization without Ball Milling

**DOI:** 10.1021/acs.joc.5c00109

**Published:** 2025-06-19

**Authors:** Carolina Méndez-Gálvez, Fredrik Barnå, Boris Olsthoorn, David Estébanez Rosich, Paul J. Gates, Lukasz T. Pilarski

**Affiliations:** a Department of Chemistry − BMC, Uppsala University, P.O. Box 576, Uppsala 75123, Sweden; b School of Chemistry, 1980 University of Bristol, Cantock’s Close, Clifton, Bristol BS8 1TS, United Kingdom

## Abstract

A low-cost, operationally simple approach to eight different solventless Rh-, Ru-, Ir-, and Pd-catalyzed C–H and C–X functionalizations, as well as the synthesis of difficult-to-make rhodacyclic complexes, is presented. The method uses open-air grinding and heating, is reproducible, gives competitive yields compared with automated ball milling protocols, and can be extended to the late-stage modification of various bioactive compounds.

## Graphical abstract

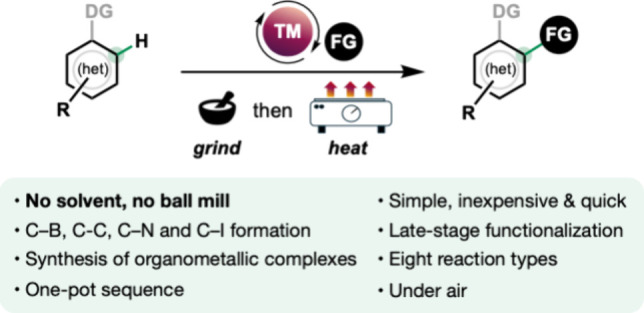

## Introduction

Solvent waste poses a major sustainability challenge for chemical synthesis. Annually, > 28 million tons of solvent are consumed worldwide^1^ and solvent use accounts for up to 85% of waste mass generated by pharmaceutical production alone.^2^ Moreover, the adverse health consequences of exposure to some solvents drive increasingly strict regulation of their use.^3^

Mechanochemistry obviates the use of bulk solvents as reaction media by using physical forces to mix and activate reagents.^4^ It has been at the forefront of efforts to align synthesis more closely to green chemistry principles^5^ and has wrought various other advantages,^6^ including improved safety^7^ and access to novel mechanistic pathways.^4c,8^ Numerous mechanochemical coupling reactions traditionally considered the province of solution-phase catalysis^4d,9^ have been developed, including various C–H functionalizations.^10^ C–H functionalization has in its own right matured into a powerful technology, not least for streamlining and improving the sustainability of organic synthesis.^11^

Automated ball milling has become the default technique for conducting solventless reactions at the laboratory scale.^12^ It boasts the systemization of conditions and, among myriad other benefits, new possibilities for interrogating solventless reaction mechanisms.^13^ While ball milling can supersede solvothermal conditions by using physical force to achieve reagent mixing and activation, there is a conspicuous absence of comparisons to other approaches that might achieve this, raising the question of whether the automated ball milling apparatus is strictly necessary in many cases.^14^ Ball milling studies typically specify various reaction parameters, including milling frequency, the materials of the jars and balls, liquid assisted grinding (LAG),^15^ intermissions and exclusion of air. Also, parameter reporting is not standardized; potentially important details, such as the internal temperatures of reaction vessels or the weight and size of ball bearings used, are often omitted.

Here, we show that a variety of transition metal-catalyzed C–H and C–X bond formations proceeds effectively using a rapid, low-cost ‘grind-and-heat’ (G&H) approach involving sequential pestle-and-mortar grinding and solventless heating. We compare the performance of G&H with that of ball milling and extend the scope, including to late-stage functionalization^16^ (LSF) of biologically active substrates, solventless versions of which are still remarkably rare.^17^ We also demonstrate one pot G&H reactivity, in which transformations take place sequentially without solvent or intermediate purification.

Previously, we described catalytic C–H methylation under ball milling conditions (Figure 1).^17a^ Subsequently, we showed comparable or equivalent yields could be obtained using a G&H approach where reagent mixing and activation are delivered simply through manual grinding of the reaction components using a pestle and mortar before heating in a glass vial for 0.5–2 h, without stirring or an inert atmosphere.^17b^ Importantly, this proved highly reproducible, both between reaction runs and different operators. Thus, G&H could provide *E*-factors^18^ identical to the ball milling protocol (5–25 times lower than the solution phase equivalent,^19^ Figure 1), without specialized equipment.

**Figure 1:**
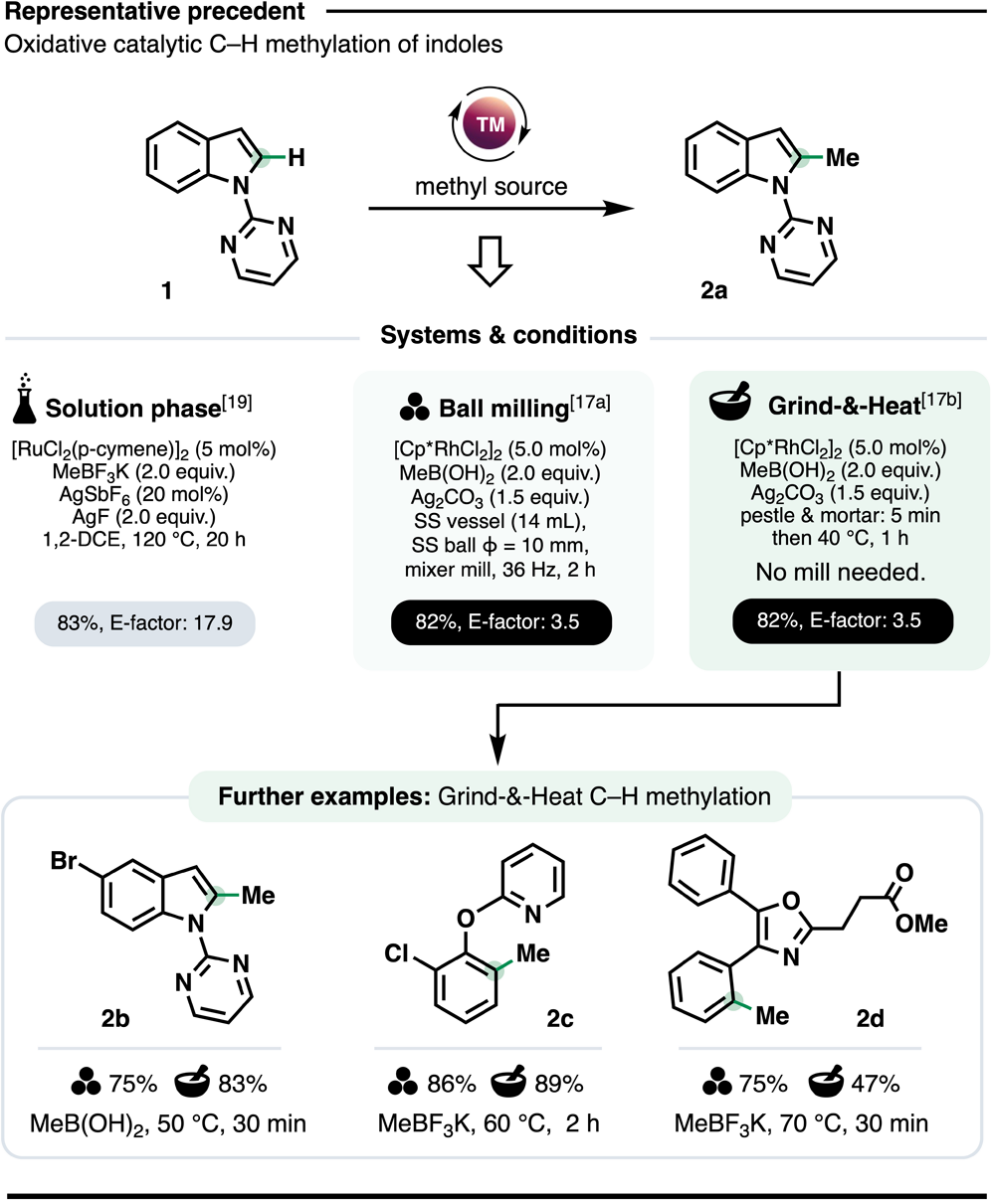
Top: Solution phase, ball milling and G&H conditions for oxidative indole C–H methylation. Bottom: Ball milling and G&H compared for three example compounds.

## Results & Discussion

### Solventless Rh-Based C–H Functionalizations

We began examining the generalizability of G&H with Rh-catalyzed C–H iodination^20,21^ and alkenylation (Scheme 1).^22^ Previously, **3a** could be converted to **4a** in either 78% or 84% yield using a planetary ball mill at 800 rpm or a mixer mill at 30 Hz, respectively.^20,21,23^ For G&H, we maintained the relative loadings of all reagents but replaced ball milling with pestle and mortar grinding (5 min) and heating the resulting mixture at 70 °C^24^ for 2 h under air without stirring. This gave **4a** and **4a’** (Scheme 1) in 90% spectroscopic yield (**4a**/**4a’** = 53:47). Heating at 90 °C instead gave a combined yield of 91% (**4a**/**4a’** = 60:40), which corresponds to a comparable yield of **4a** (76%) in the ball milling study.^20^ G&H could be extended to a derivative of the NSAID oxaprozin (product **4b**, 68% yield), and to access an iodinated analog of the herbicide diflufenican (**4c**). Compounds **4b** and **4c** are the first examples of late-stage C–H halogenation under solventless conditions.^25^

**Scheme 1:**
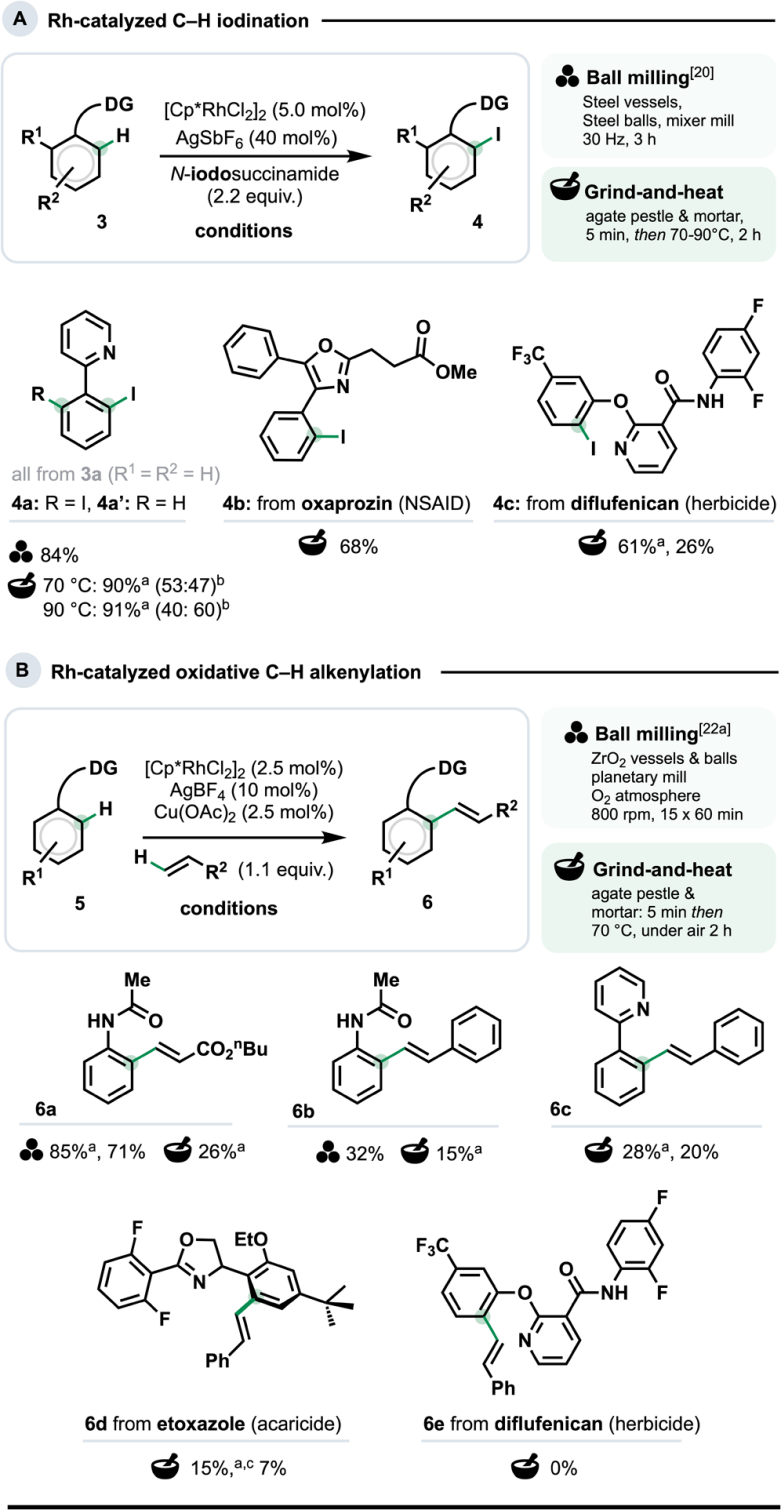
(A, B) Rh-Catalyzed C–H Functionalization under Ball Milling and G&H Conditions^a^

a G&H reactions at 0.3 mmol scale. ^a^Yield determined by ^1^H NMR spectroscopy using 1,3,5-trimethoxybenzene as an internal standard. ^b^ **4a**/**4a′** regioisomeric ratio. ^c^Identical spectroscopic yield obtained for reactions at 70 and 90 °C.

Scheme 1b shows G&H extended to Rh-catalyzed oxidative C–H alkenylation. This was originally reported with 1,2-dichloroethane as the optimal solvent, the use of which has since become legally restricted.^3b^ The original reaction proceeded at 120 °C over 16 h with stoichiometric Cu­(OAc)_2_ as the terminal oxidant under Ar.^26^ A later study used a planetary ball mill at 800 rpm with ZrO_2_ balls and vessels over 15 h.^22a^ For this, catalytic Cu­(OAc)_2_ sufficed with O_2_ as the terminal oxidant. Under G&H (grinding 5 min, heating at 70 °C under air) anilides **6a** and **6b** formed in 26% and 15% spectroscopic yields, respectively. Using N­(sp^2^)-based directing groups also proved viable (**6c** and **6d**). Despite the comparatively low yields for this specific set of conditions, the amounts of **6d** obtained from the acaricide etoxazole would suffice for biological testing. In contrast to **4c,** in the C–H iodination, no conversion of diflufenican was observed, presumably due to the difficultly of the alkene insertion to make an 8-membered, sterically congested rhodacycle.^27^

The mechanochemical synthesis of coordination and organometallic complexes has attracted significant attention.^6a,20,28^ This includes metallacycles,^13a,20,29^ which are intermediates in many catalytic C–H functionalizations,^30^ such as those in Scheme 1. Previously, we found rhodacycles generally form in much higher yields under ball milling than in solution,^17a^ while Hernández and co-workers have shed valuable light on their role in mechanochemical *ortho*-directed C–H activation.^13a,29b^

We wanted to compare G&H to solution phase and ball milling conditions for preparing rhodacycles **7** (Scheme 2), which are congeners of on-cycle intermediates for the reactions in Scheme 1. Literature solution-phase reactions to form rhodacycles, e.g., **7a**, can give poor to vanishingly small yields, especially for 6-membered variants (e.g., 24% for **7b** and 2% for **7c**).^17a^ Moreover, they usually rely on CH_2_Cl_2_, a Category 2 carcinogen.^31^ By contrast, our G&H protocol compared very favorably to the solution-phase reactions for 6-membered rhodacycles: **7b** and **7c** formed in 80% and 59% yield, respectively, underscoring that solventless approaches offer significant advantages, even without specialized equipment.

**Scheme 2:**
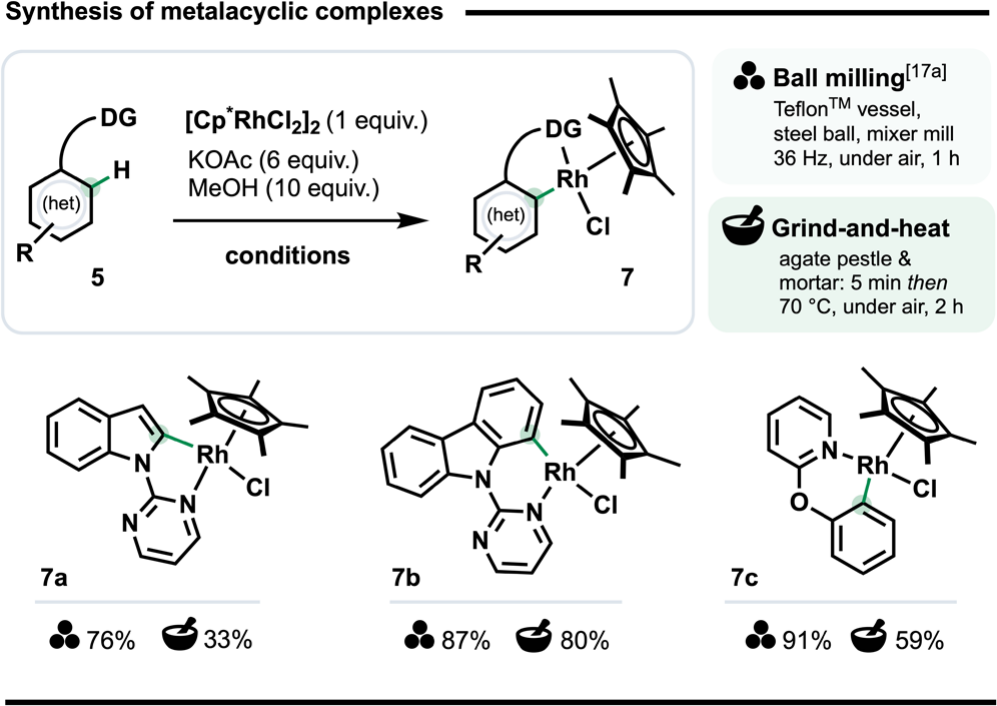
Synthesis of Metalacyclic Complexes Using G&H (0.2 mmol Scale)

### Solventless Ru-Catalyzed Alkyne Hydroarylation

Although Ru-catalyzed C–H functionalization has advanced remarkably in recent years,^32^ mechanochemical versions remain notably sparse.^33^ Among these is Bolm and co-workers’ C–H *ortho*-alkenylation of anilides and pyrazoles,^33b^ the scope of which was more recently expanded by Kumar’s group to encompass N­(sp^2^)-containing heteroarenes.^34^

Substrates **5** (Scheme 3a) incorporate representative O- and N-based directing groups tested using G&H. These gave the mono- (**8a**) and dialkylated (**8b**) products through appropriate alkyne loadings (1.2 and 2.0 equiv., respectively), mirroring the control possible in the ball milling versions. For **8a**, ball milling proved somewhat higher yielding than G&H (83% vs 57% yield), whereas for **8b** the differences between G&H (75%) and the ball milling versions of the reaction (79–80%) were close to negligible. Unlike ball milling, G&H did not need an inert atmosphere. The G&H hydroarylation could also be extended to etoxazole, a commercialized acaricide (derivative **8c**).

**Scheme 3:**
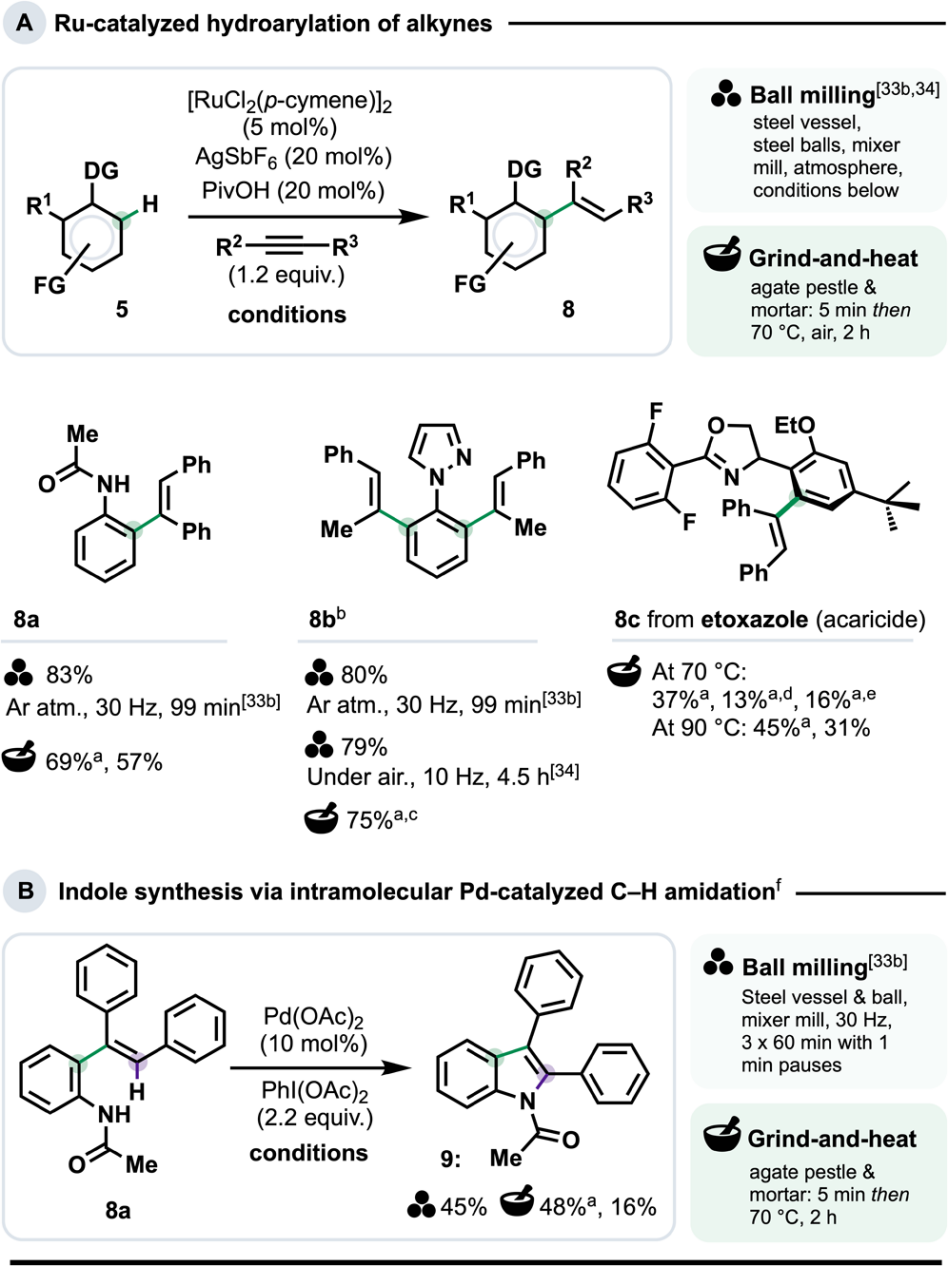
(A) Ru-Catalyzed Hydroarylation of Alkynes *via* C–H Activation; (B) Oxidative C–H Amidation^a^

a G&H at 0.3 mmol scale. ^a^Yield determined by ^1^H NMR spectroscopy using 1,3,5-trimethoxybenzene as internal standard. ^b^2.0 equiv of alkyne. ^c^Heating at 60 °C. ^d^5 min magnetic stirring (400 rpm) instead of grinding. ^e^Heated without grinding step. ^f^0.16 mmol scale.

Generation of ruthenacyclic derivatives of **5** *via* was unsuccessful under either ball milling or G&H conditions; both gave intractable mixtures, with ^1^H NMR spectroscopy indicating free *p*-cymene. Neutral η^6^-bound *p*-cymene ligands are known to displace readily from Ru­(II) in the presence of N-based σ-donors.^35^ We observed this in related Ru-catalyzed C–H functionalizations, but found that it does not inhibit catalysis.^36^ Thus, although *p*-cymene ligated ruthenacycles seem inaccessible mechanochemically, they are unlikely to be on-cycle species in as many Ru-catalyzed C–H functionalizations as for which they have been proposed.^37^

The prevalence of indoles in bioactive molecules has inspired many approaches to their synthesis via catalytic C–H functionalization.^38^ For example, hydroarylation product **8a** has been reported to form **9** in 45% yield via Pd-catalyzed C–H amidation using a mixer mill (30 Hz over three 60 min milling periods).^33b^ Under G&H conditions (grinding 5 min, then heating at 70 °C for 2 h), we found **9** formed in 48% spectroscopic yield (34% isolated), which is within reach of the ball milling approach.

### Solventless Ir-Catalyzed C–H Borylation of Heteroarenes

Ir-catalyzed (hetero)­arene C–H borylation has grown into a profoundly enabling methodology, not least due to its functional group tolerance and the enormous versatility of the C–B bond.^39^ In what is – to the best of our knowledge – the only study on mechanochemical Ir-catalyzed C–H borylation, Ito and co-workers used ball milling to side-step the need for dry, degassed solvents or inert atmospheres.^6b,40^ Although initial optimized conditions gave several C2-borylated indoles (*e.g*., **11a**, Scheme 4), substrates with modestly higher melting points (>70 °C) gave no conversion, which was attributed to poor reagent mixing.^6b^ Using LAG^15^ (THF, 25 μL/mg reagent) increased the spectroscopic yields of **11b**, **11c** and **11d** from 0% in each case to 66%, 64% and 93%, respectively. C–H Borylation under G&H conditions (heating at 80 °C) gave us essentially identical results to those from the ball milling approach for both indole- and pyrrole-based substrates, **11a**-**c**, without the need for either air exclusion or LAG.^41^ We could also extend the scope to include lilolidine, a core substructure of numerous bioactive molecules. Its C2-boryl derivative **11e** was obtained in 57% isolated yield without LAG, despite the melting point of lilolidine (87 °C) being higher than the reaction temperature (80 °C), which contrasts with the criteria for LAG under ball milling. However, it accords with our finding for G&H C–H methylation,^17b^ for which DSC measurements revealed only the melting window for the substrate or reaction mixture needs to begin below the reaction temperature for successful conversion to occur. The G&H C–H borylation of lilolidine could be incorporated in a one-pot borylation/Suzuki-Miyaura coupling sequence (see below, Scheme 5b).^42^

**Scheme 4:**
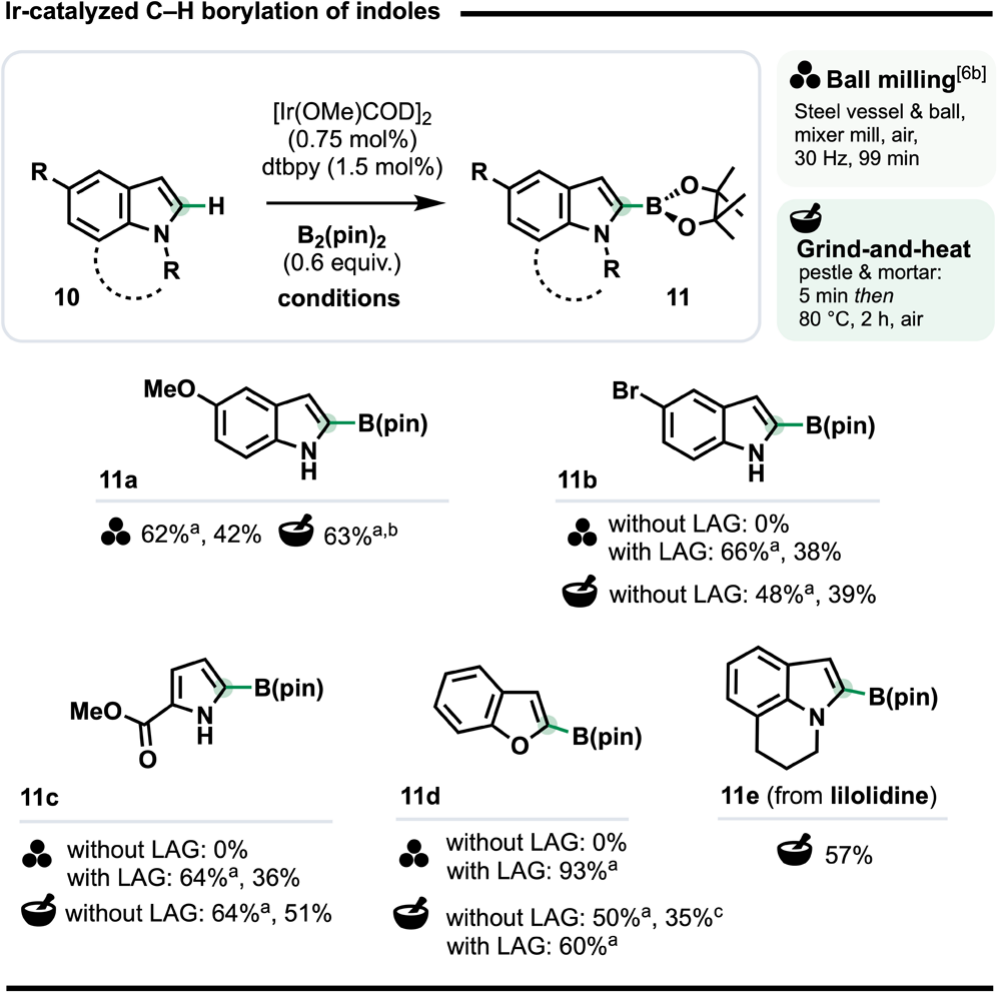
Ir-Catalyzed Heteroarene C–H Borylation^a^

a G&H at 0.3 mmol scale. dtbpy = 4,4′-di-*tert*-butyl-2,2′-bipyridyl; B2­(pin)­2 = bis­(pinacolato)­diboron. LAG (liquid-assisted grinding): THF (25 μL/mg of reagent mass) included with grinding. ^a^Yield determined by ^1^H NMR spectroscopy using 1,3,5-trimethoxybenzene as internal standard. ^b^Average of three runs by three different operators. ^c^3 mmol scale.

**Scheme 5:**
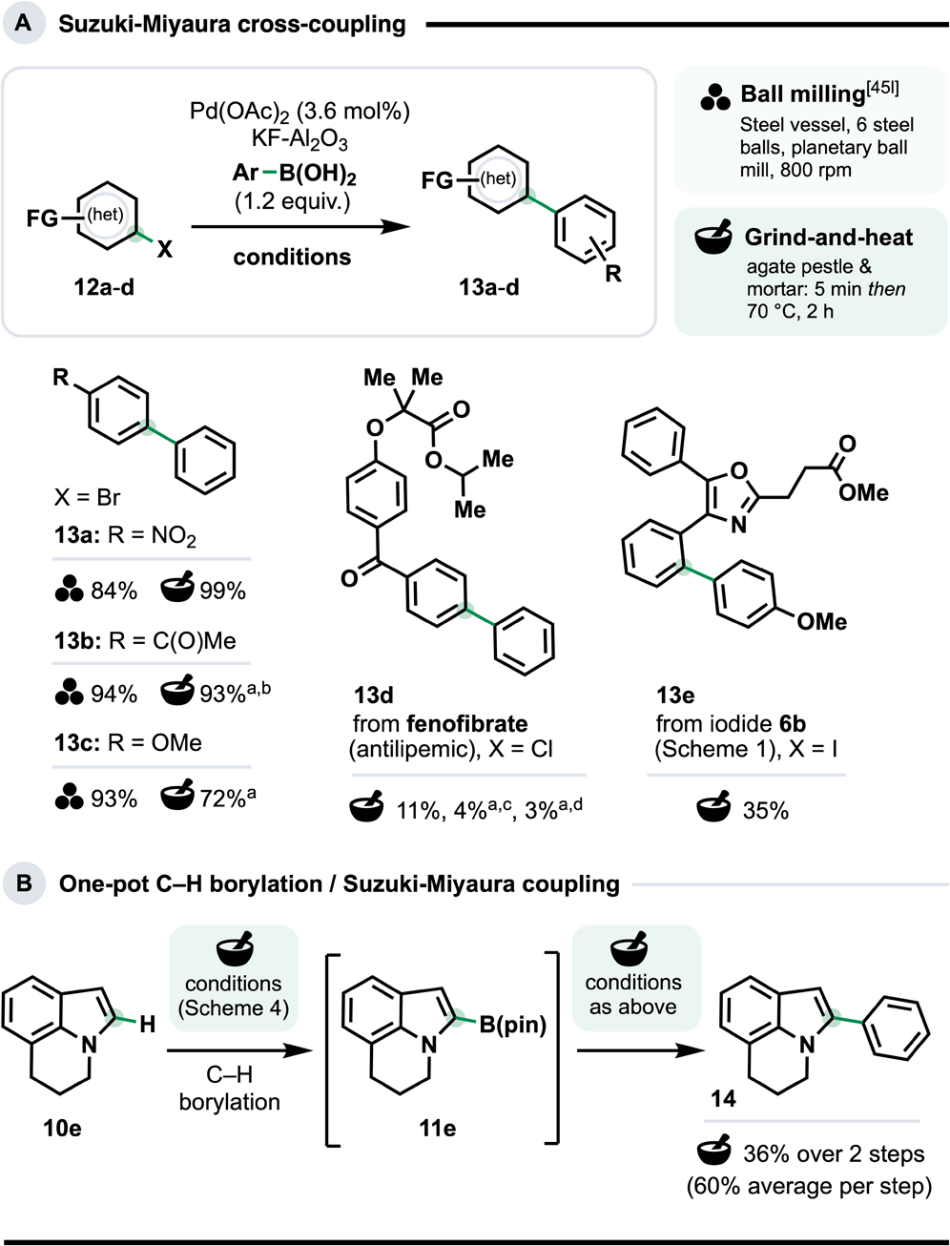
Suzuki-Miyaura cross coupling. G&H at 0.3 mmol scale[Fn sch5-fn1]

a
^a^Yield determined using ^1^H NMR spectroscopy with 1,3,5-trimethoxybenzene as an internal standard. ^b^Average of three runs by three different operators. ^c^5 min magnetic stirring (400 rpm) instead of grinding, ^d^Heated without grinding step.

### Solventless Suzuki–Miyaura Cross-Coupling

Suzuki-Miyaura cross-coupling (SMC) is the pre-eminent method for constructing biaryls,^43^ which feature in countless bioactive and functional molecules;^44^ interest in mechanochemical SMCs has grown accordingly.^4a,14c,15b,45^ A sole report by Ananikov marks the only precedent for SMC under conditions approximating G&H.^46^ However, their system was catalyzed by Pd nanoparticles supported on multiwalled carbon nanotubes (PdNPs/MWCNT). Without these to hand, we opted to examine a simpler system previously described to work under ball milling conditions: a Pd­(OAc)_2_ precatalyst with a KF-Al_2_O_3_ activator.^45l^ Initially, we compared this with G&H for SMCs between phenylboronic acid and three electrophiles: 4-nitro, 4-aceto- and 4-methoxybromobenzene (Scheme 5). Yields for our G&H approach (products **13a**–**c**, 72–99%) were competitive with those from ball milling, and tracked the electronic properties of the R groups, in line with the expected difficulty of oxidative addition.^47^ The same system could address the Ar–Cl bond of fenofibrate, a commercialized antilipemic (product **13d**), as well as the Ar–I bond of **4b** (obtained from the late-stage C–H iodination of oxaprozin – see Scheme 1B) to give **13e**. Although the yields from these two reactions were modest (11–35%), these amounts would suffice for physiochemical and biological testing.

2-Arylindoles feature in numerous bioactive molecules.^48^ We tested the prospects of constructing the C2–Ar bond via a one-pot C–H borylation/SMC sequence, starting from the privileged lilolidine scaffold (**10e**). In the first step, **10e** was addressed using our G&H C–H borylation conditions (Scheme 4). After the heating step, catalytic Pd­(OAc)_2_, KF-Al_2_O_3_ and PhB­(OH)_2_ were added to the crude mixture, which was ground (5 min), before the resumption of heating (70 °C). Product **14** was obtained in 36% isolated yield (an average of 60%/step), demonstrating the viability of one-pot G&H procedures, even involving two different transition metals.

### Solventless Buchwald–Hartwig Amination

The Buchwald-Hartwig amination (BHA) is a well-established and profoundly enabling method for installing amine functionality.^49^ Recently, several ball mill-based protocols for BHA have been described.^50^ To examine the applicability of G&H conditions to this reaction class, we adapted two sets of conditions separately reported by the teams of Geneste^50a^ (System 1, Scheme 6), and Kubota and Ito^50b^ (System 2). The former uses a Pd loading at 13 mol % loading, split equally between Pd­(OAc)­2 and tBuXPhosG3 (**16**).^50a^ The latter system uses Pd­(OAc)_2_ (5 mol %) and ^t^BuP·HBF_4_ (10 mol %), although Kubota and Ito also found XPhos to be a highly effective ligand (giving 91% yield),^50b^ which we used for this study. Maintaining the ball milling reactions at 125 °C was described as beneficial, so we used this as the temperature for the heating step of our G&H regime.

**Scheme 6:**
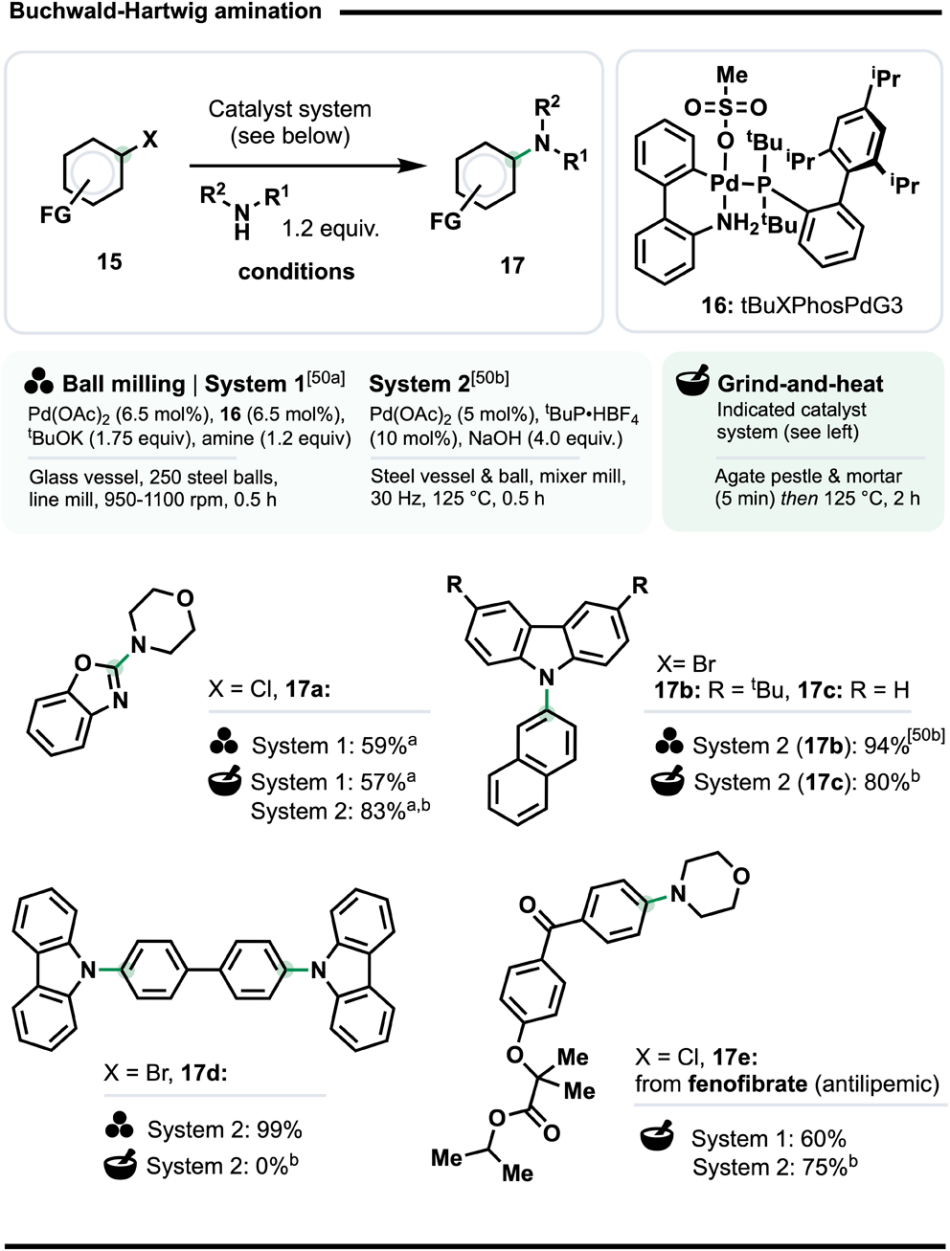
Buchwald–Hartwig Aminations^a^

a All reactions at 0.3 mmol scale. ^a^Yield determined using ^1^H NMR spectroscopy with 1,3,5-trimethoxybenzene as an internal standard. ^b^XPhos (10 mol %) used as ligand.

Initially, we compared the G&H versions of System 1 and System 2 in the amination of 2-chlorobenzoxazole with morpholine (product **17a**). Ball milling and G&H versions of System 1 gave very similar results (59% vs 57% spectroscopic yield); System 2 gave 83%. The G&H version of System 2 also gave carbazole-based diaryl amine **17c** in 80% yield from 2-bromonaphthalene, a little lower than the 94% obtained by Kubota and Ito for analog **17b**.^50b^ Unfortunately, G&H could not compete with ball milling for generating **17d**; the continuous physical impact under ball milling conditions may be better suited to overcoming π-stacking interactions in larger polycyclic aromatic molecules. Finally, we applied again the G&H-adapted Systems 1 and 2 in the late-stage amination of the antilipemic fenofibrate with morpholine, which gave **17e** in 60% and 75% yield, respectively. Overall, this selection of reactions establishes the viability of the ball mill-free G&H approach for the amination of aryl chlorides and bromides with either aromatic or aliphatic amines, as well as in the context of modifying a commercialized drug.

### General Remarks

In line with our study on solventless catalytic C–H methylation,^17b^ the G&H reactions discussed above exhibited excellent reproducibility both between different runs as well as different operators. Importantly, omitting either the grinding or heating step, using magnetic stirring instead of grinding (either prior to or during heating), or grinding the reaction components separately before their combined heating invariably led to diminished or negligible yields.^51^ In addition to providing homogenization, the grinding step of the G&H method may facilitate the formation of mechanistically relevant cocrystalline intermediates.^14^

## Conclusions

We have described an expedient, inexpensive and accessible protocol for solventless C–H and C-X functionalizations to form C–B, C–C, C–N, C–I and C–Rh bonds without a ball mill. Some of these transformations were previously reported individually to require specific atmospheres (e.g., O_2_ or Ar), lengthy milling/pausing protocols, or LAG under ball milling conditions. Although G&H struggled with larger polycyclic aromatic substrates, yields were generally competitive with previously reported ball milling conditions for all reaction classes. Additionally, we have shown G&H to be suitable for the manipulation of a variety of biologically active compounds not previously addressed under solventless conditions of any kind. Finally, G&H can also be used in a one pot fashion (i.e., without intermediate purification) even with different transition metals catalysts in each step.

Ball milling offers significant sustainability advantages, can be adapted for gaseous reagents,^6d,6e^ and is an important tool for understanding solventless reactivity. Our G&H approach does not challenge this; instead, our results show G&H has greater potential than might be assumed as an operationally simple and inexpensive approach to solventless transformations. We hope this will contribute to a reduction of reliance on bulk solvent media and, thus, the advancement of sustainable chemistry practices in organic synthesis.

## Supplementary Material



## Data Availability

The data underlying this study are available in the published article and its Supporting Information.

## Notes

General information, detailed experimental procedures, characterization data, and NMR spectra (PDF)
