# Supplementary material for: Solventless Catalytic C–H and C–X Functionalization without Ball Milling
Source: J Org Chem. 2025 Jun 19;90(26):8846–56. doi: 10.1021/acs.joc.5c00109 (PMC12235648; doi:10.1021/acs.joc.5c00109)

Solventless Catalytic C–H and C–X Functionalization Without Ball Milling

Carolina Méndez-Gálvez, Fredrik Barnå, Boris Olsthoorn, David Estébanez Rosich, Paul J. Gates and Lukasz T. Pilarski*

Supporting information

Table of Contents

GENERAL INFORMATION S3
REACTIONS & PURIFICATION S3
CHARACTERIZATION TECHNIQUES S3
ORIGIN OF SUBSTRATES S4
COMPOUNDS PREPARED USING LITERATURE PROCEDURES S4
SUBSTRATE AND REACTION SCOPE S5
RH-CATALYZED C–H IODINATION (SCHEME 1A) S5
RH-CATALYZED OXIDATIVE C–H ALKENYLATION (SCHEME 1B) S6
METALACYCLIC COMPLEXES (SCHEME 2) S9
RU-CATALYZED HYDROARYLATION OF ALKYNES (SCHEME 3) S10
INDOLE SYNTHESIS VIA PD-CATALYZED C–H AMIDATION (SCHEME 3B) S12
IR-CATALYZED C-H BORYLATION OF HETEROARENES (SCHEME 4) S13
SUZUKI MIYAURA CROSS-COUPLING (SCHEME 5A) S16
ONE POT C–H BORYLATION / SUZUKI-MIYAURA COUPLING (SCHEME 5B) S19
BUCHWALD-HARTWIG AMINATION (SCHEME 6) S20
REFERENCES S22
COPIES OF NMR SPECTRA S23

General Information

Reactions & purification

Unless stated otherwise, all reagents and solvents were obtained commercially and used as received, and all reactions were carried out under normal atmospheric conditions using an agate mortar and pestle and 10 mL microwave vials capped under air. Reactions were heated using standard laboratory heating (PEG) bath.

Thin-layer chromatography (TLC) analysis was performed using Merck TLC plates (TLC Silica gel 60, F254, aluminium sheets) and visualized using ultraviolet light (254 or 350 nm). Purification by column chromatography was performed using VWR silica gel 60 H (particle size 0.063−0.100 mm). Flash column chromatography was performed using a Biotage Isolera One Flash Chromatography instrument with Luknova silica cartridges 12 g (30 ml/min flow rate) and 25 g (35 ml/min flow rate).

Characterization techniques

Mass spectrometry: High-resolution accurate-mass mass spectra were analyzed using either a Thermo Fisher Scientific Orbitrap Elite (ESI), Waters Synapt G2-Si with Advion Nanomate (nanospray) or Thermo Fisher Scientific QExactive GC-Orbitrap (EI).

NMR spectroscopy: ^{1}H, ^{13}C and ^{19}F NMR spectra were recorded at 25 °C using a Varian Agilent MR400-DD2 400 MHz spectrometer (^{1}H 400 MHz, ^{13}C 101 MHz, ^{19}F 376 MHz), Bruker Avance 500 MHz spectrometer (^{1}H 500 MHz, ^{13}C 125 MHz) or Bruker BioSpin 600 MHz spectrometer (^{1}H 601 MHz, ^{13}C 151 MHz, ^{19}F 565 MHz).

^{1}H and ^{13}C NMR chemical shifts are reported in ppm. Structure determination of new compounds was performed using bidimensional NMR spectroscopy. ^{1}H NMR shifts were referenced indirectly to tetramethylsilane via residual solvent signals (7.26 ppm for $CDCl_3$). ^{13}C NMR shifts were referenced to the solvent peak (77.2 ppm for $CDCl_3$). ^{19}F chemical shifts were calibrated to an external standard ($CFCl_3$ at 0.00 ppm). Multiplicities are reported as: s = singlet, d = doublet, t = triplet, q = quartet, m = multiplet. ^{13}C spectra were measured with decoupling of ^{1}H. Non-product signals in the recorded NMR spectra were identified with the help of the reported chemical shifts of the common NMR impurities.[1]

All NMR spectra were processed with MestReNova software (v14.3.3). Automatic baseline correction and autophase correction were routinely applied to the spectra and zero-filling was sometimes used to increase resolution of the obtained spectra.

Origin of substrates

Unless otherwise stated (see below), all starting materials were obtained commercially and used as received without further purification.

Compounds prepared using literature procedures

- Methyl 3-(4,5-diphenyloxazol-2-yl)propanoate (Oxaprozin methyl ester, **3b**).[2]
- 1-(pyrimidin-2-yl)-1H-indole (**5a**)[3]
- 9-(pyrimidin-2-yl)-9H-carbazole (**5b**)[4]
- 2-phenoxypyridine (**5c**)[5]
- $[Cp^*RhCl_2]_2$[6]
- $[RuCl_2(p\text{-cymene})]_2$[7]

Substrate and reaction scope

Rh-catalyzed C–H iodination (Scheme 1A)

2-(2,6-di-iodophenyl)pyridine (**4a**)

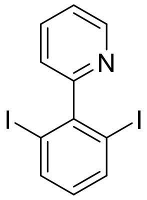

To an agate mortar were added [Cp*$RhCl_2$]$_2$ (0.01 g, 0.015 mmol, 0.05 equiv.), $AgSbF_6$ (0.041 g, 0.12 mmol, 0.4 equiv.) and NIS (0.15 g, 0.66 mmol, 2.2 equiv.). The resulting mixture was ground manually for 5 min with a pestle, then transferred into a 10 mL microwave vial and 2-phenylpyridine (0.043 mL, 0.30 mmol, 1.0 equiv.) added. The vial was subsequently capped and placed into an oil bath preheated to 90 °C. The reaction mixture was heated under air for 120 min without magnetic stirring, then cooled to r.t., washed out of the vial with a small amount of EtOAc and filtered through a tightly packed Celite plug. The plug was flushed with EtOAc (30 mL). The filtrate was washed with saturated aqueous sodium bisulfite solution (20 mL). The phases were separated, and the aqueous phase was extracted with EtOAc (20 mL). The combined organic phases were dried over Na_2SO_4 and then filtered. To the filtrate was added 1,3,5-trimethoxybenzene as a stock solution (0.50 mL; 0.1 M in EtOAc). The mixture was concentrated under reduced pressure and spectroscopic yields were determined by ^{1}H NMR spectroscopy (0.27 mmol, 91%, 36:55 monoiodination:diiodination). Spectra are in agreement with previously reported data.[8]

Methyl 3-(4-(2-iodophenyl)-5-phenyloxazol-2-yl)propanoate (**4b**)

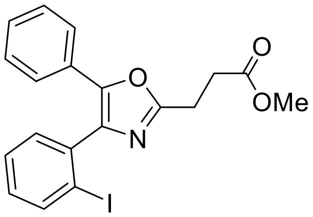

To an agate mortar were added Oxaprozin methyl ester **3b** (0.092 g, 0.30 mmol, 1.0 equiv.), [Cp*$RhCl_2$]$_2$ (0.01 g, 0.015 mmol, 0.05 equiv.), $AgSbF_6$ (0.041 g, 0.12 mmol, 0.4 equiv.) and NIS (0.15 g, 0.66 mmol, 2.2 equiv.). The resulting mixture was ground manually for 5 min with a pestle, then transferred into a 10 mL microwave vial, which was subsequently placed into an oil bath preheated to 90 °C. The reaction mixture was heated under air for 120 min without magnetic stirring, then cooled to r.t., washed out of the vial with a small amount of EtOAc and filtered through a tightly packed Celite plug. The plug was flushed with EtOAc (100 mL) and the combined filtrates were concentrated under reduced pressure. The reaction mixture was purified by flash column chromatography using a gradient of ethyl acetate in petroleum ether (0 to 100%) to afford the product as an off-white solid (0.087 g, 68%). ^{1}H NMR (400 MHz, $CDCl_3$): δ 7.97 (d, *J* = 7.9 Hz, 1H), 7.43 - 7.14 (m, 7H), 7.11 - 7.09 (m, 1H), 3.73 (s, 3H), 3.23 (t, *J* = 7.8 Hz, 2H), 2.94 (t, *J* = 7.8 Hz, 2H). $^{13}C\{^1H\}$ NMR (101 MHz, $CDCl_3$): δ 172.4, 161.1, 145.8, 139.6, 138.0, 136.9, 131.2, 130.2, 128.6, 128.4, 128.2, 128.1, 124.9, 99.8, 77.2, 52.00, 30.8, 23.5. HRMS-ESI: calcd for $C_{19}H_{16}INO_3$ $[M+H]^+$, 434.0248; found, 434.0243.

N-(2,4-difluorophenyl)-2-(2-iodo-5-(trifluoromethyl)phenoxy)nicotinamide (**4c**)

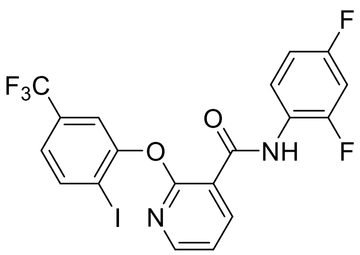

To an agate mortar were added Diflufenican (0.118 g, 0.30 mmol, 1.0 equiv.), [Cp*$RhCl_2$]$_2$ (0.01 g, 0.015 mmol, 0.05 equiv.), $AgSbF_6$ (0.041 g, 0.12 mmol, 0.4 equiv.) and NIS (0.149 g, 0.66 mmol, 2.2 equiv.). The resulting mixture was ground manually for 5 min with a pestle, then transferred into a 10 mL microwave vial, which was capped and placed into an oil bath preheated to 90 °C. The reaction mixture was heated under air for 120 min without magnetic stirring, then cooled to r.t., washed out of the vial with a small amount of EtOAc and filtered through a tightly packed Celite plug (EtOAc, 100 mL). For spectroscopic yield determination, quenching with aqueous sodium bisulfite was performed analogously to that for **4a**. For isolated yield, the filtrates were concentrated under reduced pressure and the crude purified by column chromatography using a gradient of ethyl acetate in petroleum ether (0 to 100%) to afford the product as a colorless solid (0.040 g, 26%). ^{1}H NMR (500 MHz, $CDCl_3$): δ 9.72 (s, 1H), 8.62 (dd, *J* = 7.6, 2.1 Hz, 1H), 8.40 (dt, *J* = 9.0, 6.0 Hz, 1H), 8.14 (dd, *J* = 4.7, 2.1 Hz, 1H), 7.97 (d, *J* = 8.2 Hz, 1H), 7.41 (s, 1H), 7.24 (d, *J* = 8.4 Hz, 1H), 7.21 – 7.16 (m, 1H), 6.86 - 6.78 (m, 2H) . ^{13}C{^{1}H} NMR (126 MHz, $CDCl_3$): δ 159.9, 158.8 (d, *J* = 11.7 Hz), 157.5, 156.8 (d, *J* = 10.3 Hz), 152.9 (d, *J* = 11.1 Hz), 151.8, 151.0 (d, *J* = 13.0 Hz), 149.4, 141.9, 139.8, 131.7 (q, *J* = 3.4 Hz), 123.2 (m), 122.1 (dd, *J* = 8.9, 1.7 Hz), 121.77 (dd, *J* = 11.2, 3.8 Hz), 121.1, 119.9 (q, *J* = 4.17 Hz), 119.2,115.8, 110.3 (d, *J* = 3.7 Hz), 110.4 (d, *J* = 3.9 Hz), 102.8 (d, *J* = 23.4 Hz), 102.6 (d, *J* = 22.8 Hz)^{19}F NMR (376 MHz, $CDCl_3$) δ -62.82 (s), -114.42 - -114.49 (m), -124.27 - -124.35 (m). HRMS-ESI: calcd for $C_{19}H_{10}F_5IN_2O_2$ [M+H]$^+$, 520.9780; found, 520.9778.

Rh-catalyzed oxidative C–H alkenylation (Scheme 1B)

Butyl-3-(2-acetamidophenyl) acrylate (**6a**):

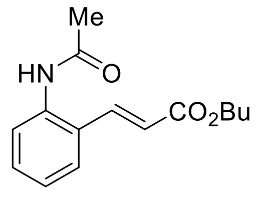

To an agate mortar were added acetanilide **5a** (0.041 g, 0.30 mmol, 1.0 equiv.), [Cp*$RhCl_2$]$_2$ (0.005 g, 0.0075 mmol, 0.025 equiv.), $AgBF_4$ (0.006 g, 0.03 mmol, 0.01 equiv.) and $Cu(OAc)_2$ (0.0014 g, 0.0075 mmol, 0.025 equiv.). The resulting mixture was ground manually for 5 min with a pestle, then transferred into a 10 mL microwave vial and butyl acrylate (0.047 mL g, 0.33 mmol, 1.1 equiv.) was added. The vial was capped and placed into an oil bath preheated to 70 °C. The reaction mixture was heated under air for 120 min without magnetic stirring, then cooled to r.t., washed out of the vial with a small amount of EtOAc and filtered through a tightly packed Celite plug. The plug was flushed with EtOAc (100 mL). To the filtrate was added 1,3,5-trimethoxybenzene as a stock solution (0.50 mL; 0.1 M in EtOAc). The mixture was concentrated under reduced pressure and spectroscopic yields were determined by ^{1}H NMR spectroscopy (0.078 mmol, 26%). Spectra are in agreement with previously reported data.[9]

N-(2-styrylphenyl)acetamide (**6b**):

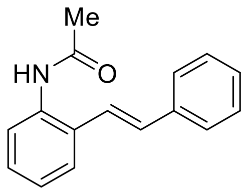

To an agate mortar were added acetanilide **5a** (0.041 g, 0.30 mmol, 1.0 equiv.), $[Cp^*RhCl_2]_2$ (0.005 g, 0.0075 mmol, 0.025 equiv.), $AgBF_4$ (0.006 g, 0.03 mmol, 0.01 equiv.) and $Cu(OAc)_2$ (0.005.2 g, 0.003 mmol, 0.010 equiv.). The resulting mixture was ground manually for 5 min with a pestle, then transferred into a 10 mL microwave vial and styrene (0.034 mL g, 0.33 mmol, 1.1 equiv.) added. The vial was subsequently placed into an oil bath preheated to 70 °C. The reaction mixture was heated under air for 120 min without magnetic stirring, then cooled to r.t., washed out of the vial with a small amount of EtOAc and filtered through a tightly packed Celite plug. The plug was flushed with EtOAc (100 mL). To the filtrate was added 1,3,5-trimethoxybenzene as a stock solution (0.50 mL; 0.1 M in EtOAc). The mixture was concentrated under reduced pressure and spectroscopic yields were determined by ^1H NMR spectroscopy (0.045 mmol, 15%). Spectra are in agreement with previously reported data.[10]

2-(2-styrylphenyl) pyridine (**6c**):

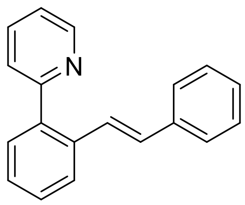

To an agate mortar were added 2-phenylpyridine (0.047 g, 0.30 mmol, 1.0 equiv.), $[Cp^*RhCl_2]_2$ (0.005 g, 0.0075 mmol, 0.025 equiv.), $AgBF_4$ (0.006 g, 0.03 mmol, 0.01 equiv.) and $Cu(OAc)_2$ (0.0014 g, 0.0075 mmol, 0.025 equiv.). The resulting mixture was ground manually for 5 min with a pestle, then transferred into a 10 mL microwave vial and styrene (0.034 mL g, 0.33 mmol, 1.1 equiv.) added. The vial was subsequently placed into an oil bath preheated to 70 °C. The reaction mixture was heated under air for 120 min without magnetic stirring, then cooled to r.t., washed out of the vial with a small amount of EtOAc and filtered through a tightly packed Celite plug. The plug was flushed with EtOAc (100 mL) and to the combined filtrates was added 1,3,5-trimethoxybenzene as a stock solution (1.0 mL; 0.1 M in DCM). The mixture was concentrated under reduced pressure and spectroscopic yields were determined by ^1H NMR spectroscopy. Spectra are in agreement with previously reported data. The reaction mixture was purified by flash column chromatography using a gradient of ethyl acetate in petroleum ether (0 to 100%) to afford the product as a colorless oil (0.025 g, 20%). Spectra are in agreement with previously reported data.[10]

4-(4-(tert-butyl)-2-ethoxy-6-styrylphenyl)-2-(2,6-difluorophenyl)-4,5-dihydrooxazole (**6d**):

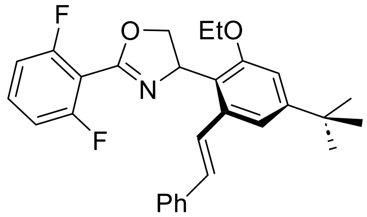

To an agate mortar were added Etoxazole (0.12 g, 0.30 mmol, 1 equiv.), $[Cp^*RhCl_2]_2$ (0.005 g, 0.0075 mmol, 0.025 equiv.), $AgBF_4$ (0.006 g, 0.03 mmol, 0.01 equiv.) and $Cu(OAc)_2$ (0.0014 g, 0.0075 mmol, 0.025 equiv.). The resulting mixture was ground manually for 5 min with a pestle, then transferred into a 10 mL microwave vial and styrene (0.034 mL g, 0.33 mmol, 1.1 equiv.) added. The vial was capped and placed into an oil bath preheated to 70 °C. The reaction mixture was heated under air for 120 min without magnetic stirring, then cooled to rt. The crude reaction mixture was washed out of the vial with a small amount of EtOAc and filtered through a tightly packed Celite plug. The plug was flushed with EtOAc (100 mL) and to the combined filtrates was added 1,3,5-

trimethoxybenzene as a stock solution (1.0 mL; 0.1 M in DCM). The mixture was concentrated under reduced pressure and spectroscopic yields were determined by ^{1}H NMR spectroscopy. The reaction mixture was purified by flash column chromatography using a gradient of ethyl acetate in petroleum ether (0 to 100%) to afford the product as a beige solid (0.010 g, 7%). ^{1}H NMR (500 MHz, $CDCl_3$): δ 7.56 (d, *J* = 15.9 Hz, 1H), 7.51 - 7.46 (m, 2H), 7.38 (tt, *J* = 8.6, 6.2 Hz, 1H), 7.32 (t, *J* = 7.6 Hz, 2H), 7.26 - 7.21 (m, 1H), 7.13 (d, *J* = 1.8 Hz, 1H), 6.96 (t, *J* = 8.4 Hz, 2H), 6.90 - 6.85 (m, 2H), 5.98 (dd, *J* = 11.3, 9.2 Hz, 1H), 4.65 (dd, *J* = 11.3, 7.8 Hz, 1H), 4.58 (dd, *J* = 9.2, 7.8 Hz, 1H), 4.08 (dtt, *J* = 16.1, 9.1, 7.0 Hz, 2H), 1.34 (m, 12H). ^{13}C{^{1}H} NMR (126 MHz, $CDCl_3$): δ 162.4 (d, *J* = 6.1 Hz), 160.3 (d, *J* = 6.1 Hz), 157.9, 156.82, 152.11, 138.7, 137.4, 132.2, 132.0, 128.5, 127.6, 126.6, 122.4, 116.7, 112.0, (d, *J* = 4.03 Hz), 111.8 (d, *J* = 4.03 Hz), 108.9, 71.8, 64.3, 63.6, 34.9, 31.3, 14.5. ^{19}F NMR (376 MHz, $CDCl_3$) δ -107.8 (t, *J* = 6.8 Hz). HRMS-ESI: calcd for $C_{29}H_{30}F_2NO_2$ $[M+H]^+$, 462.2239; found, 462.2237.

Metalacyclic complexes (Scheme 2)

Rhodacycle **7a**

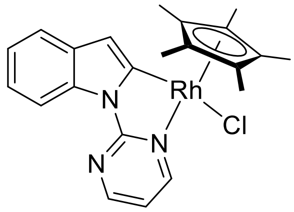

To an agate mortar were added 1-(pyrimidin-2-yl)-1H-indole (0.04 g, 0.20 mmol, 2.0 equiv.), $[RhCp^*Cl_2]_2$ (0.06 g, 0.10 mmol, 1 equiv.) and potassium acetate (0.06 g, 0.60 mmol, 6 equiv.). The resulting mixture was ground manually for 5 min with a pestle, then transferred into a 10 mL microwave vial and MeOH (40 µL, 1 mmol, 10 equiv.) was added using a micro syringe. The vial was capped and placed into an oil bath preheated to 70 °C. The reaction mixture was heated under air for 120 min without magnetic stirring, then cooled to rt, washed out of the vial with a small amount of CH_2Cl_2 and filtered. Addition of n-pentane resulted in a precipitate which was filtered and re-crystalized again from DCM/n-pentane. The solids were filtered and dried under reduced pressure to yield the product as orange/red crystals (0.031 g, 33%). Spectra are in agreement with previously reported data.[11]

Rhodacycle **7b**

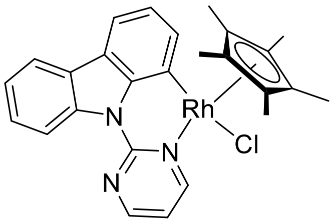

To an agate mortar were added 9-(pyrimidin-2-yl)-9H-carbazole (0.05 g, 0.20 mmol, 2 equiv.), $[RhCp^*Cl_2]_2$ (0.06 g, 0.10 mmol, 1 equiv.) and KOAc (0.06 g, 0.60 mmol, 6 equiv.). The resulting mixture was ground manually for 5 min with a pestle, then transferred into a 10 mL microwave vial and MeOH (40 µL, 1 mmol, 10 equiv.) was added via a microsyringe. The vial was capped and placed into an oil bath preheated to 70 °C. The reaction mixture was heated under air for 120 min without magnetic stirring, then cooled to rt, washed out of the vial with a small amount of CH_2Cl_2 and filtered. The product was recrystallized from DCM/n-pentane to yield the product as orange/red crystals (0.083 g, 80%). Spectra are in agreement with previously reported data.[11]

Rhodacycle **7c**

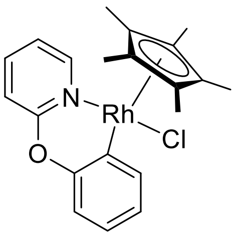

To an agate mortar were added 2-phenoxypyridine (0.04 g, 0.20 mmol, 2 equiv.), $[RhCp^*Cl_2]_2$ (0.06 g, 0.10 mmol, 1 equiv.) and potassium acetate (0.06 g, 0.60 mmol, 6 equiv.). The resulting mixture was ground manually for 5 min with a pestle, then transferred into a 10 mL microwave vial and MeOH (40 µL, 1 mmol, 10 equiv.) added. The vial was subsequently placed into an oil bath preheated to 70 °C. The reaction mixture was heated under air for 120 min without magnetic stirring, then cooled to r.t., washed out of the vial with a small amount of DCM and filtered. The product was recrystallized from DCM/n-pentane to yield the product as orange/red crystals (0.053 g, 59%). Spectra are in agreement with previously reported data.[11]

Ru-catalyzed hydroarylation of alkynes (Scheme 3)

N-(2-(1,2-diphenylvinyl)phenyl)acetamide (**8a**)

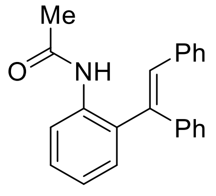

To an agate mortar were added acetanilide (0.041 g, 0.30 mmol, 1.0 equiv.), diphenyl acetylene (0.064 g, 0.36 mmol, 1.2 equiv.), $[Ru(p\text{-cymene})Cl_2]_2$ (0.010 g, 0.015 mmol, 0.05 equiv.), silver hexafluoro antimonate (0.021 g, 0.060 mmol, 0.2 equiv.) and pivalic acid (0.006 g, 0.06 mmol, 0.2 equiv.). The resulting mixture was ground manually for 5 min with a pestle, then transferred into a 10 mL microwave vial, which was capped and placed into an oil bath preheated to 70 °C. The reaction mixture was heated under air for 120 min without magnetic stirring, then cooled to rt, washed out of the vial with a small amount of EtOAc and filtered through a tightly packed celite plug. The plug was flushed with EtOAc (100 mL) and the filtrate was concentrated under reduced pressure. The reaction mixture was purified by flash column chromatography using a gradient of ethyl acetate in petroleum ether (0 to 100%) to afford the product as a colorless solid (0.053 mg, 0.17 mmol, 57%). Spectra are in agreement with previously reported data.[12]

1-(2,6-bis(1-phenylprop-1-en-2-yl)phenyl)-1H-pyrazole (**8b**)

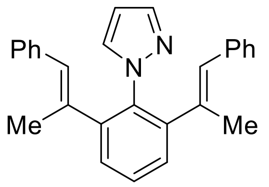

To an agate mortar were added 1-phenyl-1*H*-pyrazole (0.04 mL, 0.30 mmol, 1 equiv.), 1-phenylpropyne (0.075 mL, 0.60 mmol, 2 equiv.), $[Ru(p\text{-cymene})Cl_2]_2$ (0.009 g, 0.015 mmol, 0.05 equiv.), $AgSbF_6$ (0.021 g, 0.060 mmol, 0.2 equiv.) and PivOH (0.007 g, 0.06 mmol, 0.2 equiv.). The resulting mixture was ground manually for 5 min with a pestle, then transferred into a 10 mL microwave vial, which was capped and placed into an oil bath preheated to 60 °C. The reaction mixture was heated under air for 120 min without magnetic stirring, then cooled to rt, washed out of the vial with a small amount of EtOAc and filtered through a tightly packed Celite plug. The plug was flushed with EtOAc (100 mL) and to the combined filtrates was added 1,3,5-trimethoxybenzene as a stock solution (1.0 mL; 0.1 M in DCM). The mixture was concentrated under reduced pressure and spectroscopic yields were determined by ^{1}H NMR spectroscopy (0.23 mmol, 75%). Spectra are in agreement with previously reported data.[13]

4-(4-(tert-butyl)-2-(1,2-diphenylvinyl)-6-ethoxyphenyl)-2-(2,6-difluorophenyl)-4,5-dihydrooxazole (**8c**)

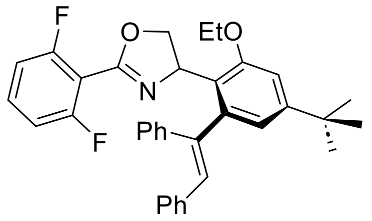

To an agate mortar were added Etoxazole (0.12 g, 0.30 mmol, 1 equiv.), diphenyl acetylene (0.064 g, 0.36 mmol, 1.2 equiv.), $[Ru(p\text{-cymene})Cl_2]_2$ (0.01 g, 0.015 mmol, 0.05 equiv.), $AgSbF_6$ (0.021 g, 0.060 mmol, 0.2 equiv.) and pivalic acid (0.006 g, 0.06 mmol, 0.2 equiv). The resulting mixture was ground manually for 5 min with a pestle, then transferred into a 10 mL microwave vial, which was capped and placed into an oil bath preheated to 90 °C. The reaction mixture was heated under air for 120 min without magnetic stirring, then cooled to rt, washed out of the vial with a small amount of EtOAc and filtered through a tightly packed Celite plug. The plug was flushed with EtOAc (100 mL) and to the combined filtrates was added 1,3,5-trimethoxybenzene as a stock solution (1.0 mL; 0.1 M in

DCM). The mixture was concentrated under reduced pressure and spectroscopic yields were determined by ^{1}H NMR spectroscopy. The reaction mixture was purified by flash column chromatography using a gradient of ethyl acetate in petroleum ether (0 to 100%) to afford the product as a beige solid (0.048 g, 31%). ^{1}H NMR (400 MHz, $CDCl_3$): δ 7.39 - 7.32 (m, 1H), 7.22 - 7.14 (m, 10H), 6.96 - 6.90 (m, 4H), 6.74 (br s, 1H), 5.58 (br s, 1H), 4.16 (br s, 1H), 4.07 - 4.01 (m, 2H), 3.66 (s, 1H), 1.32 (s, 9H), 1.24 (t, *J* = 6.9 Hz, 3H). $^{13}C\{^{1}H\}$ NMR (101 MHz, $CDCl_3$): δ 162.6 (d, *J* = 6.43 Hz), 160.0 (d, *J* = 5.6 Hz), 158.5, 156.3, 151.8, 145.4, 142.4, 137.2, 131.7, 131.6, 130.5, 129.8, 129.4, 128.3, 128.0, 127.2, 126.8, 122.5, 119.6, 112.0 - 11.9 (m), 111.8 - 111.7 (m), 108.9, 77.2, 71.2, 66.8, 63.1, 34.8, 31.3, 14.3. ^{19}F NMR (376 MHz, $CDCl_3$) δ -107.6 (q, *J* = 6.25 Hz). HRMS-ESI: calcd for $C_{35}H_{33}F_2NO_2$ $[M+H]^+$, 538.2552; found, 538.2543.

Variation 1: with magnetic stirring instead of grinding step:

When the reaction was carried out according to the above procedure except with the pestle-and-mortar grinding step replaced by magnetic stirring (400 rpm, 5 min) in a 10 mL microwave vial prior to heating, **8c** was obtained in 13% spectroscopic yield using 1,3,5-trimethoxybenzene as internal standard.

Variation 2: without grinding or stirring:

When the reaction was carried out according to the above procedure, except that the grinding or stirring step were omitted (*i.e.* all reagent components were placed in a 10 mL microwave vial and heated directly), **8c** was obtained in 16% spectroscopic yield using 1,3,5-trimethoxybenzene as internal standard.

Indole synthesis via Pd-catalyzed C–H amidation (Scheme 3B)

1-(2,3-diphenyl-1H-indol-1-yl)ethenone (**9**)

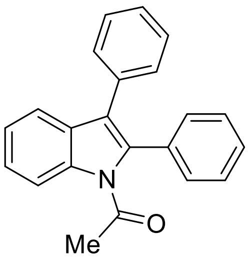

To an agate mortar were added acetamide **8a** (0.052 g, 0.16 mmol, 1.0 equiv.), $Pd(OAc)_2$ (0.0041 g, 0.018 mmol, 0.1 equiv.) and $PhI(OAc)_2$ (0.116 g, 0.36 mmol, 2.2 equiv.). The resulting mixture was ground manually for 5 min with a pestle, then transferred into a 10 mL microwave vial, which was capped and placed into an oil bath preheated to 70 °C. The reaction mixture was heated under air for 120 min without magnetic stirring, then cooled to rt, washed out of the vial with a small amount of EtOAc and filtered through a tightly packed Celite plug. The plug was flushed with EtOAc (100 mL) and to the combined filtrates was added 1,3,5-trimethoxybenzene as a stock solution (1.0 mL; 0.1 M in DCM). The mixture was concentrated under reduced pressure and spectroscopic yields were determined by ^1H NMR spectroscopy. The reaction mixture was purified by flash column chromatography using a gradient of ethyl acetate in petroleum ether (0 to 100%) to afford the product as a colorless oil (0.032 g, 34%). Spectra are in agreement with previously reported data.[12]

Ir-catalyzed C-H borylation of heteroarenes (Scheme 4)

5-methoxy-2-(4,4,5,5-tetramethyl-1,3,2-dioxaborolan-2-yl)-1H-indole (**11a**)

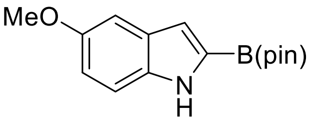

To an agate mortar were added 5-methoxyindole (0.044 g, 0.30 mmol, 1.0 equiv.), $B_2(pin)_2$ (0.046 g, 0.018 mmol, 0.6 equiv.), $[Ir(OMe)(1,5\text{-}cod)]_2$ (0.002 g, 0.0075 equiv.), and 4,4’-di-*tert*-butyl-2,2’-bipyridyl (0.001 g, 0.015 equiv.). The resulting mixture was ground manually for 5 min with a pestle, then transferred into a 10 mL microwave vial, which was capped and placed into an oil bath preheated to 80 °C. The reaction mixture was heated under air for 120 min without magnetic stirring, then cooled to rt, washed out of the vial with a small amount of EtOAc and filtered through a tightly packed Celite plug. The plug was flushed with EtOAc (100 mL) and to the combined filtrates was added 1,3,5-trimethoxybenzene as a stock solution (1.0 mL; 0.1 M in DCM). The mixture was concentrated under reduced pressure and spectroscopic yields were determined by ^{1}H NMR spectroscopy. Spectra are in agreement with previously reported data.[14]

Average of three runs with grinding step carried out by different operators:

Reaction	Person	Spectroscopic yield (%)
1	FB	64
2	LP	63
3	MP	63

5-bromo-2-(4,4,5,5-tetramethyl-1,3,2-dioxaborolan-2-yl)-1H-indole (**11b**)

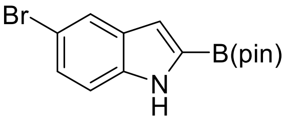

To an agate mortar were added 5-bromoindole (0.059, 0.30 mmol, 1.0 equiv.), $B_2(pin)_2$ (0.046 g, 0.018 mmol, 0.6 equiv.), $[Ir(OMe)(1,5\text{-}cod)]_2$ (0.002 g, 0.0075 equiv.), and 4,4’-di-*tert*-butyl-2,2’-bipyridyl (0.001 g, 0.015 equiv.). The resulting mixture was ground manually for 5 min with a pestle, then transferred into a 10 mL microwave vial, which was capped and placed into an oil bath preheated to 80 °C. The reaction mixture was heated under air for 120 min without magnetic stirring, then cooled to rt, washed out of the vial with a small amount of EtOAc and filtered through a tightly packed Celite plug. The plug was flushed with EtOAc (100 mL) and to the combined filtrates was added 1,3,5-trimethoxybenzene as a stock solution (1.0 mL; 0.1 M in DCM). The mixture was concentrated under reduced pressure and spectroscopic yields were determined by ^{1}H NMR spectroscopy. The crude mixture was purified by flash column chromatography using a gradient of ethyl acetate in petroleum ether (0 to 100%) to afford the product as a colorless oil in (0.035 g, 39%). Spectra are in agreement with previously reported data. [14]

Methyl 5-(4,4,5,5-tetramethyl-1,3,2-dioxaborolan-2-yl)-pyrrole-2-carboxylate (**11c**)

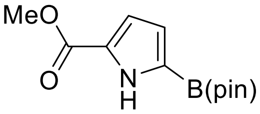

To an agate mortar were added methyl pyrrole-2-carboxylate (0.037, 0.30 mmol, 1.0 equiv.), $B_2(pin)_2$ (0.046 g, 0.018 mmol, 0.6 equiv.), $[Ir(OMe)(1,5\text{-}cod)]_2$ (0.002 g, 0.0075 equiv.), and 4,4’-di-*tert*-butyl-2,2’-bipyridyl (0.001 g, 0.015 equiv.). The resulting mixture was ground

manually for 5 min with a pestle, then transferred into a 10 mL microwave vial, which was capped and placed into an oil bath preheated to 80 °C. The reaction mixture was heated under air for 120 min without magnetic stirring, then cooled to rt, washed out of the vial with a small amount of EtOAc and filtered through a tightly packed Celite plug. The plug was flushed with EtOAc (100 mL) and to the combined filtrates was added 1,3,5-trimethoxybenzene as a stock solution (1.0 mL; 0.1 M in DCM). The mixture was concentrated under reduced pressure and spectroscopic yields were determined by ^{1}H NMR spectroscopy. The crude mixture was purified by flash column chromatography using a gradient of ethyl acetate in petroleum ether (0 to 100%) to afford the product as a beige solid (0.039 g, 51%). Spectra are in agreement with previously reported data.[14]

2-(benzofuran-2-yl)-4,4,5,5-tetramethyl-1,3,2-dioxaborolane (**11d**)

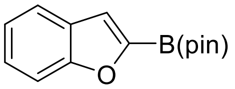

To an agate mortar were added 2,3-benzofuran (0.035 g, 0.30 mmol, 1.0 equiv.), B_2(pin)$_2$ (0.046 g, 0.018 mmol, 0.6 equiv.), [Ir(OMe)(1,5-cod)]$_2$ (0.002 g, 0.0075 equiv.), and btbpy (0.001 g, 0.015 equiv.). The resulting mixture was ground manually for 5 min with a pestle, then transferred into a 10 mL microwave vial, which was subsequently placed into an oil bath preheated to 80 °C. The reaction mixture was heated under air for 120 min without magnetic stirring, then cooled to r.t., washed out of the vial with a small amount of EtOAc and filtered through a tightly packed Celite plug. The plug was flushed with EtOAc (100 mL) and to the combined filtrates was added 1,3,5-trimethoxybenzene as a stock solution (1.0 mL; 0.1 M in DCM). The mixture was concentrated under reduced pressure and spectroscopic yields were determined by ^{1}H NMR spectroscopy. The crude mixture was purified by flash column chromatography using a gradient of ethyl acetate in petroleum ether (0 to 100%) to afford the product as a colorless solid (0.033 g, 45%). Spectra are in agreement with previously reported data.[14]

When the reaction was scaled up to 3.0 mmol 2,3-benzofuran, the procedure was the same, apart from that the crude mixture was recrystallized from hexanes to afford the product as a colorless solid (257.1 mg, 35%).

2-(4,4,5,5-tetramethyl-1,3,2-dioxaborolan-2-yl)-5,6-dihydro-4H-pyrrolo[3,2,1-ij] quinoline (**11e**)

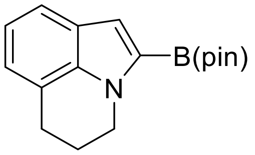

To an agate mortar were added Lilolidine (0.047 g, 0.30 mmol, 1.0 equiv.), B_2(pin)$_2$ (0.046 g, 0.018 mmol, 0.6 equiv.), [Ir(OMe)(1,5-cod)]$_2$ (0.002 g, 0.0075 equiv.), and btbpy (0.001 g, 0.015 equiv.). The resulting mixture was ground manually for 5 min with a pestle, then transferred into a 10 mL microwave vial, which was subsequently placed into an oil bath preheated to 80 °C. The reaction mixture was heated under air for 120 min without magnetic stirring, then cooled to r.t., washed out of the vial with a small amount of EtOAc and filtered through a tightly packed Celite plug. The plug was flushed with EtOAc (100 mL), and the combined filtrates were concentrated under reduced pressure. The reaction mixture was purified by flash column chromatography using a gradient of ethyl acetate in petroleum ether (0 to 100%) to afford the product as a yellow oil (0.048 g, 57%). ^{1}H NMR (400 MHz, $CDCl_3$): δ 7.46 (d, *J* = 7.3 Hz, 1H), 7.05 (s, 1H), 7.04 - 6.91 (m, 2H), 4.39 (t, *J* = 9.2 Hz, 2H), 3.02 (m, 2H), 2.28 (m, 2H), 1.36 (s, 12H). ^{13}C{^{1}H} NMR (101 MHz, $CDCl_3$): δ 137.4, 126.0, 125.6, 122.4, 119.7,

119.6, 118.8, 113.0, 100.3, 83.5, 44.4, 24.9, 24.8, 23.1. HRMS-ESI: calcd for $C_{17}H_{22}BNO_2$ $[M+H]^+$, 284.1816; found, 284.1812.

Suzuki Miyaura cross-coupling (Scheme 5A)

4-nitro-1,1'-biphenyl (**13a**)

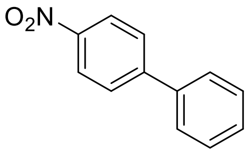

To an agate mortar were added 1-bromo-4-nitrobenzene (0.061 g, 0.3 mmol, 1.0 equiv.), phenylboronic acid (0.048 g, 0.38 mmol, 1.25 equiv.), $Pd(OAc)_2$ (0.0027 g, 0.012 mmol, 0.04 equiv.) and KF/Al_2O_3 (0.3 g). The resulting mixture was ground manually for 5 min with a pestle, then transferred into a 10 mL microwave vial, which was capped and placed into an oil bath preheated to 70 °C. The reaction mixture was heated under air for 120 min without magnetic stirring, then cooled to r.t., washed out of the vial with a small amount of EtOAc and filtered through a tightly packed Celite plug. The plug was flushed with EtOAc (100 mL). The filtrate was concentrated under reduced to afford the product as a yellow solid (59.7 mg, 0.299 mmol, 99%). Spectroscopic data were in agreement with previously reported values.[15]

4´-phenylacetophenone (**13b**)

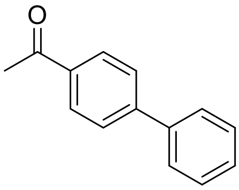

To an agate mortar were added 4´-Bromoacetophenone (0.060 g, 0.3 mmol, 1.0 equiv.), phenylboronic acid (0.046 g, 0.38 mmol, 1.25 equiv.), $Pd(OAc)_2$ (0.0027 g, 0.012 mmol, 0.04 equiv.) and KF/Al_2O_3 (0.3 g). The resulting mixture was ground manually for 5 min with a pestle, then transferred into a 10 mL microwave vial, which was subsequently placed into an oil bath preheated to 70 °C. The reaction mixture was heated under air for 120 min without magnetic stirring, then cooled to r.t., washed out of the vial with a small amount of EtOAc and filtered through a tightly packed Celite plug. The plug was flushed with EtOAc (100 mL) and to the combined filtrates was added 1,3,5-trimethoxybenzene as a stock solution (1.0 mL; 0.1 M in DCM). The mixture was concentrated under reduced pressure and spectroscopic yields were determined by ^{1}H NMR spectroscopy. Spectroscopic data were in agreement with previously reported values.[16]

Average of three runs with grinding step carried out by different operators:

Reaction	Person	Spectroscopic yield (%)
1	FB	96
2	LP	89
3	MP	95

4-methoxy-1,1´-biphenyl (**13c**)

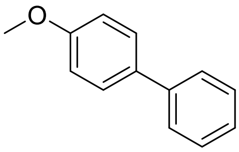

To an agate mortar were added phenylboronic acid (0.048 g, 0.38 mmol, 1.25 equiv.), $Pd(OAc)_2$ (0.0027 g, 0.012 mmol, 0.04 equiv.) and KF/Al_2O_3 (0.3 g). The resulting mixture was ground manually for 5 min with a pestle, then transferred into a 10 mL microwave vial and 4-bromoanisole (0.056 g, 0.3 mmol, 1.0 equiv.) added. The vial was subsequently placed into an oil bath preheated to 70 °C. The reaction mixture was heated under air for 120 min

without magnetic stirring, then cooled to r.t., washed out of the vial with a small amount of EtOAc and filtered through a tightly packed Celite plug. The plug was flushed with EtOAc (100 mL) and to the combined filtrates was added 1,3,5-trimethoxybenzene as a stock solution (1.0 mL; 0.1 M in DCM). The mixture was concentrated under reduced pressure and spectroscopic yields were determined by ^{1}H NMR spectroscopy (0.22 mmol, 72%). Spectroscopic data were in agreement with previously reported values.[17]

Isopropyl 2-(4-([1,1'-biphenyl]-4-carbonyl)phenoxy)-2-methylpropanoate (**13d**)

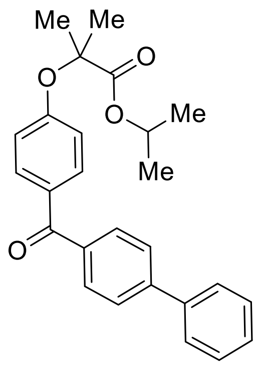

To an agate mortar were added Fenofibrate (0.108 g, 0.3 mmol, 1.0 equiv.), phenylboronic acid (0.045 g, 0.38 mmol, 1.25 equiv.), $Pd(OAc)_2$ (0.0027 g, 0.012 mmol, 0.04 equiv.) and KF/Al_2O_3 32 wt% (0.3 g). The resulting mixture was ground manually for 5 min with a pestle, then transferred into a 10 mL microwave vial, which was capped and placed into an oil bath preheated to 70 °C. The reaction mixture was heated under air for 120 min without magnetic stirring, then cooled to rt, washed out of the vial with a small amount of EtOAc and filtered through a tightly packed Celite plug. The plug was flushed with EtOAc (100 mL), and the filtrate was concentrated under reduced pressure. The reaction mixture was purified by column chromatography using 3% EtOAc in n-pentane to afford the product as a colourless solid (11%). ^{1}H NMR (400 MHz, CDCl3): δ 7.82 (m, 4H), 7.73 - 7.63 (m, 4H), 7.50 - 7. 46 (m, 2H), 7.42 - 7.39 (m, 1H), 6.88 (d, J = 8.9 Hz, 2H), 5.10 (hep, J = 6.2 Hz, 1H), 1.67 (s, 6H), 1.21 (d, J = 6.3 Hz, 6H). ^{13}C{^{1}H} NMR (101 MHz, $CDCl_3$): δ 195.1, 173.1, 159.5, 144.7, 140.0, 136.7, 132.0, 130.7, 130.4, 128.9, 128.0, 127.2, 126.8, 117.1, 79.3, 69.3, 25.3, 21.5. HRMS-ESI: calcd for $C_{26}H_{26}O_4$ $[M+H]^+$, 403.1904; found, 403.1900.

Variation 1: with magnetic stirring instead of grinding step:
When the reaction was carried out according to the above procedure except with the pestle-and-mortar grinding step replaced by magnetic stirring (400 rpm, 5 min) in a 10 mL microwave vial prior to heating, formation of Pd black was observed on the walls of the reaction vessel. **13d** was obtained in 4% spectroscopic yield using 1,3,5-trimethoxybenzene as internal standard.

Variation 2: without grinding or stirring:
When the reaction was carried out according to the above procedure, except that the grinding or stirring step were omitted (*i.e.* all reagent components were placed in a 10 mL microwave vial and heated directly), formation of Pd black was observed on the walls of the reaction vessel. **13d** was obtained in 3% spectroscopic yield using 1,3,5-trimethoxybenzene as internal standard.

Methyl 3-(4-(4'-methoxy-[1,1'-biphenyl]-2-yl)-5-phenyloxazol-2-yl)propanoate (**13e**)

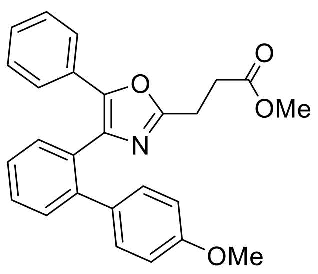

To an agate mortar were added previously synthesized Oxaproxin derivative **4b** (0.13 g, 0.3 mmol, 1.0 equiv.), 4-methoxyphenylboronic acid (0.09 g, 0.6 mmol, 2 equiv.), palladium acetate (0.0024 g, 0.012 mmol, 0.036 equiv.) and KF/Al_2O_3 32 wt% (0.3 g). The resulting mixture was ground manually for 5 min with a pestle, then transferred into a 10 mL microwave vial, which was subsequently placed into an oil bath preheated to 70 °C without magnetic stirring, for 120 min. The reaction mixture was cooled to rt, washed out of the vial with a small amount of EtOAc and filtered through a tightly packed Celite plug. The plug was flushed with additional EtOAc (100 mL), and the combined filtrates were concentrated under reduced pressure. Purification by flash column chromatography using a gradient of ethyl acetate in petroleum ether (0 to 100%) gave title compound as a colourless solid (0.043 g, 35%). ^{1}H NMR (400 MHz, $CDCl_3$): δ 7.50 - 7.36 (m, 4H), 7.20 - 7.14 (m, 5H), 6.94 (d, *J* = 8.8 Hz, 2H), 6.65 (d, *J* = 8.5 Hz, 2H), 3.73 (s, 3H), 3.71 (s, 3H), 3.15 (t, *J* = 7.4 Hz, 2H), 2.86 (t, *J* = 7.4 Hz, 2H). ^{13}C{^{1}H} NMR (101 MHz, $CDCl_3$): δ 172.4, 161.1, 158.2, 145.8, 141.8, 134.9, 133.2, 131.1, 130.8, 130.2, 130.0, 128.8, 128.3, 128.2, 127.6, 127.2, 125.0, 113.0, 55.1, 51.8, 30.9, 23.4. HRMS-ESI: calcd for $C_{26}H_{23}NO_4$ $[M+H]^+$, 414.1693; found, 414.1700.

One pot C–H borylation / Suzuki-Miyaura coupling (Scheme 5B)

2-phenyl-5,6-dihydro-4H-pyrrolo[3,2,1]quinoline (**14**)

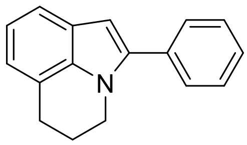

To an agate mortar were added Lilolidine (0.047 g, 0.30 mmol, 1.0 equiv.), $B_2(pin)_2$ (0.046 g, 0.018 mmol, 0.6 equiv.), $[Ir(OMe)(1,5\text{-}cod)]_2$ (0.002 g, 0.0075 equiv.), and btbpy (0.001 g, 0.015 equiv). The resulting mixture was ground manually for 5 min with a pestle, then transferred into a 10 mL microwave vial, which was subsequently placed into an oil bath preheated to 80 °C. The reaction mixture was heated under air for 120 min without magnetic stirring. The reaction mixture was cooled to rt and a previously 5 min ground mixture of $Pd(OAc)_2$ (0.0024 g, 0.012 mmol, 0.036 equiv.) and KF/Al_2O_3 32 wt% (0.3 g) was added, subsequently iodobenzene (0.06 g, 0.30 mmol, 0.5 equiv.) was added to the vial which was subsequently placed into an oil bath preheated to 70 °C without magnetic stirring, for 1120 min. The reaction mixture was cooled to rt, washed out of the vial with a small amount of EtOAc and filtered through a tightly packed Celite plug. The plug was flushed with additional EtOAc (100 mL), and the filtrate was concentrated under reduced pressure. Purification by flash column chromatography using a gradient of ethyl acetate in petroleum ether (0 to 100%) gave title compound as a light brown solid (0.025 g, 36 %). ^1H NMR (500 MHz, $CDCl_3$): δ 7.56 (d, *J* = 6.6 Hz, 2H), 7.48 – 7.45 (m, 3H), 7.38 (t, *J* = 7.4 Hz, 1H), 7.07 (t, *J* = 8.02 Hz, 1H), 6.94 (d, *J* = 7.2 Hz, 1H), 6.56 (s, 1H), 4.26 - 4.18 (m, 2H), 3.03 (t, *J* = 6.3 Hz, 2H), 2.22 (qt, *J* = 6.2 Hz, 2H). $^{13}C\{^1H\}$ NMR (126 MHz, $CDCl_3$): δ 139.9, 135.30, 132.7, 128.6, 128.5, 127.6, 125.9, 122.0, 119.8, 118.6, 117.8, 100.5, 43.7, 25.0, 23.2. HRMS-EI: calcd for $C_{17}H_{15}N$ $[M]^+$, 233.1204 found, 233.1199.

Buchwald-Hartwig amination (Scheme 6)

2-morpholinobenzoxazole (**17a**)

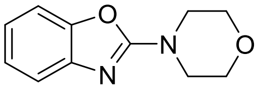

Adaptation of System 1: To an agate mortar were added benzoxazole (0.046 g, 0.3 mmol, 1.0 equiv.), $Pd(OAc)_2$ (0.004 g, 0.02 mmol, 0.065 equiv.), tBuXPhoSPdG3 (0.015 g, 0.02 mmol, 0.065 equiv.) and tBuOK (0.06 g, 0.52 mmol, 1.75 equiv.). The resulting mixture was ground manually for 5 min with a pestle, then transferred into a 10 mL microwave vial and morpholine (0.031 g, 0.36 mmol, 1.2 equiv.) added. The vial was capped and placed into an oil bath preheated to 70 °C without magnetic stirring, for 120 min. The reaction mixture was cooled to rt, washed out of the vial with a small amount of EtOAc and filtered through a tightly packed Celite plug. The plug was flushed with additional EtOAc (100 mL), and the filtrate was concentrated under reduced pressure. Purification by flash column chromatography using a gradient of ethyl acetate in petroleum ether (0 to 100%) gave title compound as an off-white solid (0.035 g, 57%).

Adaptation of System 2: To an agate mortar were added benzoxazole (0.046 g, 0.3 mmol, 1.0 equiv.), morpholine (0.031 g, 0.36 mmol, 1.2 equiv.), $Pd(OAc)_2$ (0.003 g, 0.015 mmol, 0.05 equiv.), XPhos (0.014 g, 0.03 mmol, 0.01 equiv.), and NaOH (0.05 g, 1.2 mmol, 4 equiv.). The resulting mixture was ground manually for 5 min with a pestle, then transferred into a 10 mL microwave vial, which was subsequently placed into an oil bath preheated to 125 °C without magnetic stirring, for 120 min. The reaction mixture was cooled to rt, washed out of the vial with a small amount of EtOAc and filtered through a tightly packed Celite plug. The plug was flushed with additional EtOAc (100 mL), and the filtrate was concentrated under reduced pressure. Purification by flash column chromatography using a gradient of ethyl acetate in petroleum ether (0 to 100%) gave title compound as an off white solid (0.050 g, 83%). Spectra are in agreement with previously reported data.[18]

9-(2-naphthalenyl)-9*H*-carbazole (**17c**)

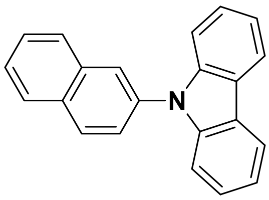

Adaptation of System 2: To an agate mortar were added 2-bromonaphtalene (0.062 g, 0.3 mmol, 1.0 equiv.), carbazole (0.05 g, 0.3 mmol, 1.0 equiv.), $Pd(OAc)_2$ (0.003 g, 0.015 mmol, 0.05 equiv.), XPhos (0.014 g, 0.03 mmol, 0.01 equiv.), and NaOH (0.05 g, 1.2 mmol, 4 equiv.). The resulting mixture was ground manually for 5 min with a pestle, then transferred into a 10 mL microwave vial, which was subsequently placed into an oil bath preheated to 125 °C without magnetic stirring, for 120 min. The reaction mixture was cooled to rt, washed out of the vial with a small amount of EtOAc and filtered through a tightly packed Celite plug. The plug was flushed with additional EtOAc (100 mL), and the filtrate was concentrated under reduced pressure. Purification by flash column chromatography using a gradient of ethyl acetate in petroleum ether (0 to 100%) gave title compound as an off-white solid (0.070 g, 80%). Spectra are in agreement with previously reported data.[19]

Isopropyl 2-methyl-2-(4-(4-morpholinobenzoyl)phenoxy)propanoate (**17e**)

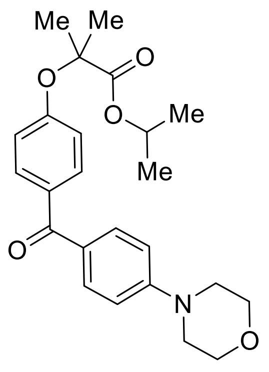

Adaptation of System 1: To an agate mortar were added Fenofibrate (0.108 g, 0.3 mmol, 1.0 equiv.), $Pd(OAc)_2$ (0.004 g, 0.02 mmol, 0.065 equiv.), $tBuXPhoSPdG_3$ (0.015 g, 0.02 mmol, 0.065 equiv.), and tBuOK (0.06 g, 0.52 mmol, 1.75 equiv.). The resulting mixture was ground manually for 5 min with a pestle, then transferred into a 10 mL microwave vial and morpholine (0.031 g, 0.36 mmol, 1.2 equiv.) added. The vial was subsequently placed into an oil bath preheated to 70 °C without magnetic stirring, for 120 min. The reaction mixture was cooled to rt, washed out of the vial with a small amount of EtOAc and filtered through a tightly packed Celite plug. The plug was flushed with additional EtOAc (100 mL), and the combined filtrates were concentrated under reduced pressure. The reaction mixture was purified by flash column chromatography using a gradient of ethyl acetate in petroleum ether (0 to 100%) to afford the product as a light green solid (0.074 g, 60%).

Adaptation of System 2: To an agate mortar were added Fenofibrate (0.108 g, 0.3 mmol, 1.0 equiv.), $Pd(OAc)_2$ (0.003 g, 0.015 mmol, 0.05 equiv.), XPhos (0.014 g, 0.03 mmol, 0.01 equiv.), and NaOH (0.05 g, 1.2 mmol, 4 equiv.). The resulting mixture was ground manually for 5 min with a pestle, then transferred into a 10 mL microwave vial and morpholine (0.031 g, 0.36 mmol, 1.2 equiv.) added. The vial was capped and placed into an oil bath preheated to 125 °C without magnetic stirring, for 120 min. The reaction mixture was cooled to rt, washed out of the vial with a small amount of EtOAc and filtered through a tightly packed Celite plug. The plug was flushed with additional EtOAc (100 mL), and the filtrate was concentrated under reduced pressure. The reaction mixture was purified by flash column chromatography using a gradient of ethyl acetate in petroleum ether (0 to 100%) to afford the product as a light green solid (0.098 g, 75%). ^{1}H NMR (400 MHz, $CDCl_3$): δ 7.76 (d, *J* = 8.1 Hz, 2H), 7.71 (d, *J* = 8.6 Hz, 2H), 6.89 (d, *J* = 8.6 Hz, 2H), 6.86 (d, *J* = 9.1 Hz, 2H), 5.08 (hept, *J* = 5.8 Hz, 1H), 3.87 - 3.84 (m, 4H), 3.32 - 3.29 (m, 4H), 1.64 (s, 6H), 1.20 (d, 6.5 Hz, 6H). $^{13}C\{^{1}H\}$ NMR (101 MHz, $CDCl_3$): δ 194.2, 173.4, 158.9, 153.9, 132.2, 131.7, 131.6, 128.4, 117.2, 113.3, 79.3, 69.3, 66.7, 47.7, 21.6, 21.1 HRMS-ESI: calcd for $C_{24}H_{29}NO_5$ $[M+H]^+$, 412.2118; found, 412.2114

References

[1] G. R. Fulmer, A. J. M. Miller, N. H. Sherden, H. E. Gottlieb, A. Nudelman, B. M. Stoltz, J. E. Bercaw, K. I. Goldberg, *Organometallics* **2010**, *29*, 2176-2179.

[2] M. Hribersek, C. Méndez-Gálvez, M. Huber, P. J. Gates, P. Shakari, A. Samanta, L. T. Pilarski, *Green Chem.* **2023**, *25*, 9138-9145.

[3] H. Wu, T. Liu, M. Cui, Y. Li, J. Jian, H. Wang, Z. Zeng, *Org. Biomol. Chem.* **2017**, *15*, 536-540.

[4] L. Zhu, X. Cao, R. Qiu, T. Iwasaki, V. P. Reddy, X. Xu, S.-F. Yin, N. Kambe, *RSC Adv.* **2015**, *5*, 39358-39365.

[5] J. Yao, R. Feng, Z. Wu, Z. Liu, Y. Zhang, *Adv. Synth. Catal.* **2013**, *355*, 1517-1522.

[6] M. Barday, C. Janot, N. R. Halcovitch, J. Muir, C. Aïssa, *Angew. Chem. Int. Ed.* **2017**, *56*, 13117-13121.

[7] S. B. Jensen, S. J. Rodger, M. D. Spicer, *J. Organomet. Chem.* **1998**, *556*, 151-158.

[8] N. Schröder, J. Wencel-Delord, F. Glorius, *J. Am. Chem. Soc.* **2012**, *134*, 8298-8301.

[9] G. N. Hermann, P. Becker, C. Bolm, *Angew. Chem. Int. Ed.* **2015**, *54*, 7414-7417.

[10] N. Umeda, K. Hirano, T. Satoh, M. Miura, *J. Org. Chem.* **2009**, *74*, 7094-7099.

[11] S. Ni, M. Hribersek, S. K. Baddigam, F. J. L. Ingner, A. Orthaber, P. J. Gates, L. T. Pilarski, *Angew. Chem. Int. Ed.* **2021**, *60*, 6660-6666.

[12] H. Cheng, J. G. Hernández, C. Bolm, *Org. Lett.* **2017**, *19*, 6284-6287.

[13] Bhawani, V. N. Shinde, Sonam, K. Rangan, A. Kumar, *J. Org. Chem.* **2022**, *87*, 5994-6005.

[14] Y. Pang, T. Ishiyama, K. Kubota, H. Ito, *Chem. Eur. J.* **2019**, *25*, 4654-4659.

[15] H. Yang, X. Han, Z. Ma, R. Wang, J. Liu, X. Ji, *Green Chem.* **2010**, *12*, 441-451.

[16] M. Keller, A. Hameau, G. Spataro, S. Ladeira, A.-M. Caminade, J.-P. Majoral, A. Ouali, *Green Chem.* **2012**, *14*, 2807-2815.

[17] L. Noël-Duchesneau, N. Lugan, G. Lavigne, A. Labande, V. César, *Organometallics* **2014**, *33*, 5085-5088.

[18] T. Guntreddi, B. K. Allam, K. N. Singh, *RSC Adv.* **2013**, *3*, 9875-9880.

[19] Y. Nakayama, N. Yokoyama, H. Nara, T. Kobayashi, M. Fujiwhara, *Adv. Synth. Catal.* **2015**, *357*, 2322-2330.

Copies of NMR spectra

^{1}H NMR 400 MHz **4b**

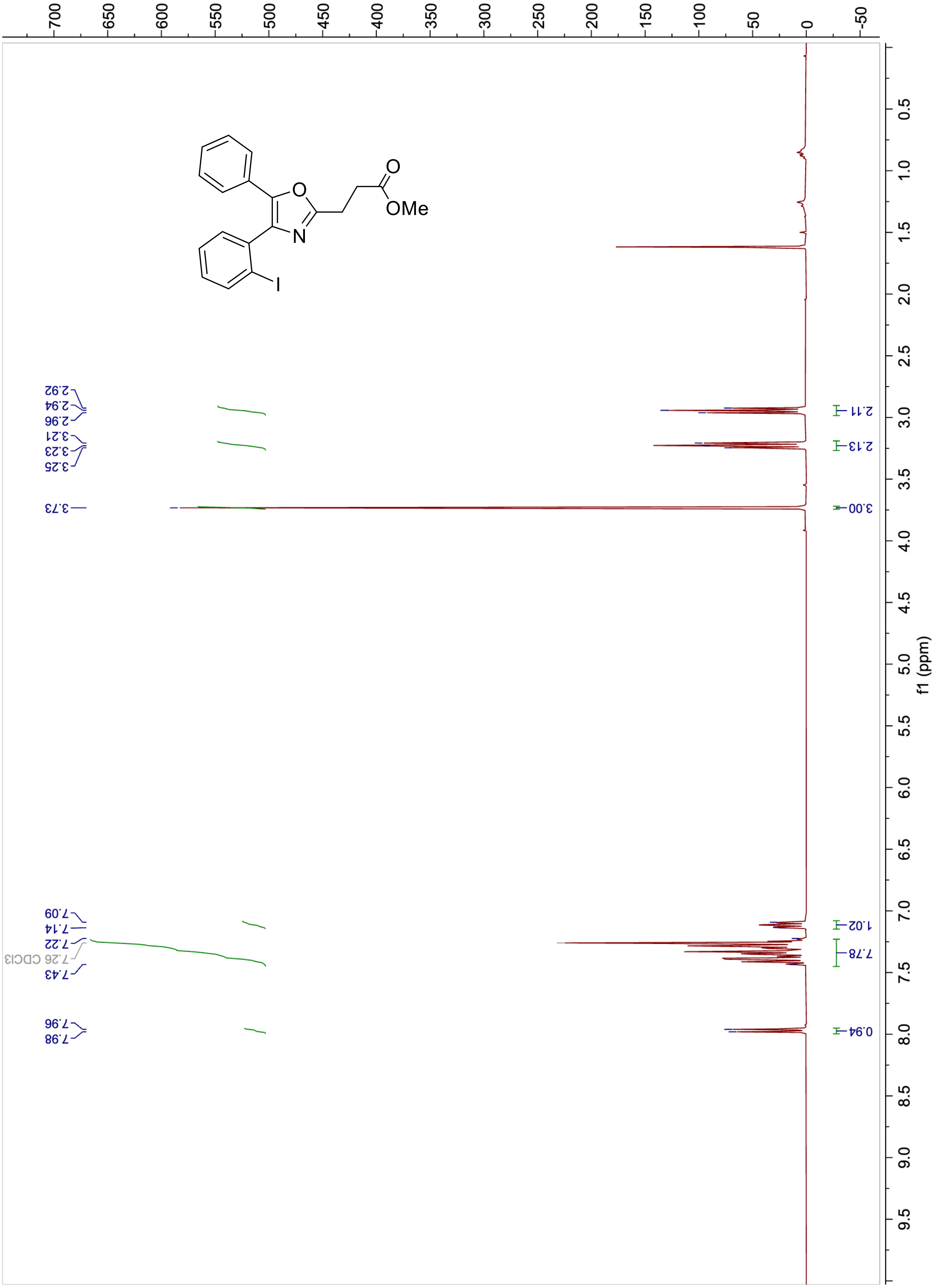

$^{13}C\{^1H\}$ NMR 101 MHz **4b**

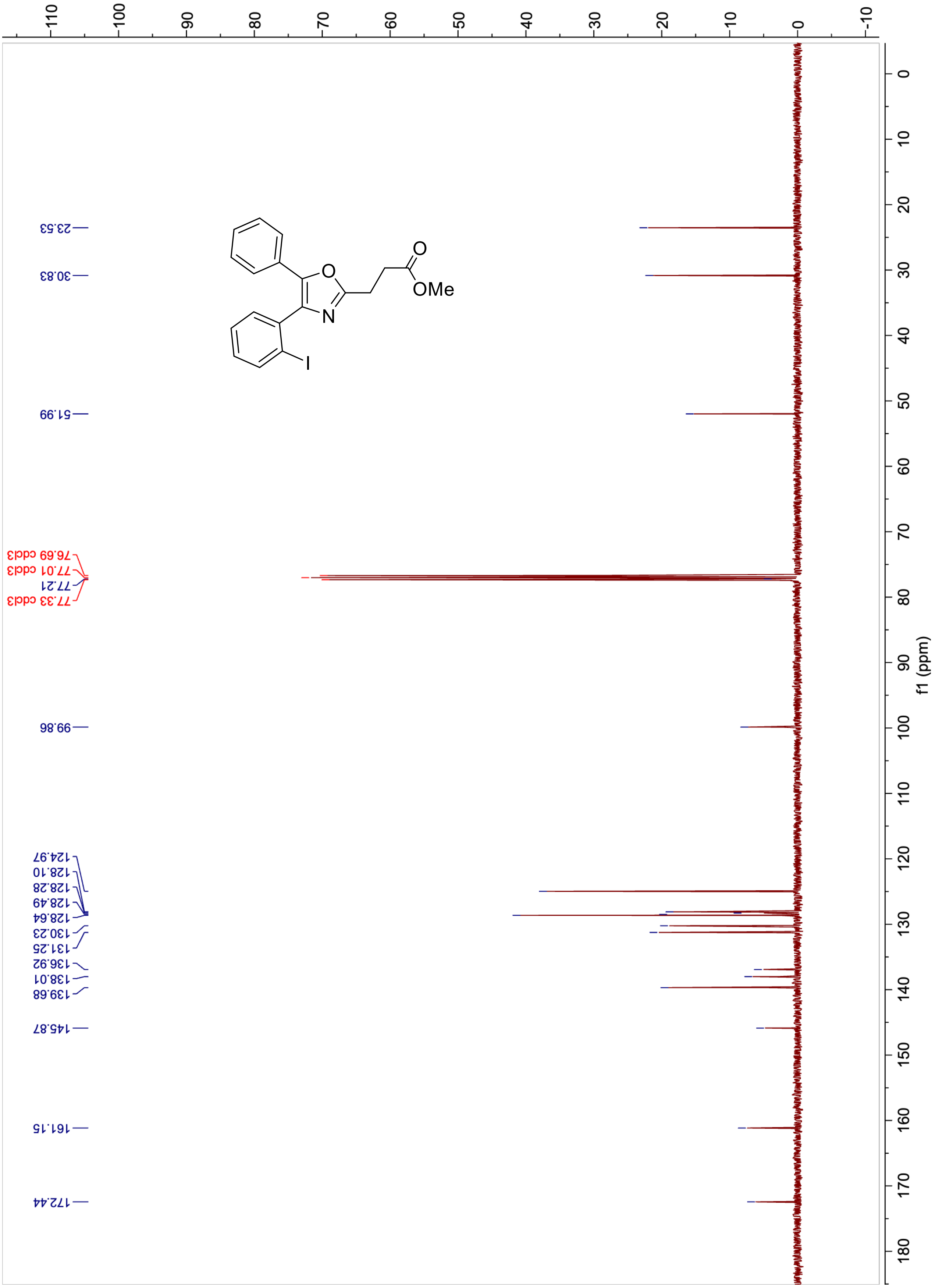

^{1}H NMR 600 MHz **4c**

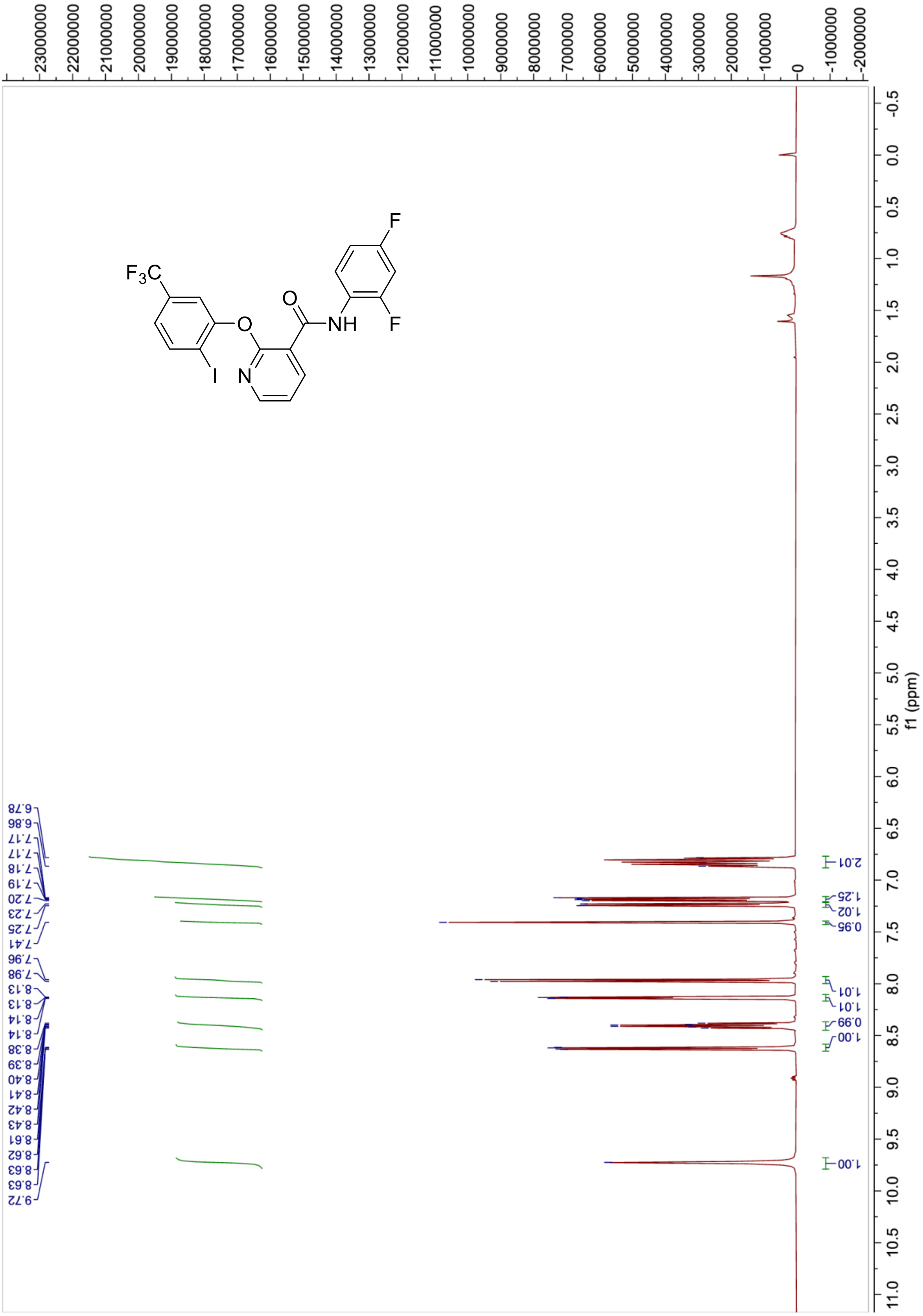

$^{13}C\{^{1}H\}$ NMR 126 MHz **4c**

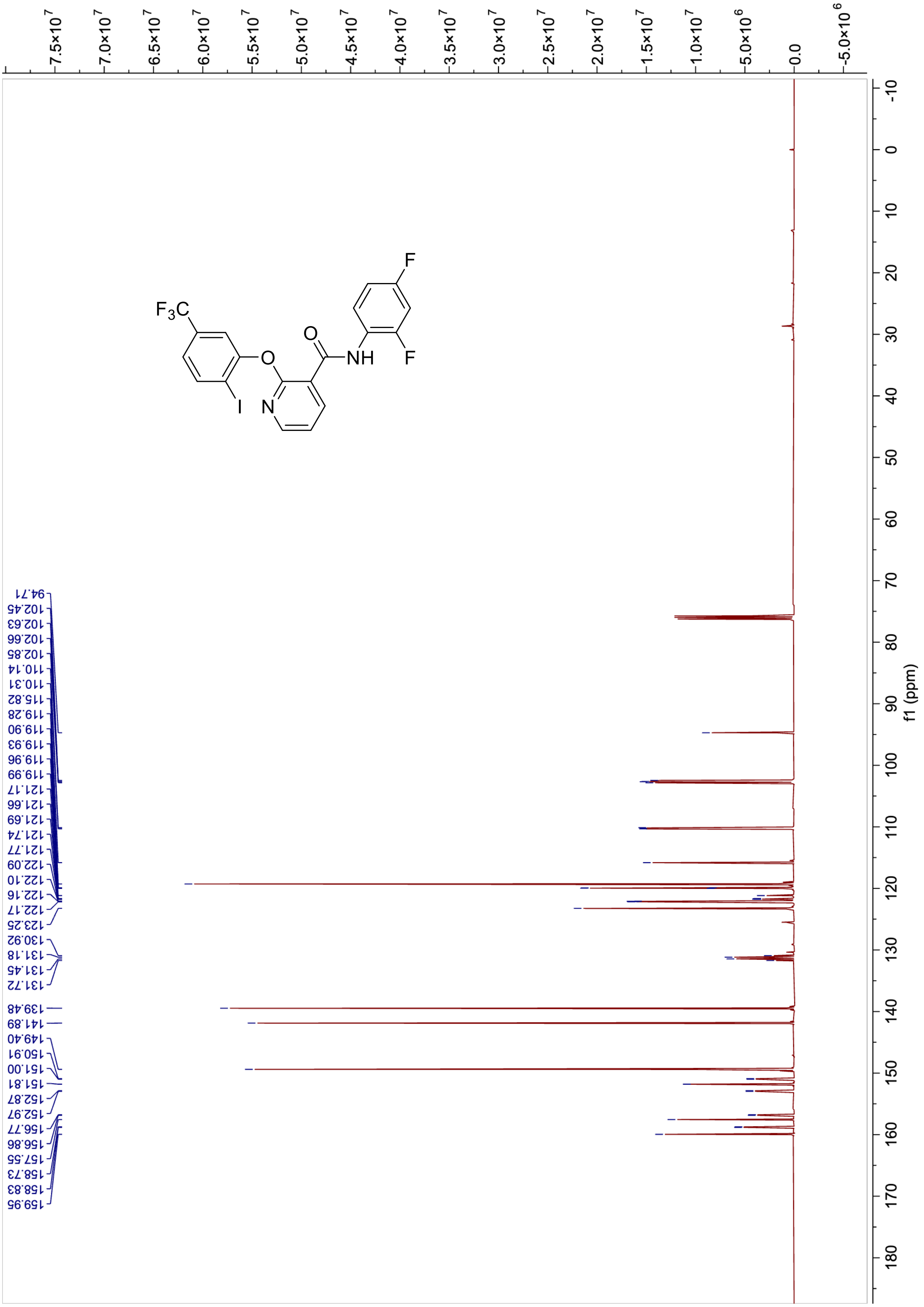

^{19}F NMR 376 MHz **4c**

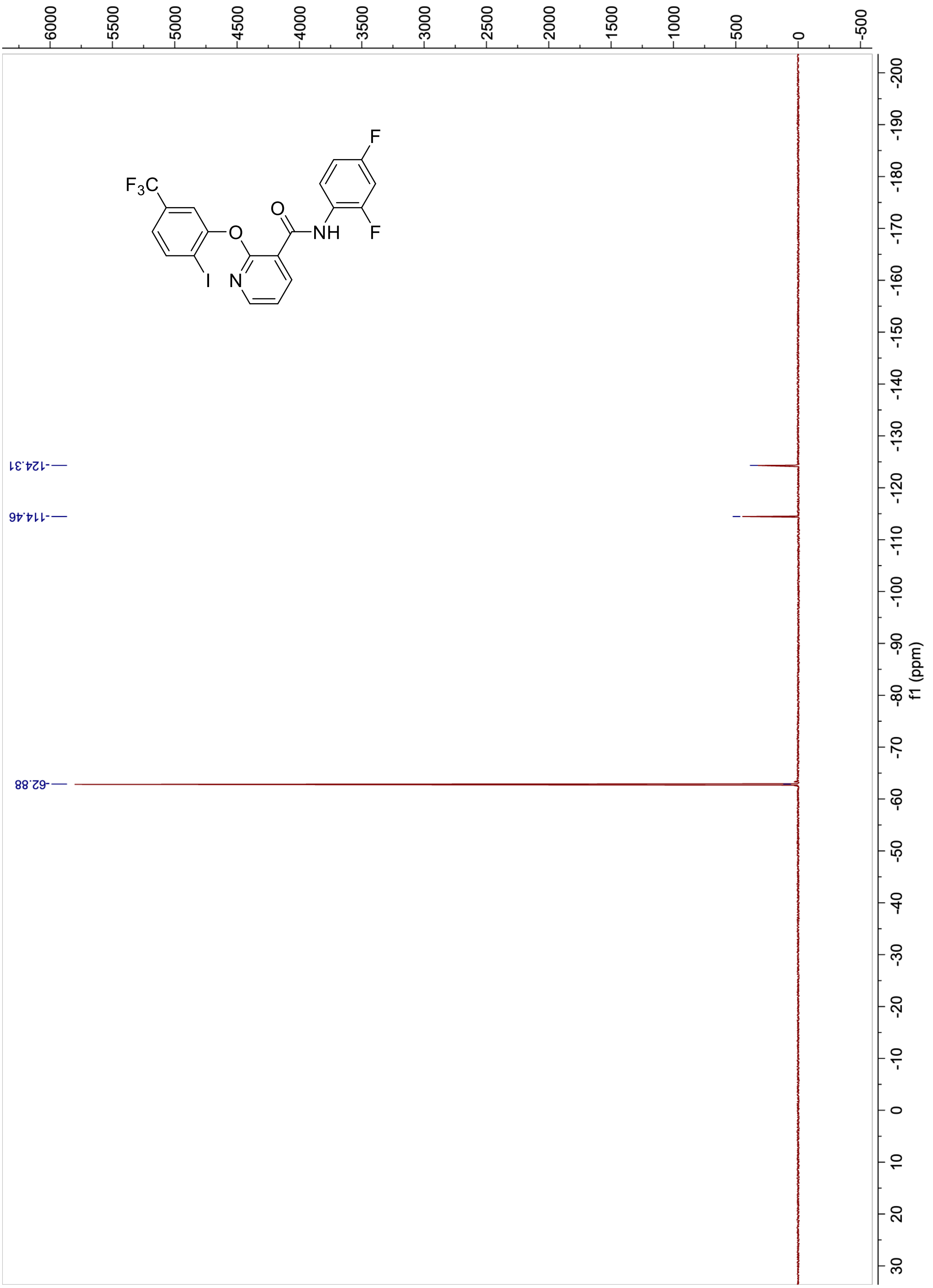

^{1}H NMR 400 MHz **6c**

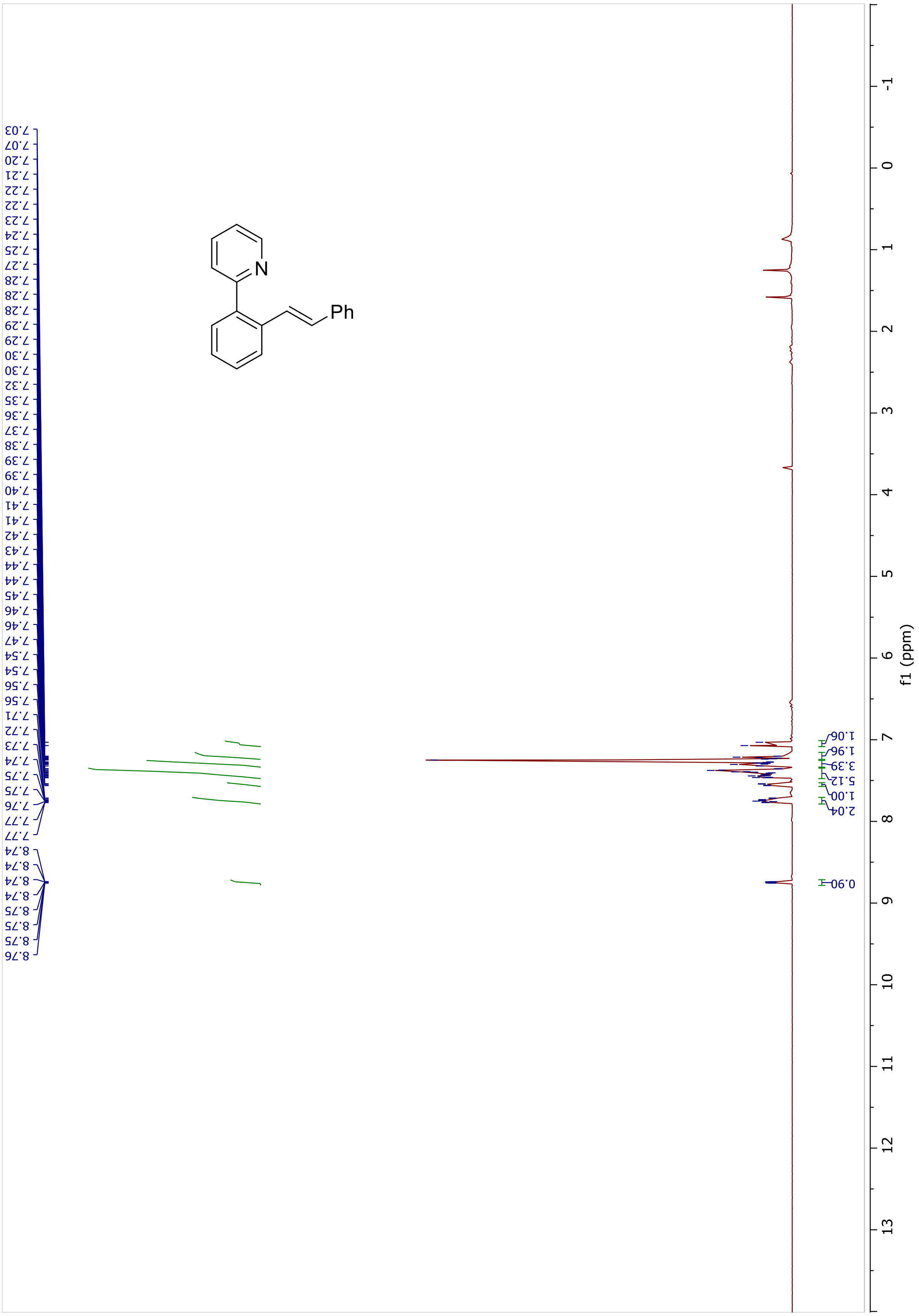

^{1}H NMR 500 MHz **6d**

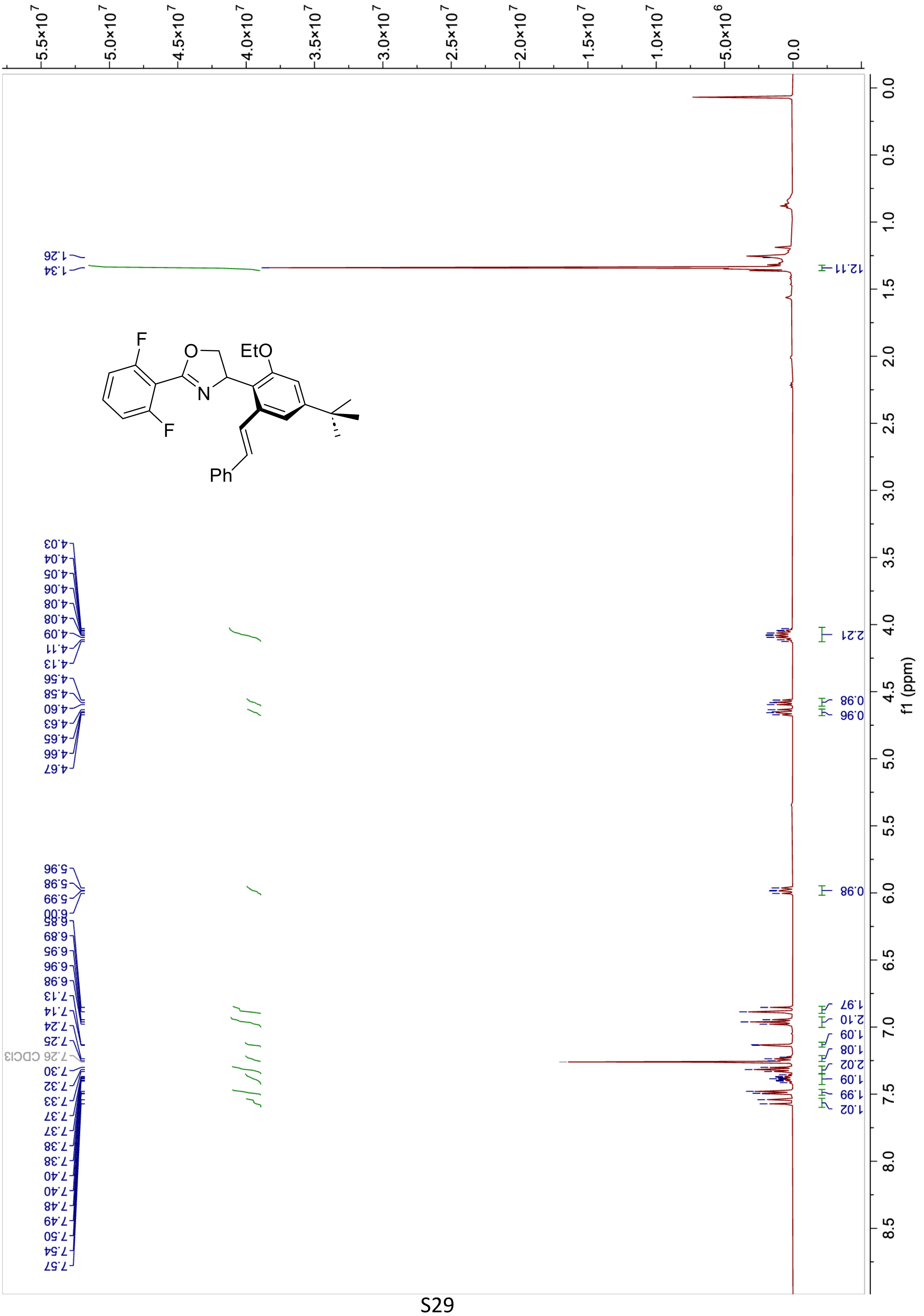

$^{13}C\{^{1}H\}$ NMR 126 MHz **6d**

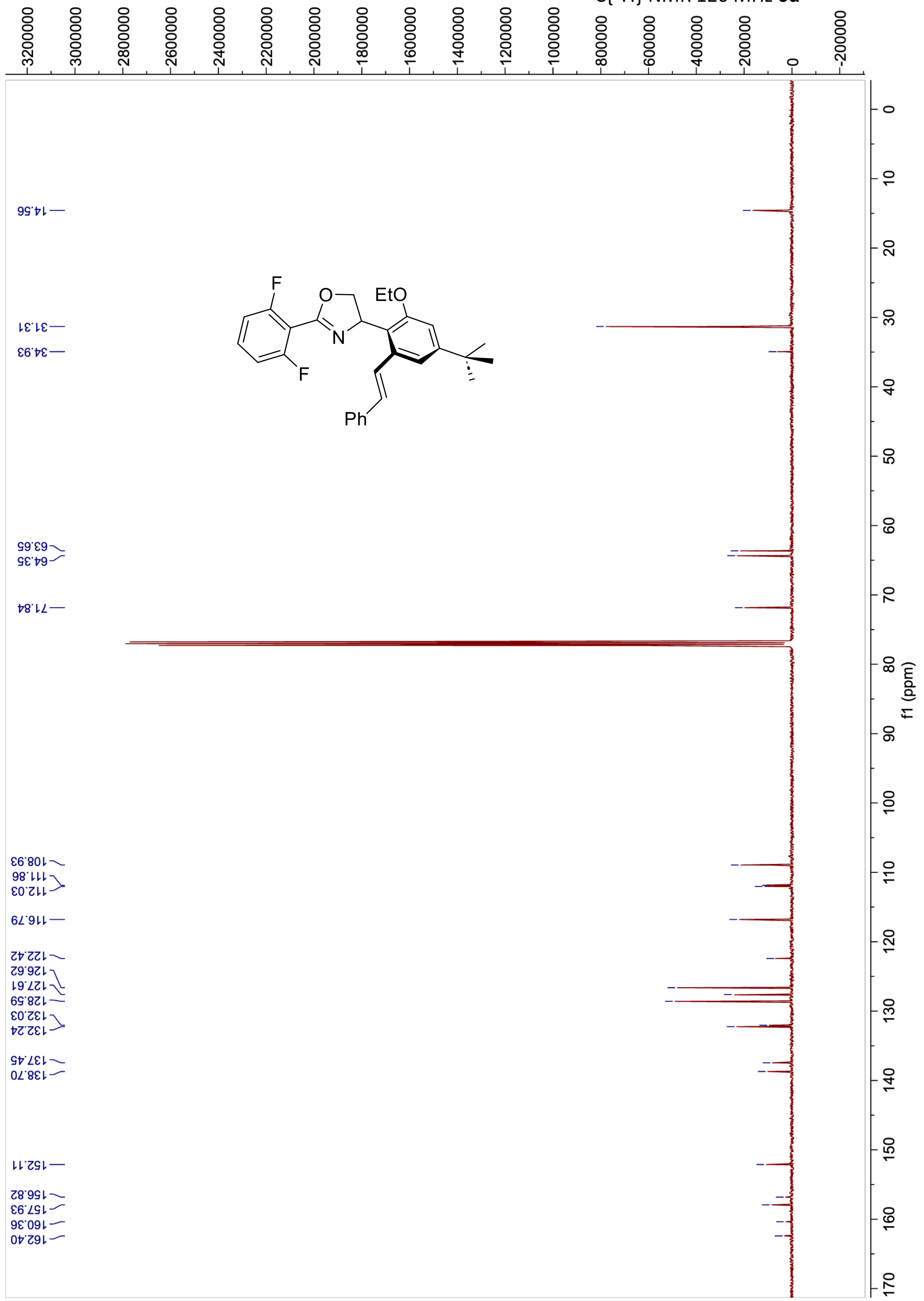

^{19}F NMR 376 MHz **6d**

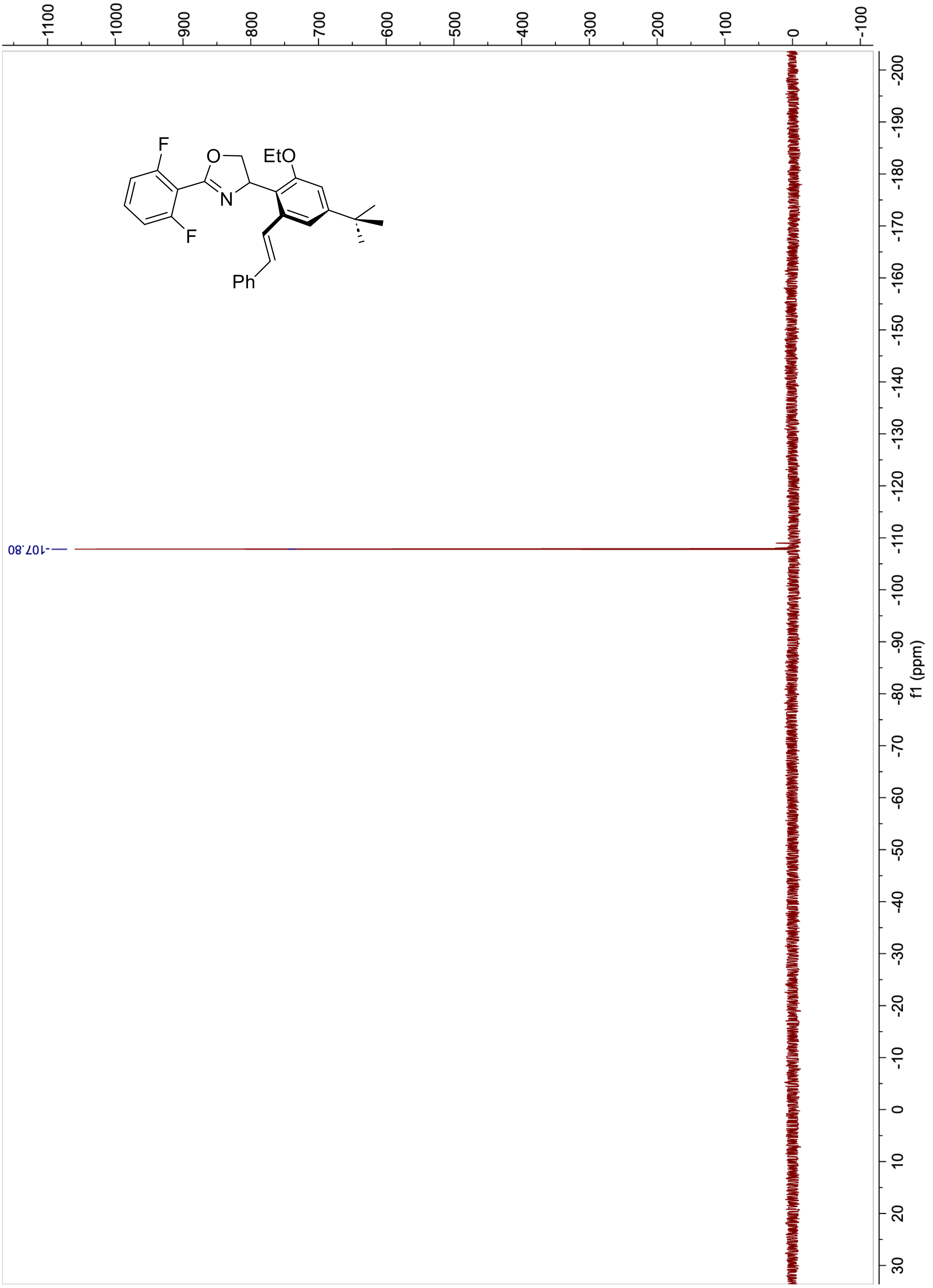

^{1}H NMR 400 MHz **7a**

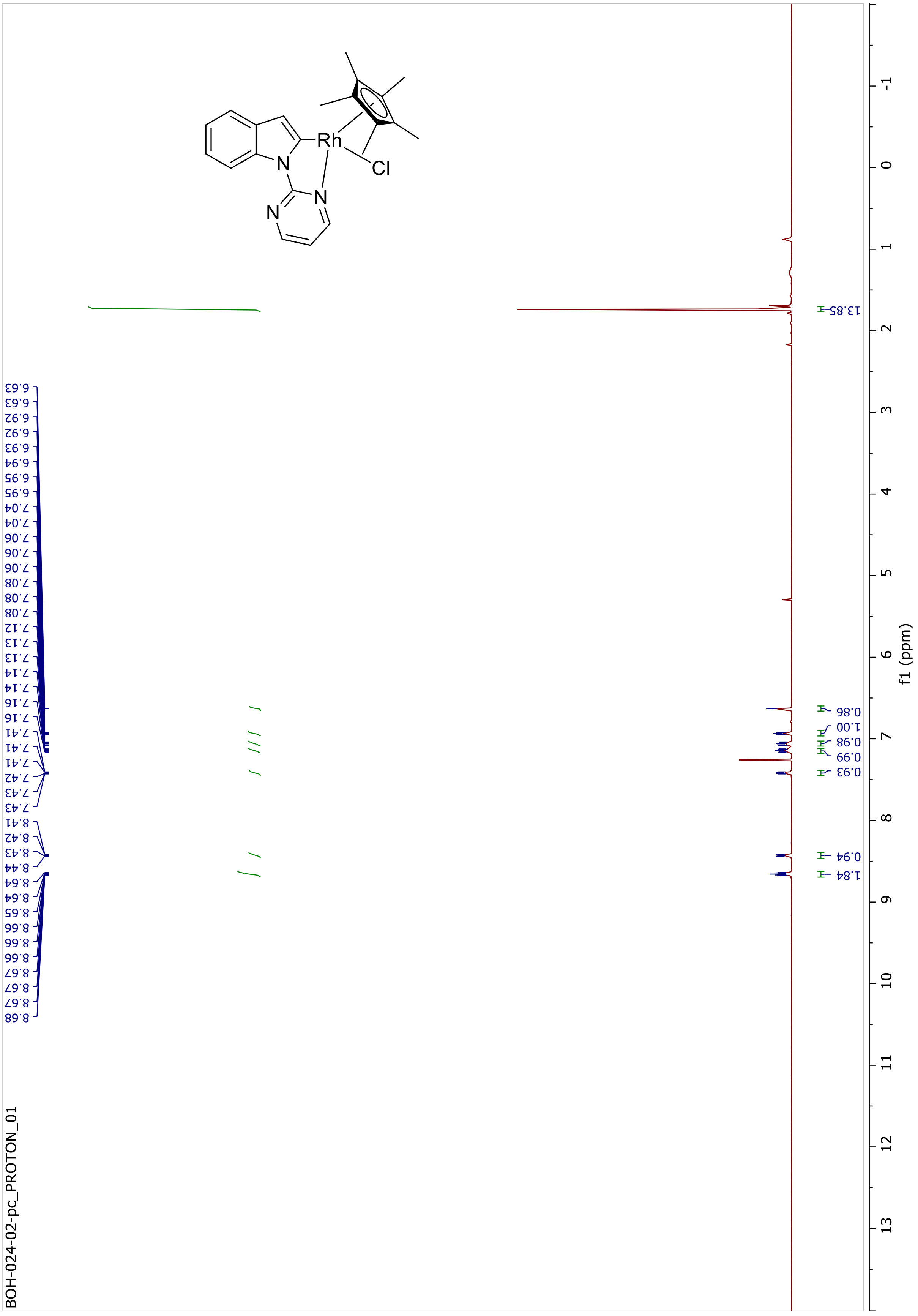

^{1}H NMR 400 MHz **7b**

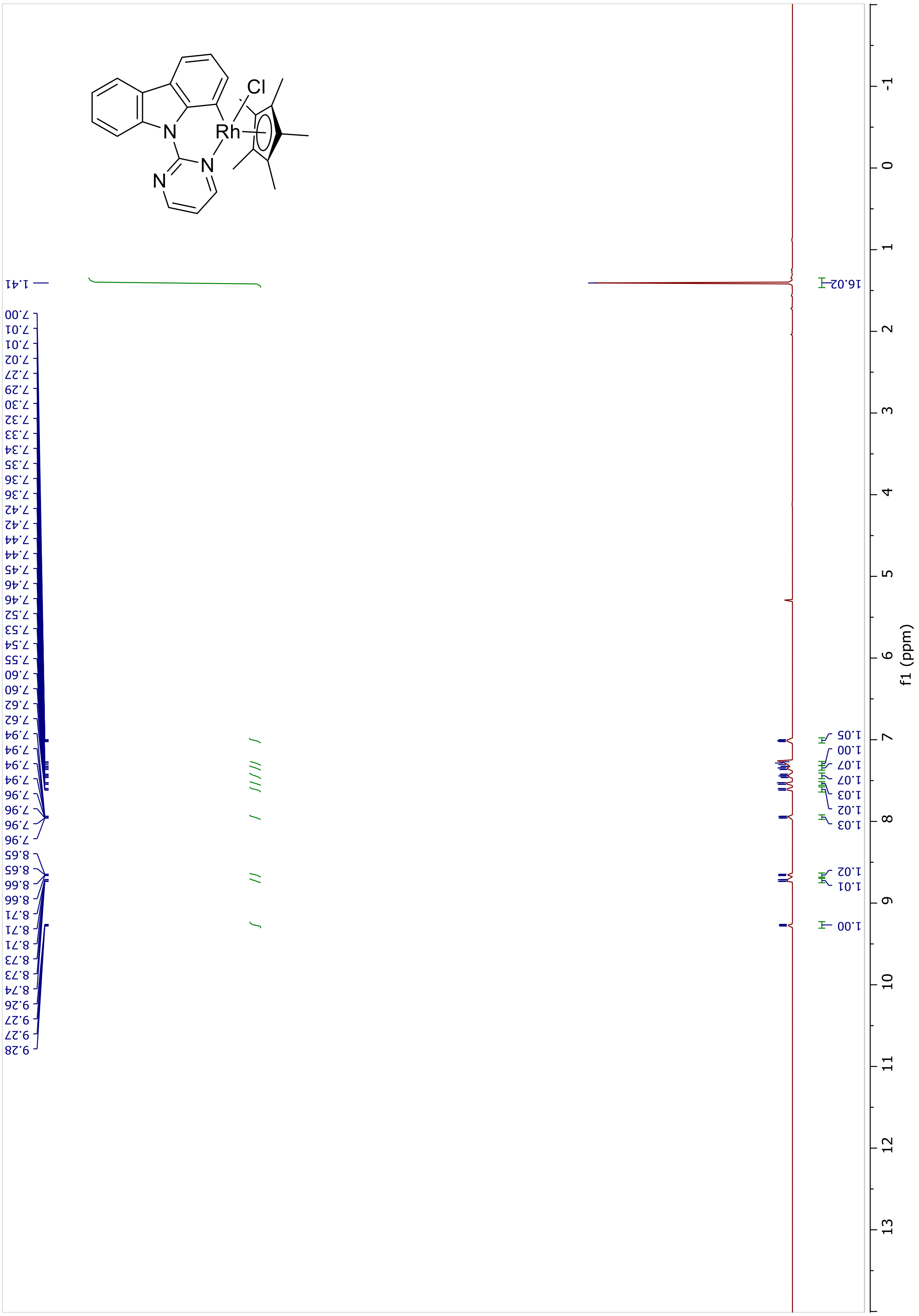

^{1}H NMR 400 MHz **7c**

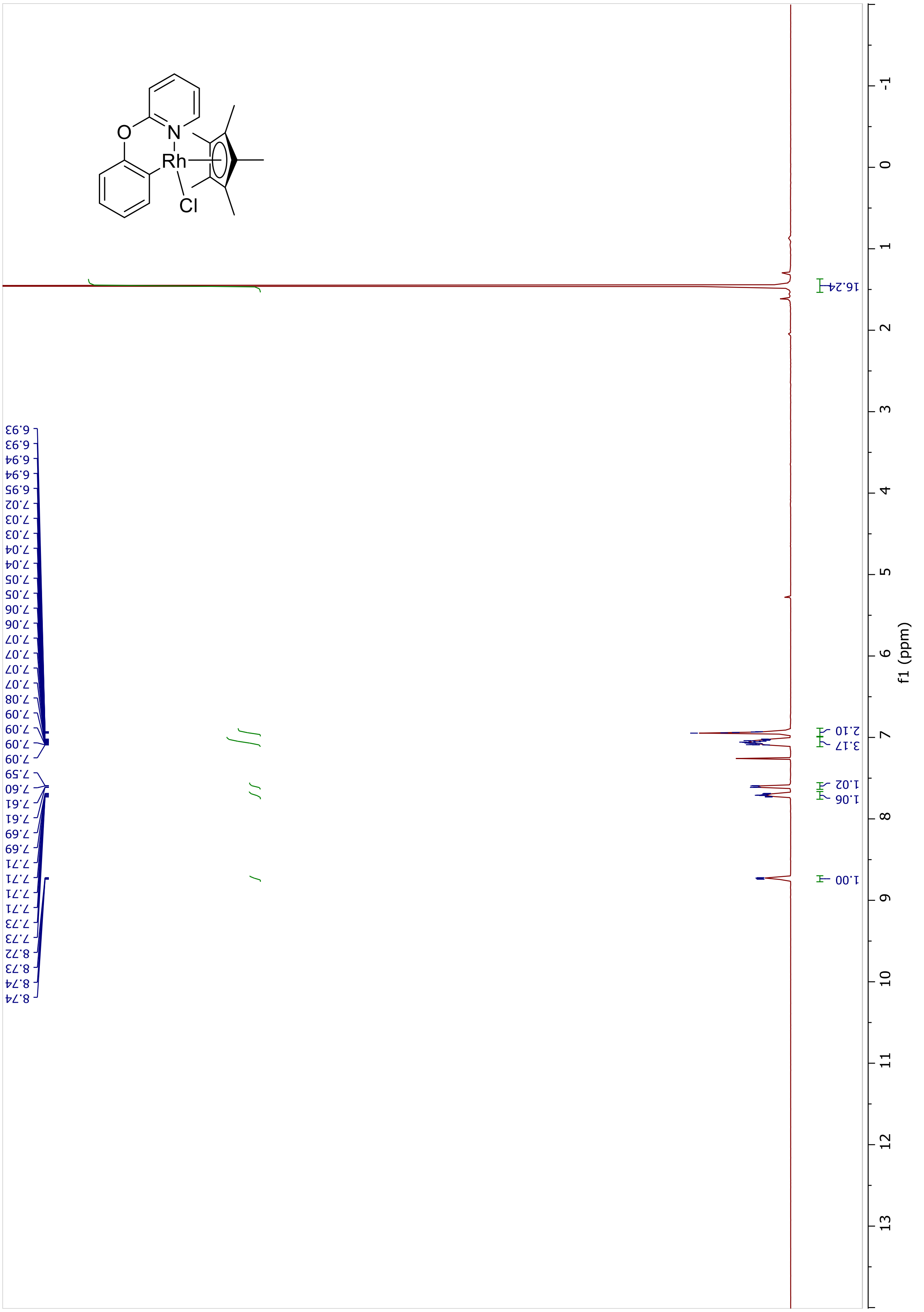

^{1}H NMR 400 MHz **8a**

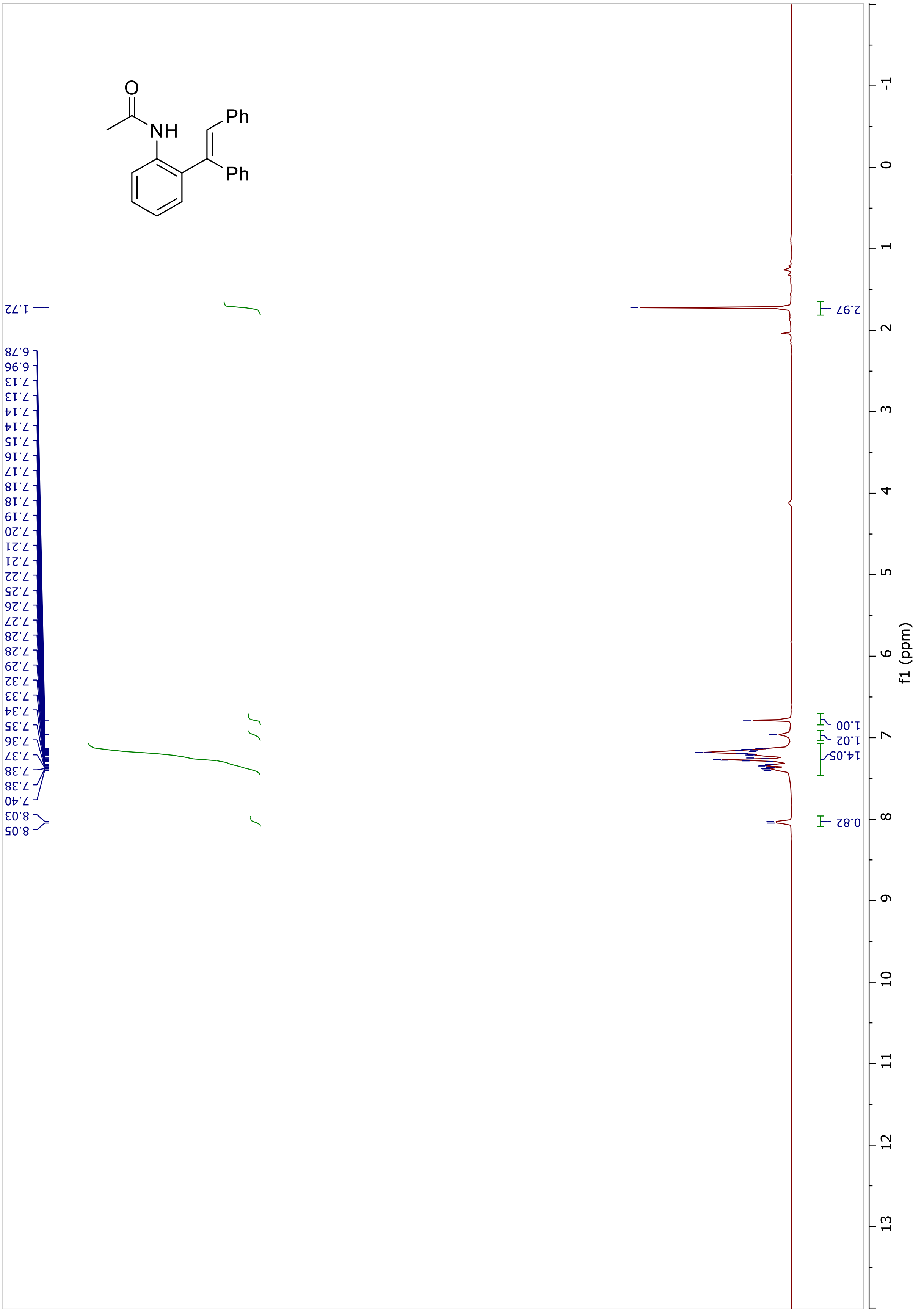

^{1}H NMR 400 MHz **8c**

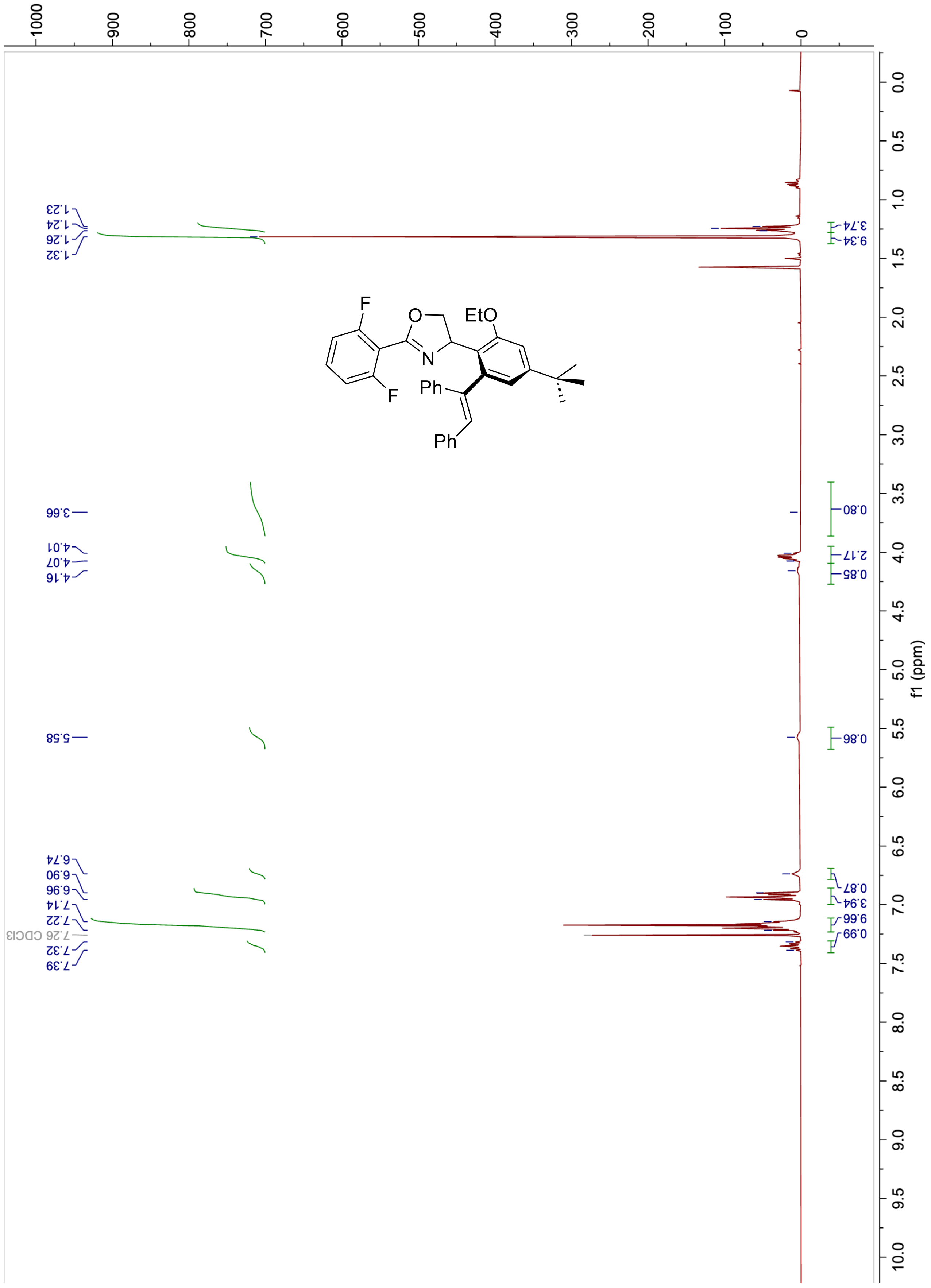

13C{1H} NMR 101 MHz **8c**

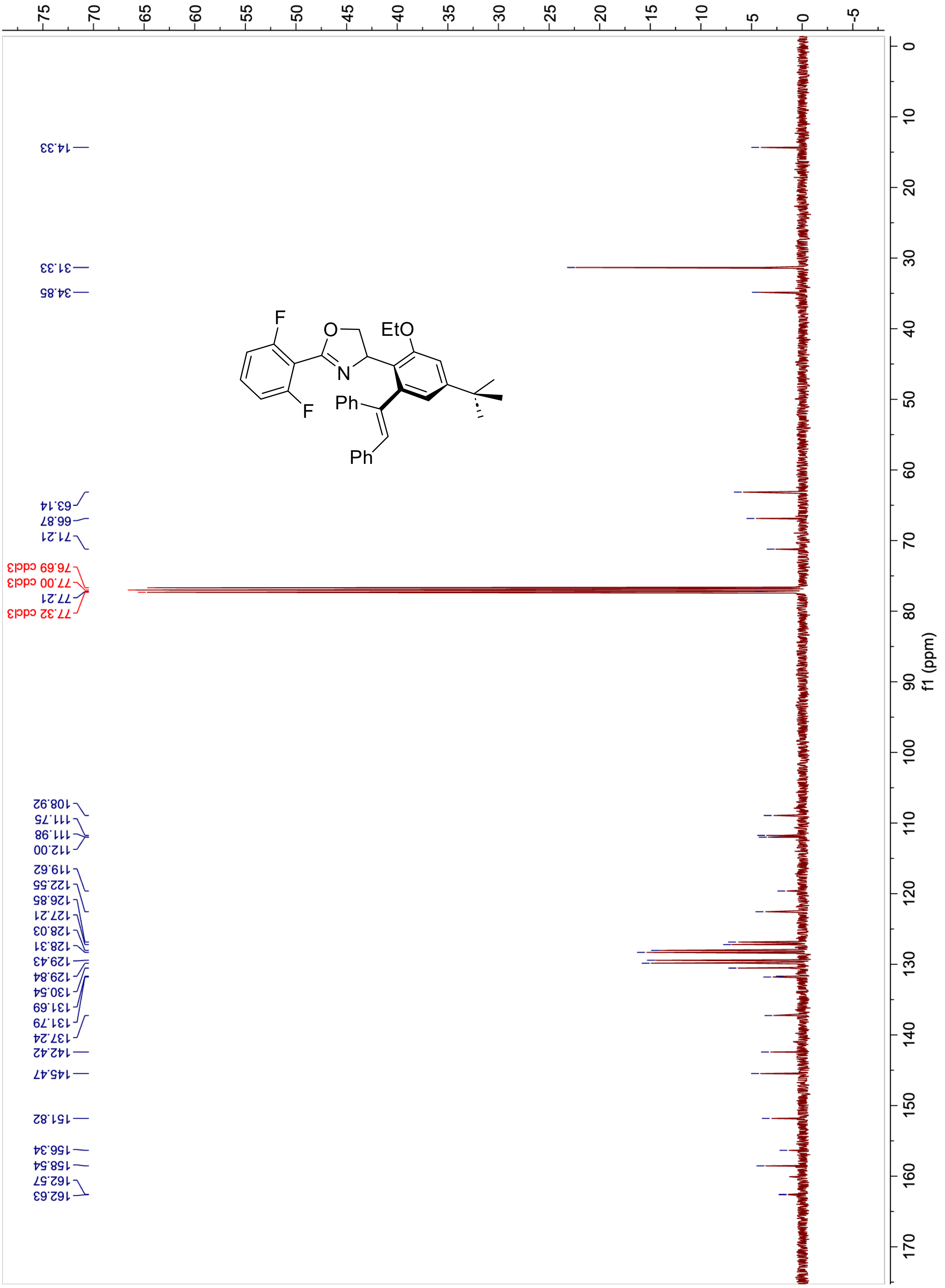

^{19}F NMR 376 MHz **8c**

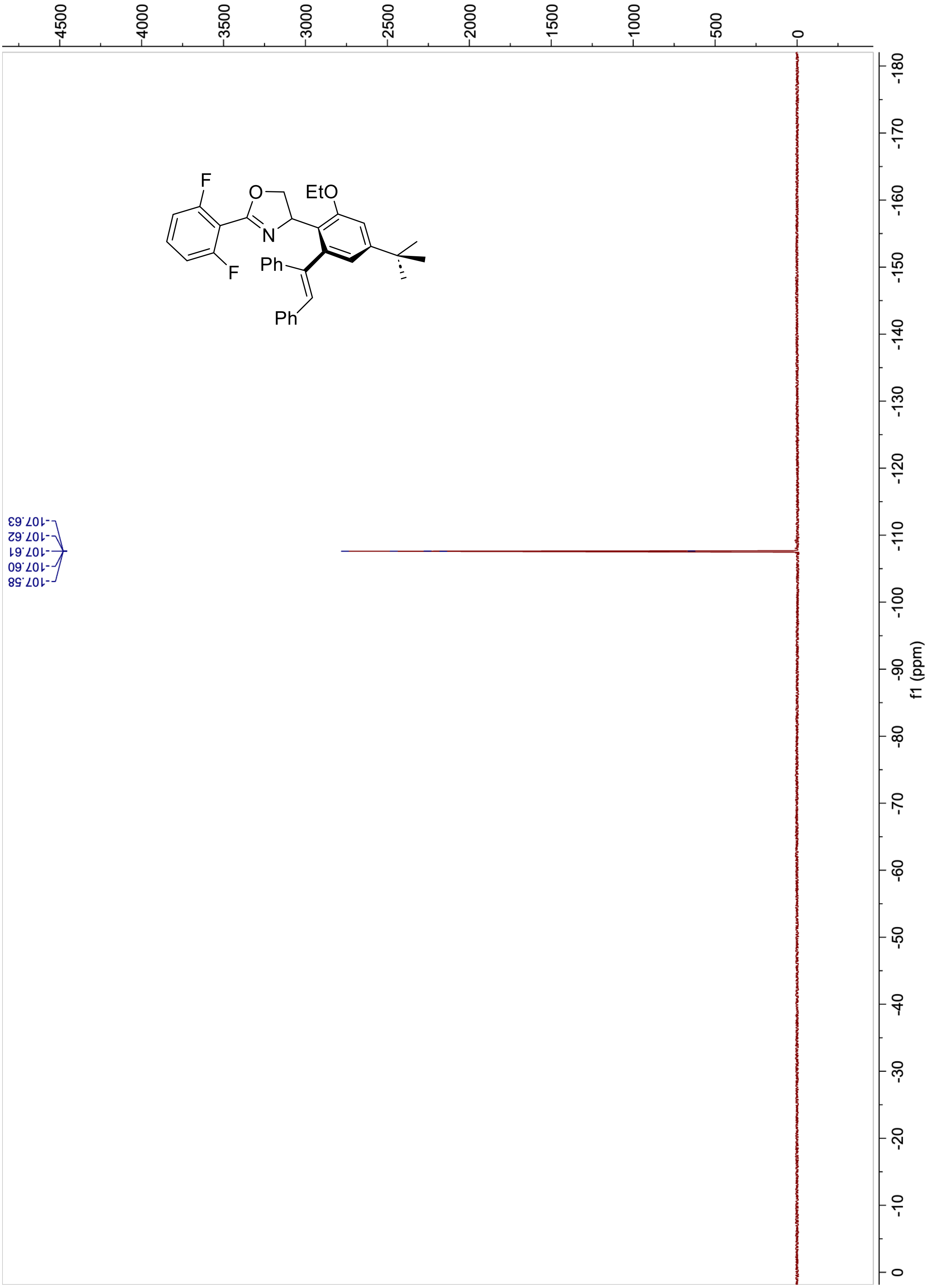

^{1}H NMR 400 MHz **9**

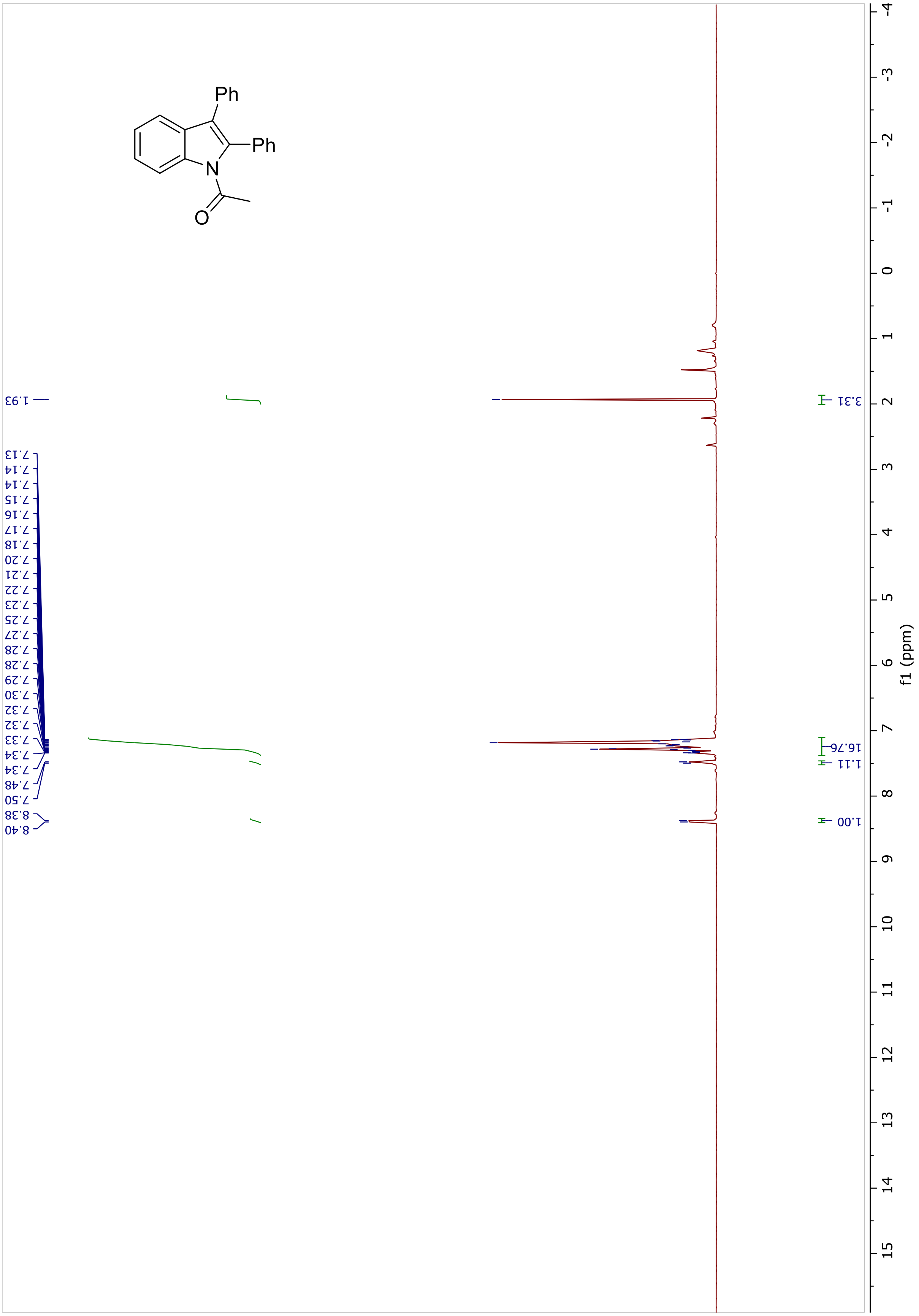

^{1}H NMR 400 MHz **11b**

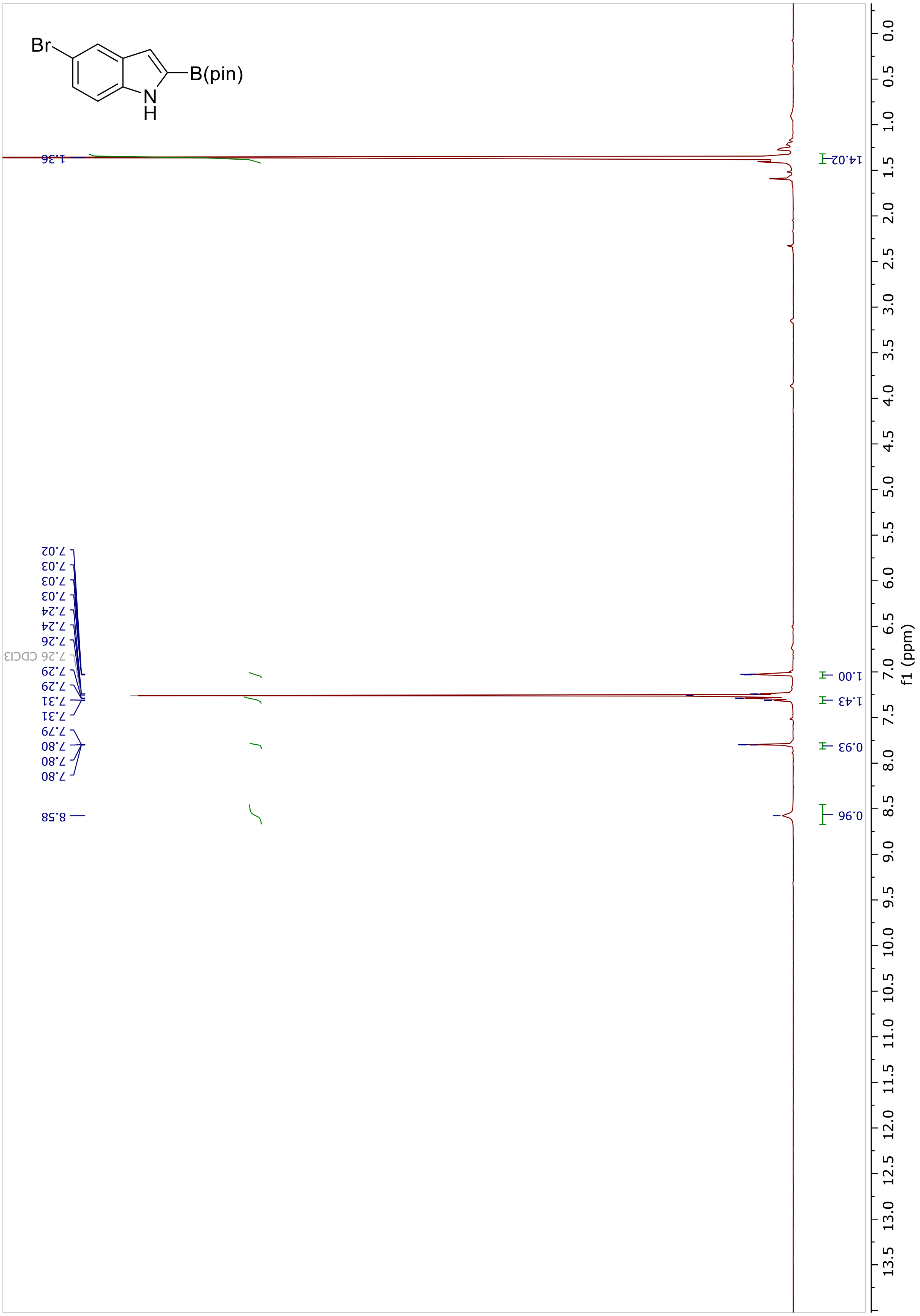

^{1}H NMR 400 MHz **11c**

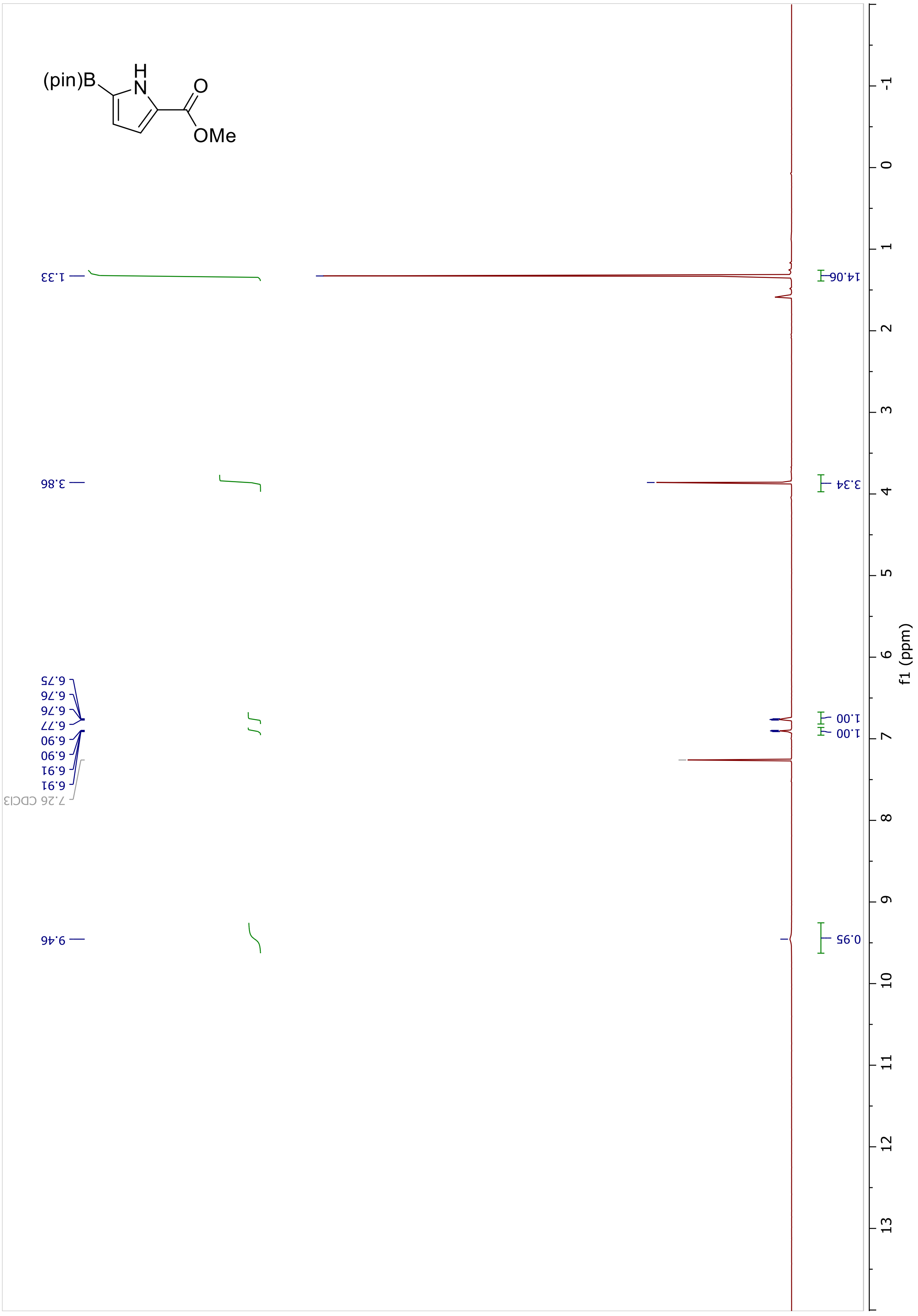

^{1}H NMR 400 MHz **11d**

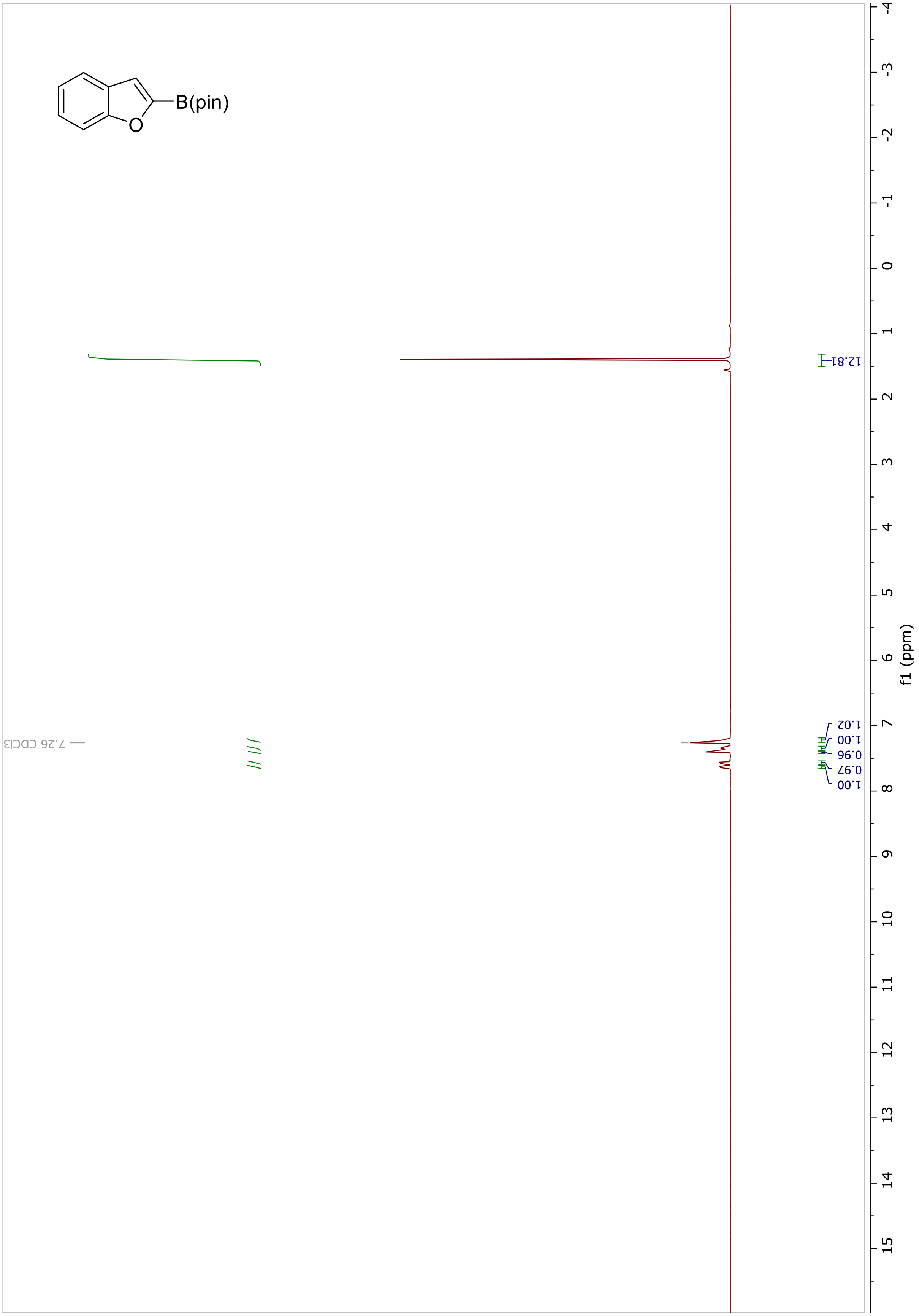

^{1}H NMR 400 MHz **11e**

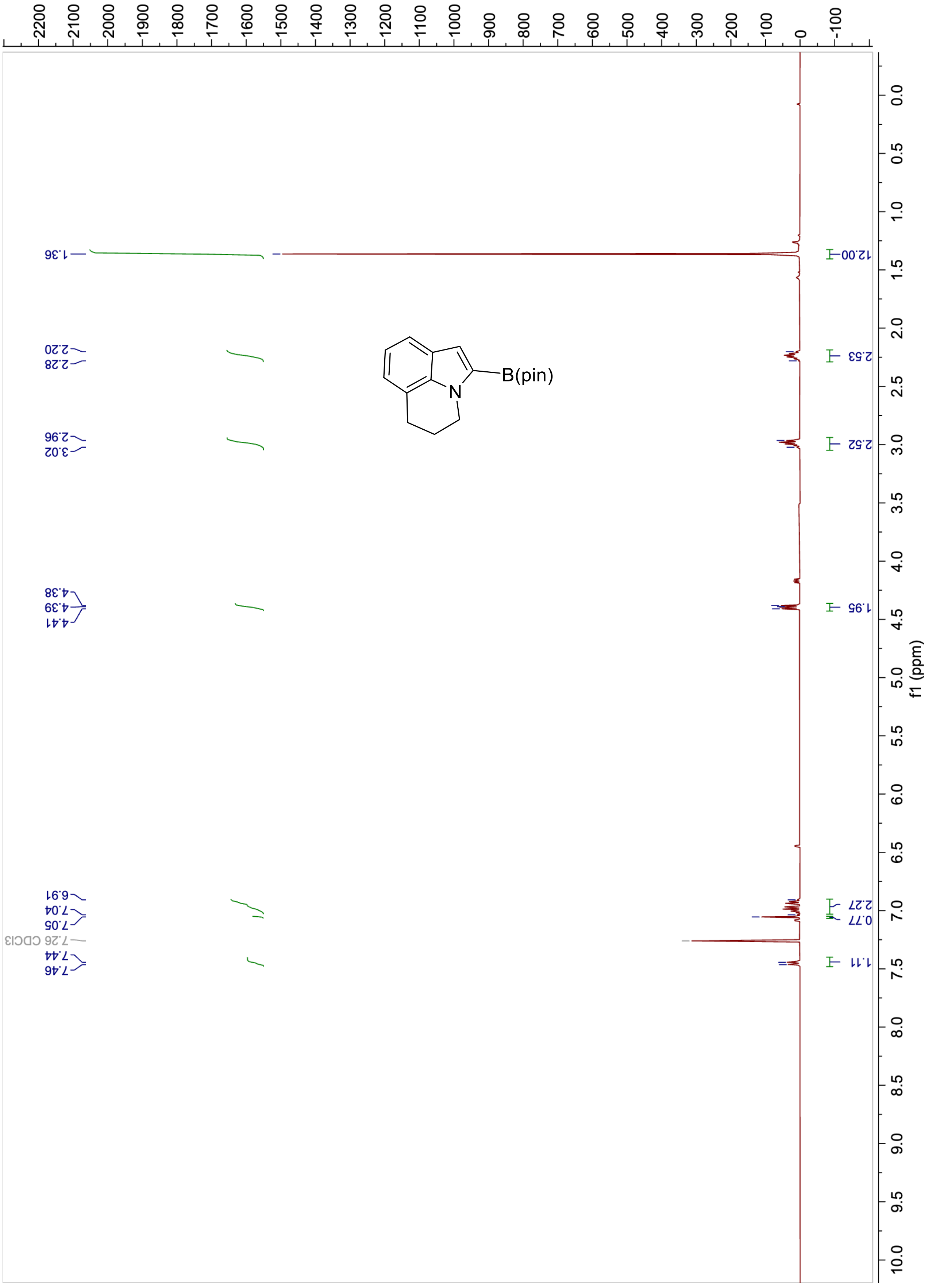

$^{13}C\{^{1}H\}$ NMR 101 MHz **11e**

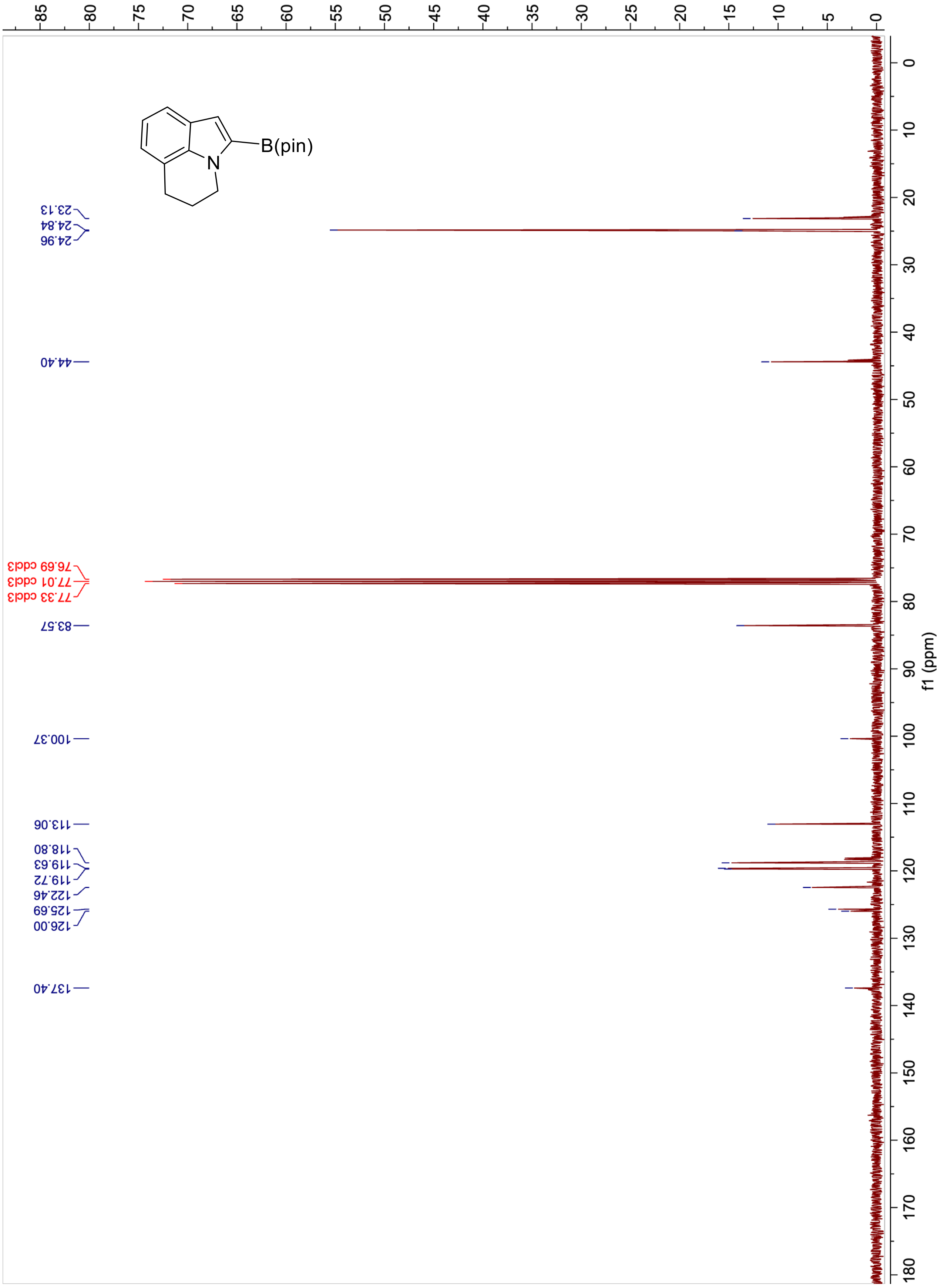

^{1}H NMR 400 MHz **13a**

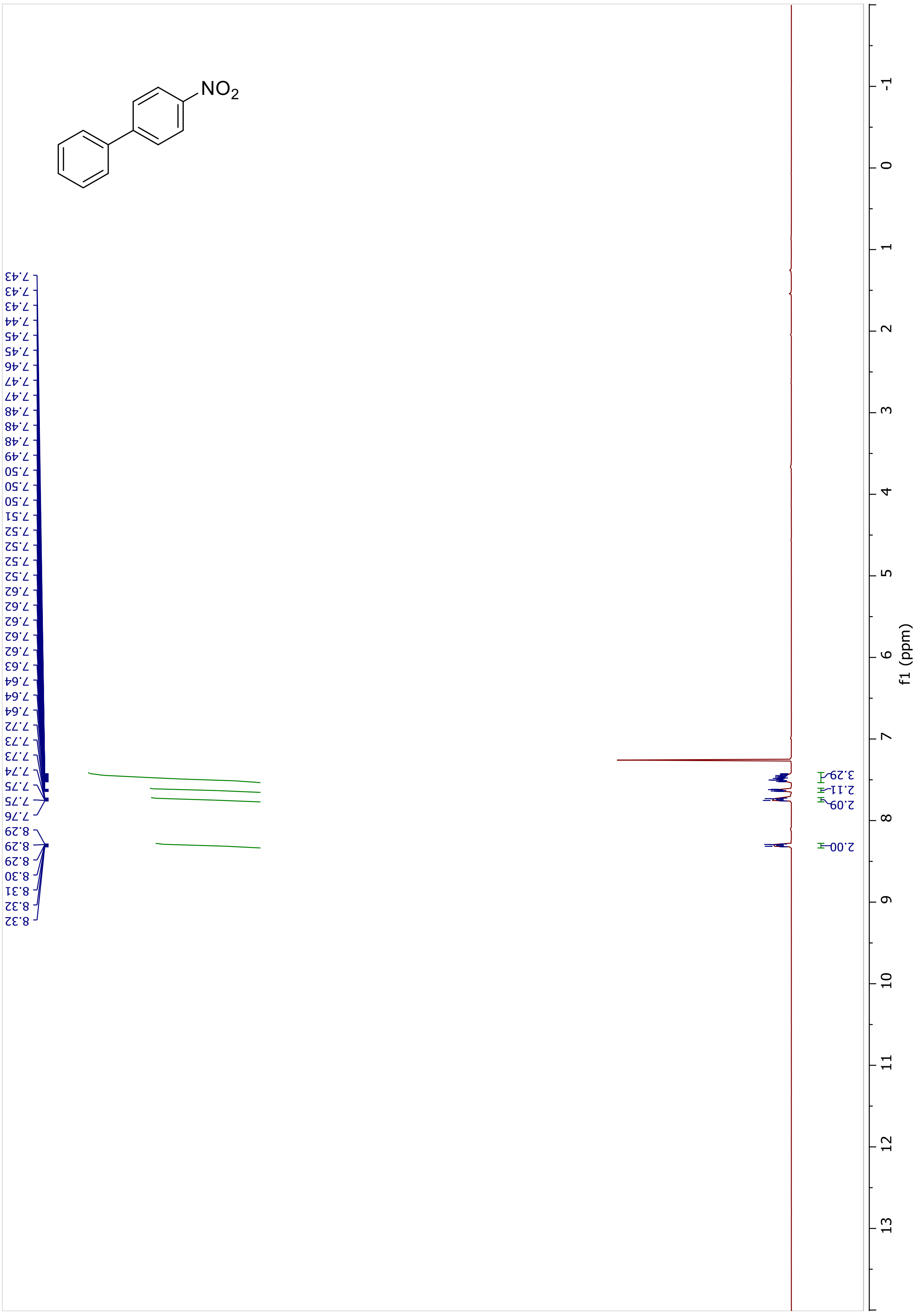

^{1}H NMR 400 MHz **13d**

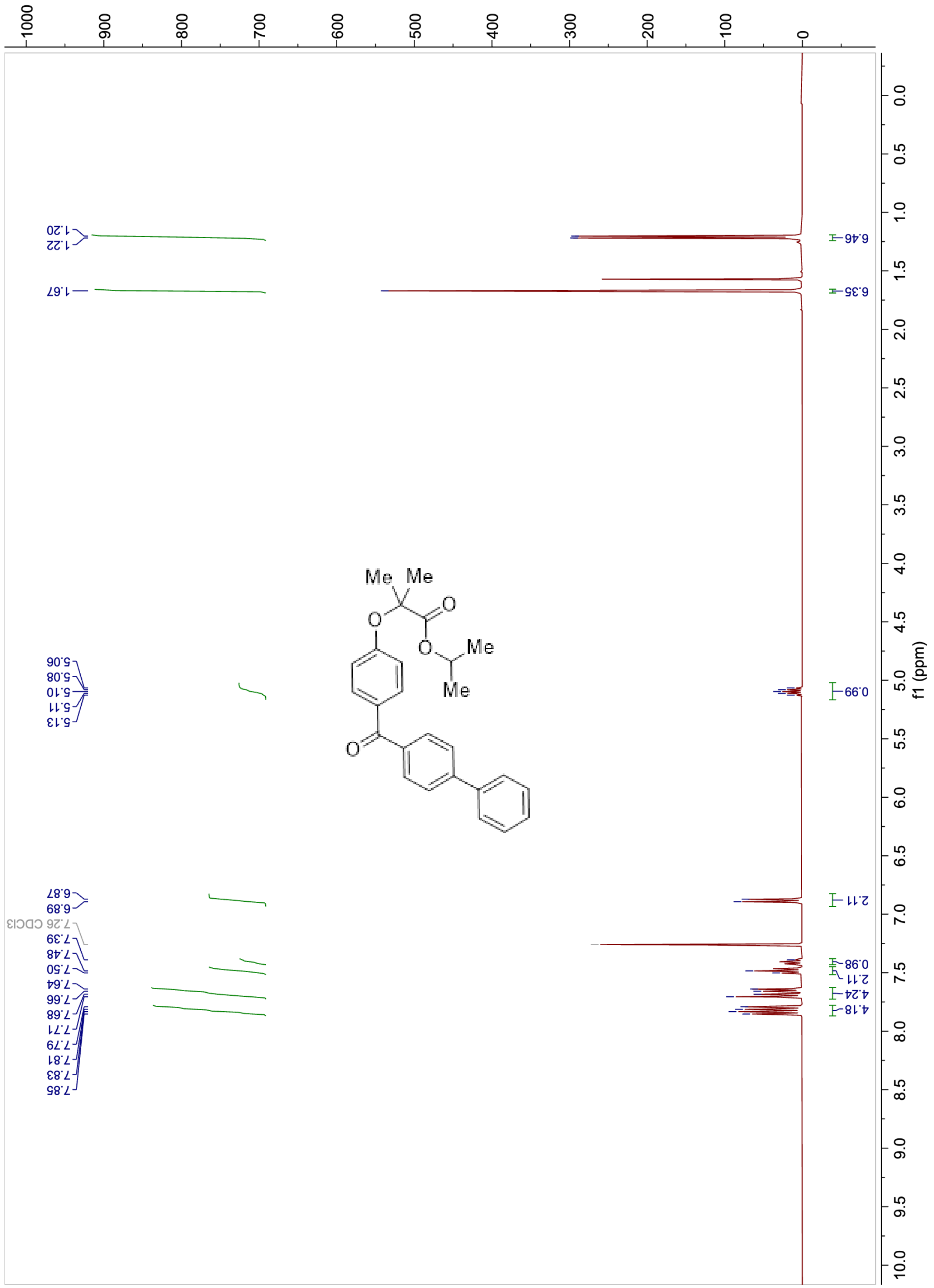

$^{13}C\{^{1}H\}$ NMR 101 MHz **13d**

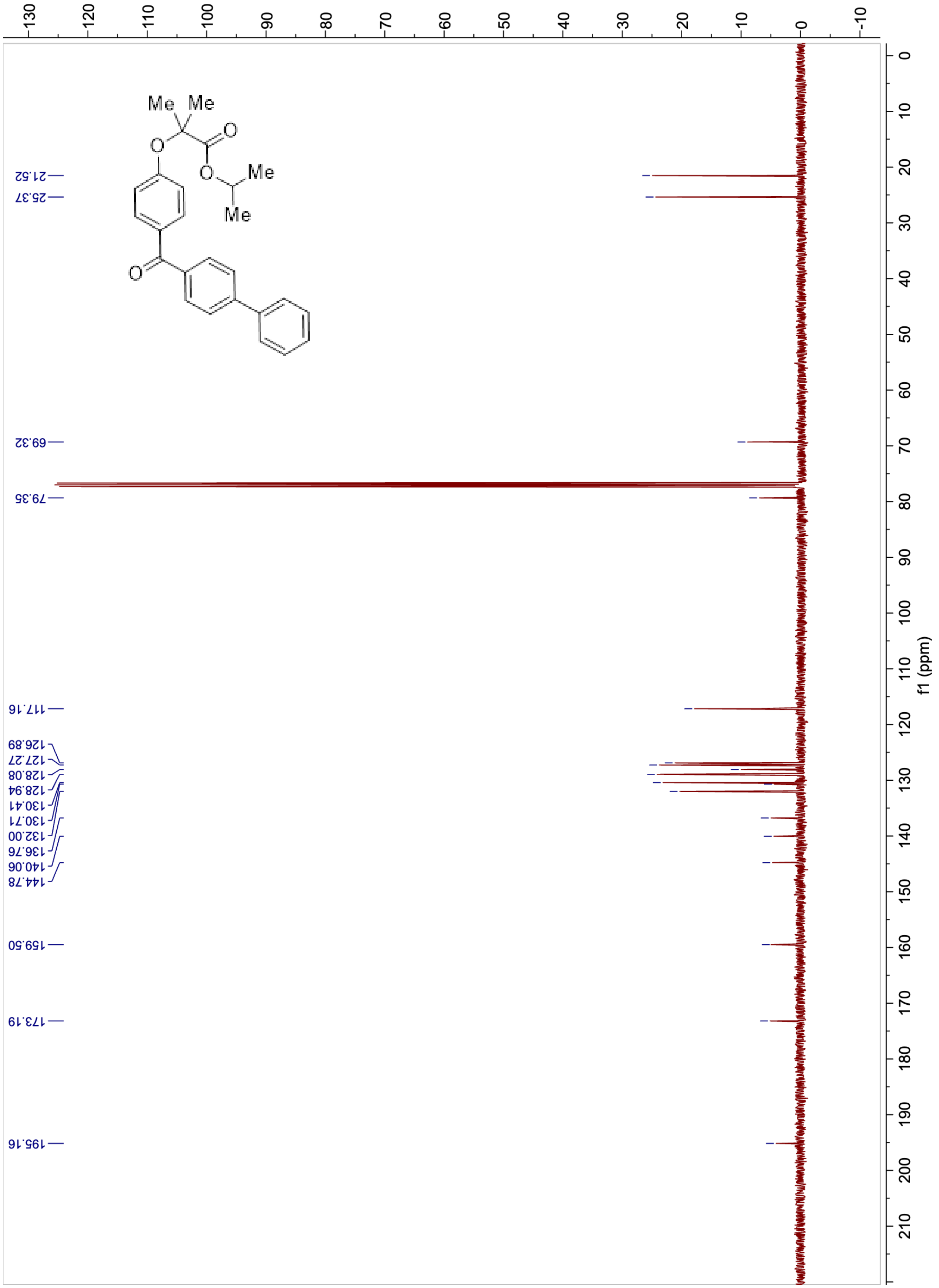

^{1}H NMR 400 MHz **13e**

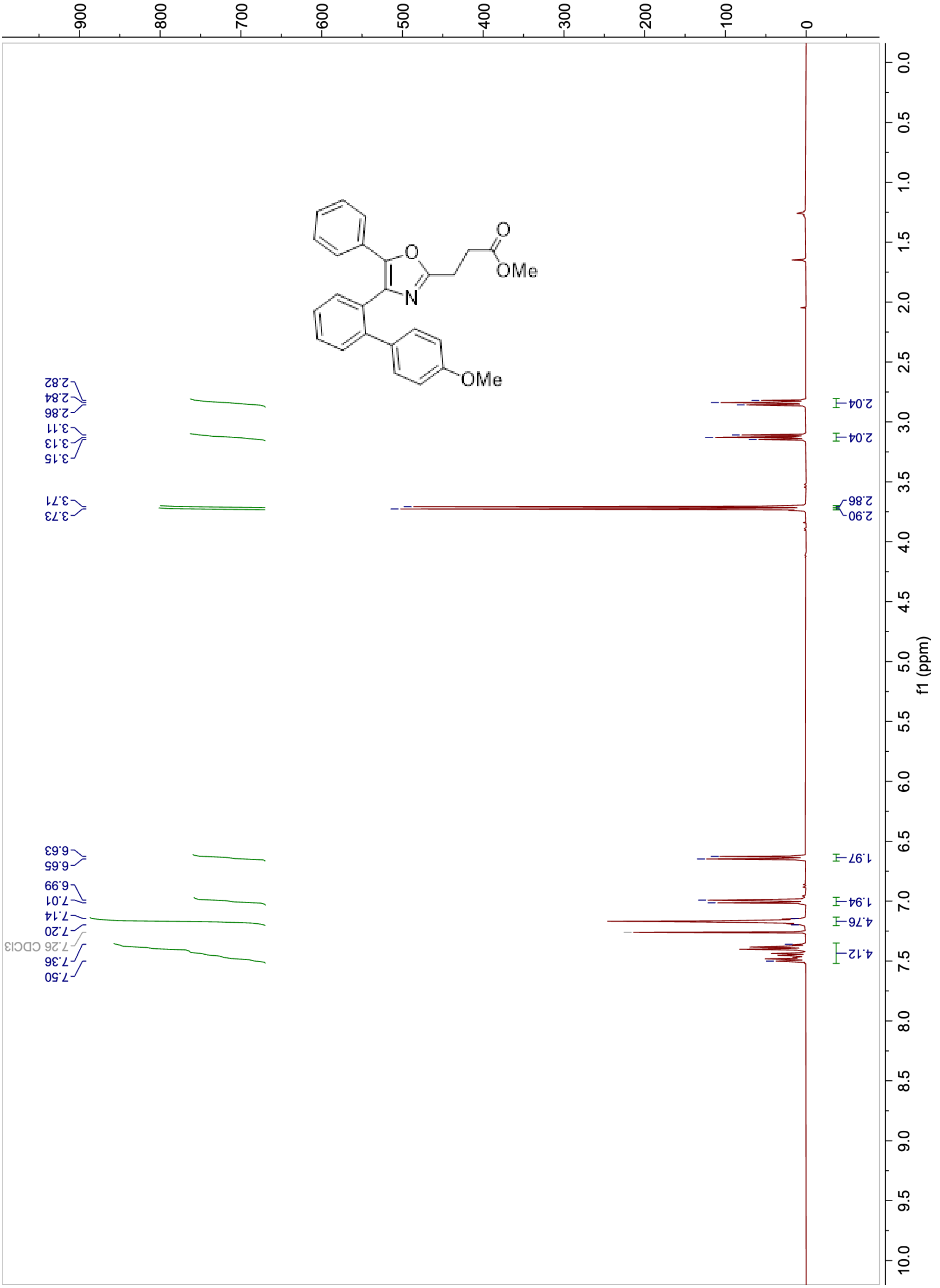

$^{13}C\{^{1}H\}$ NMR 101 MHz **13e**

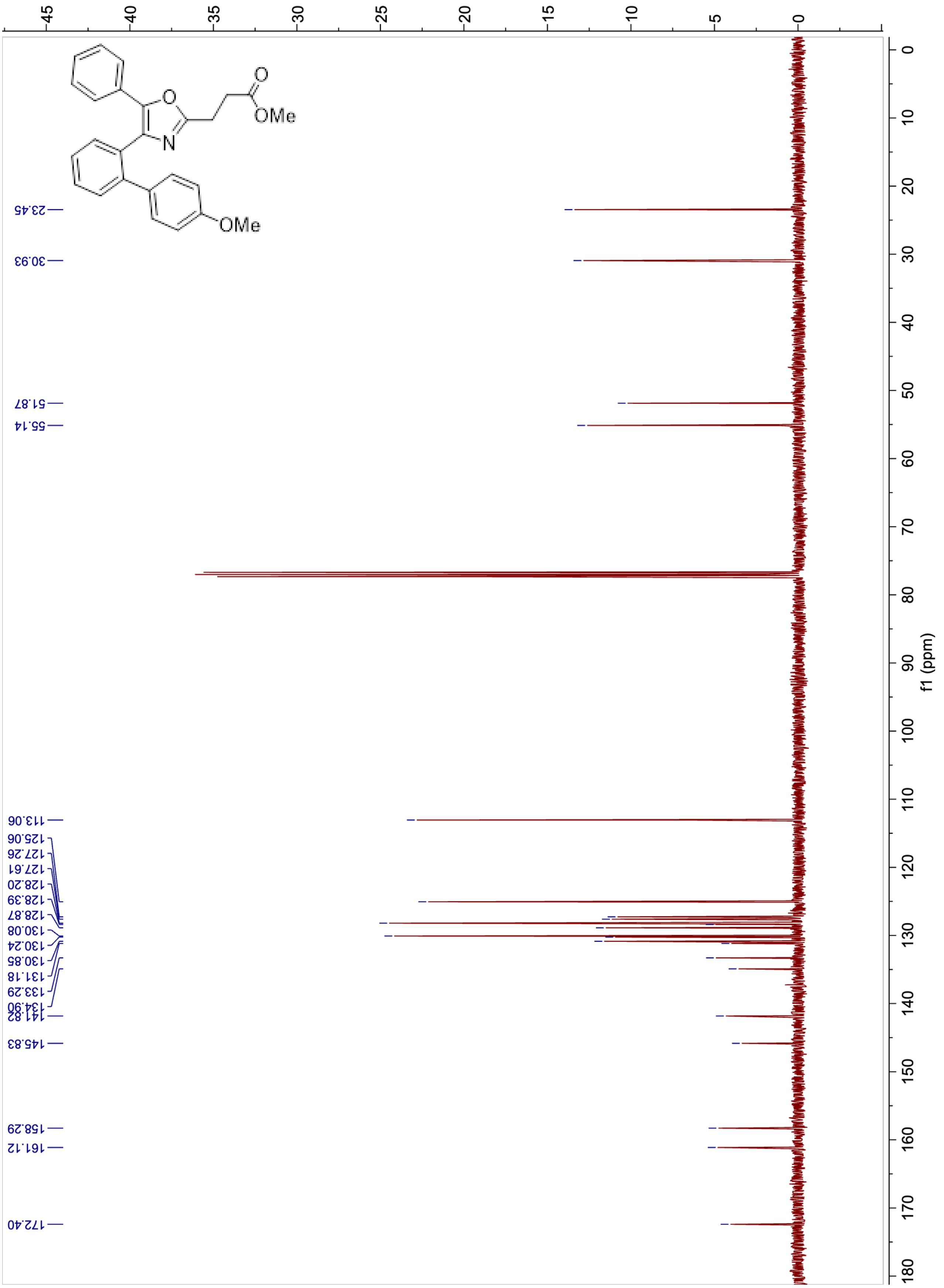

^{1}H NMR 400 MHz **14**

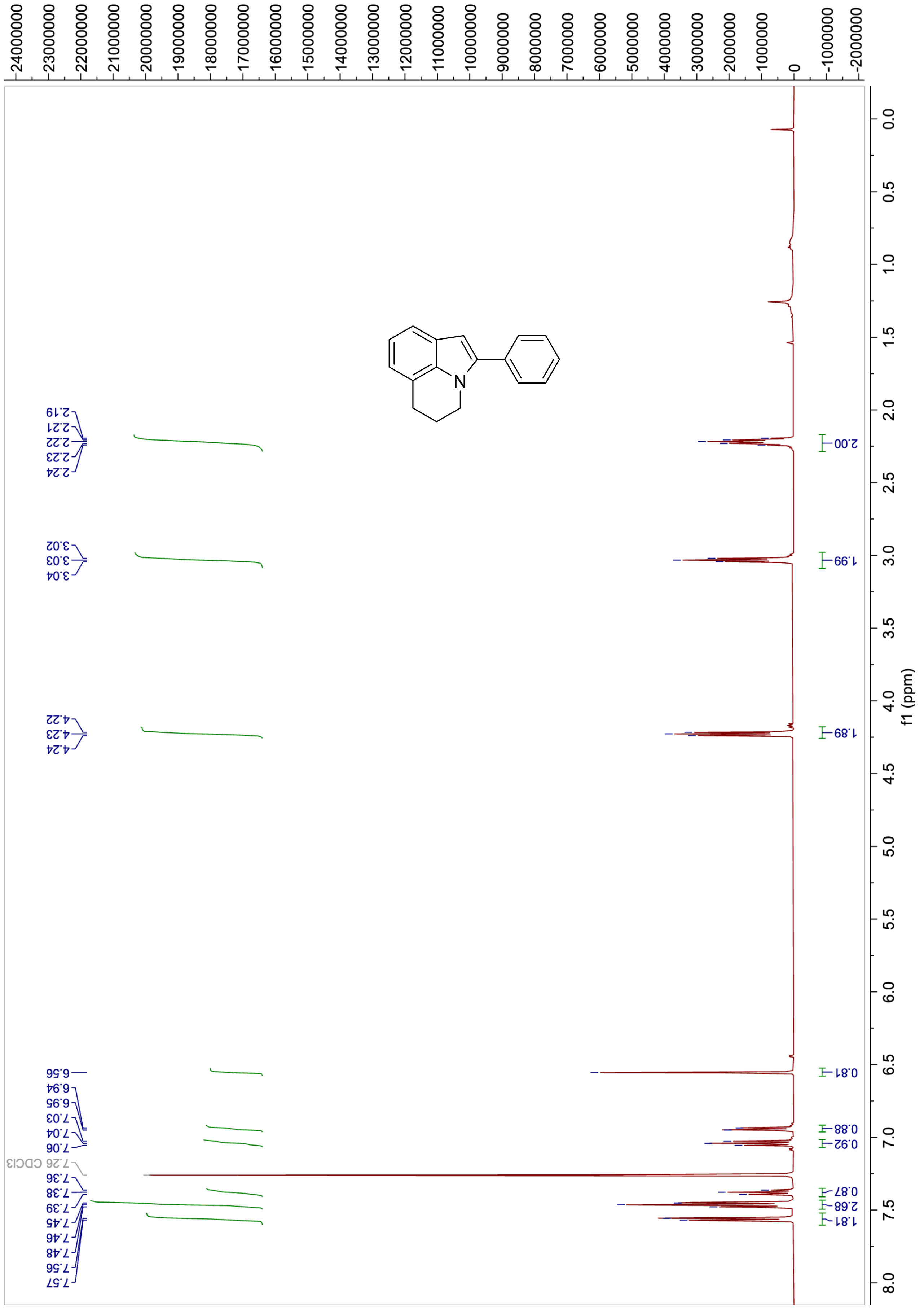

$^{13}C\{^{1}H\}$ NMR 101 MHz **14**

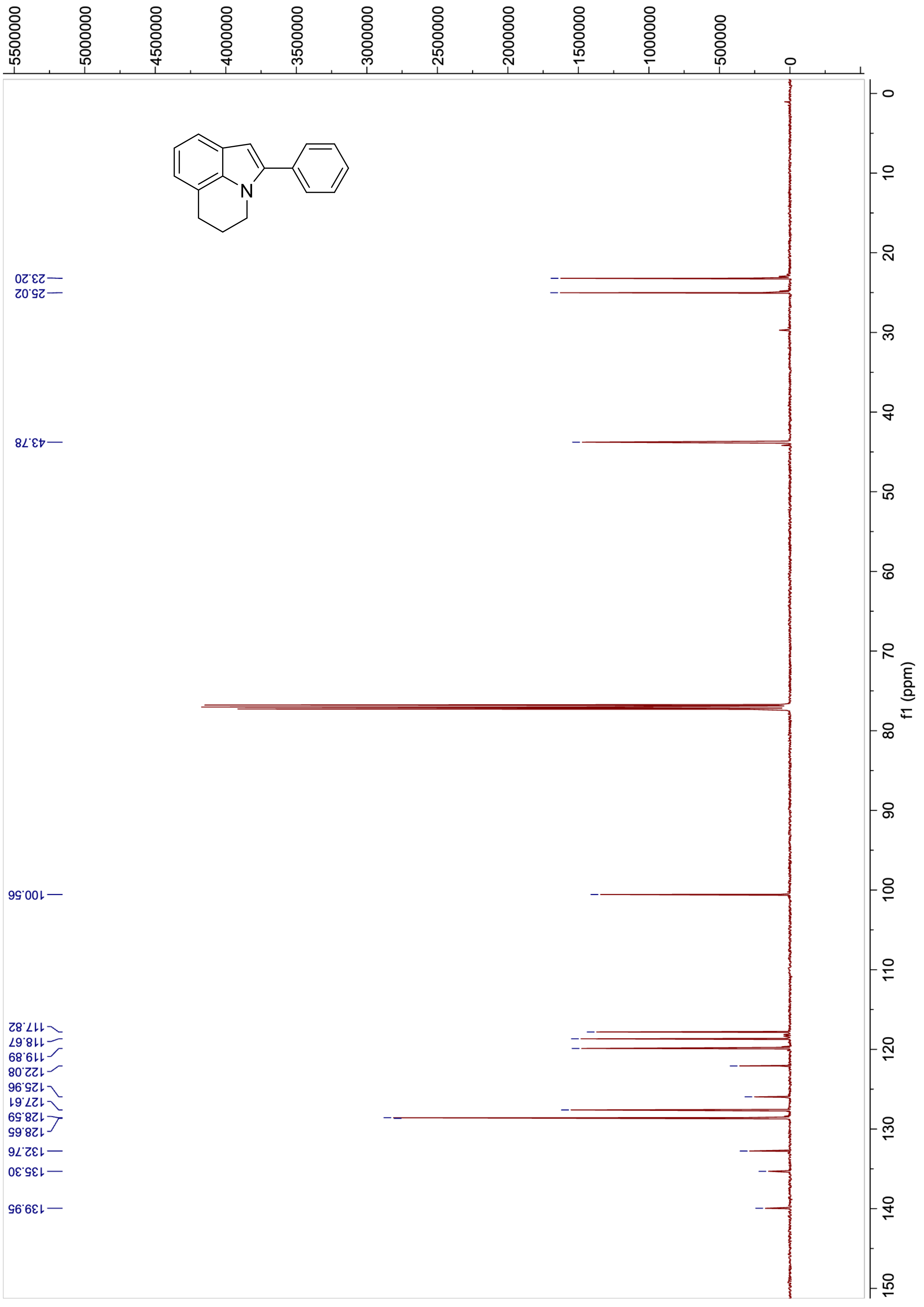

^{1}H NMR 400 MHz **17a**

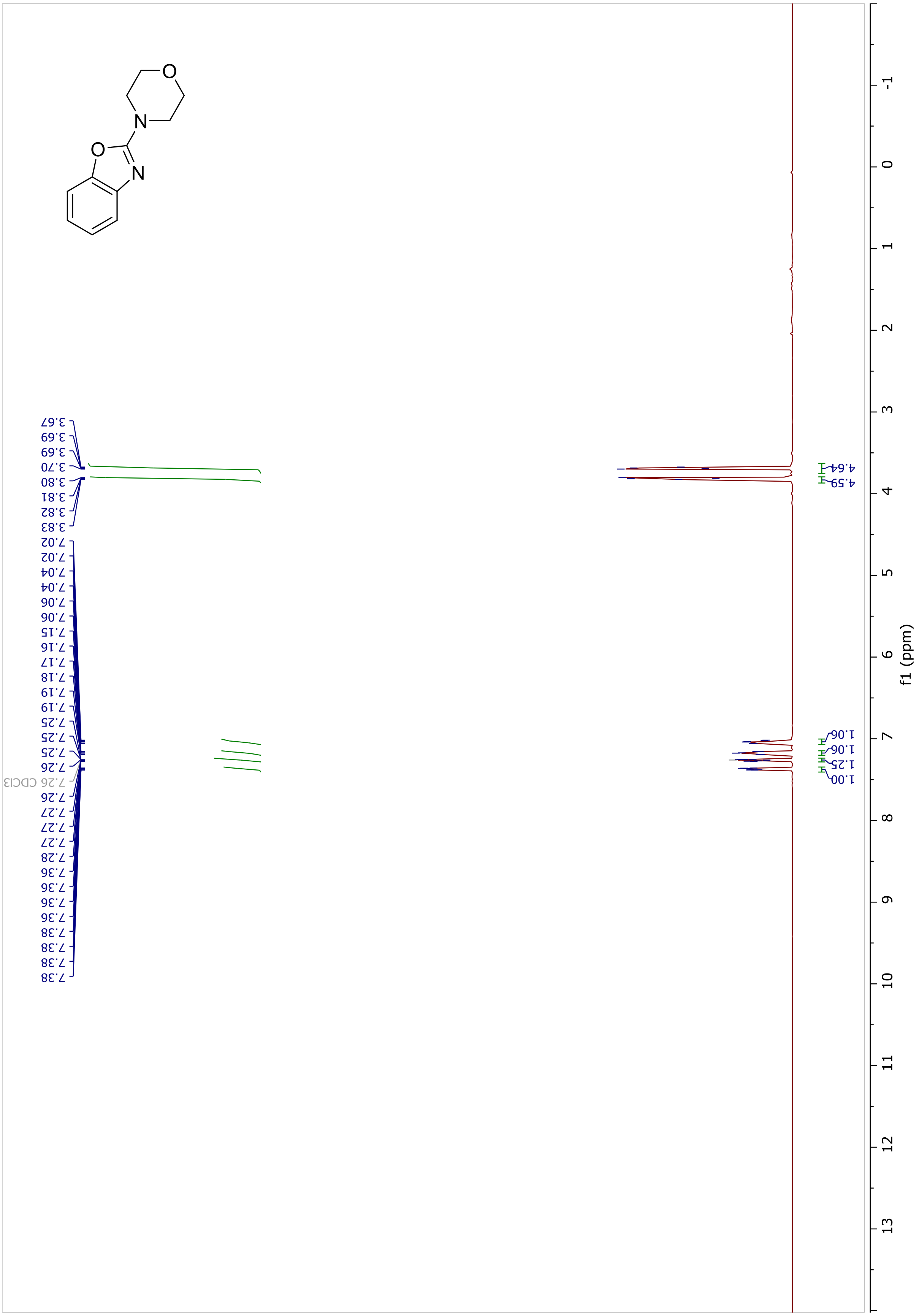

^{1}H NMR 400 MHz **17c**

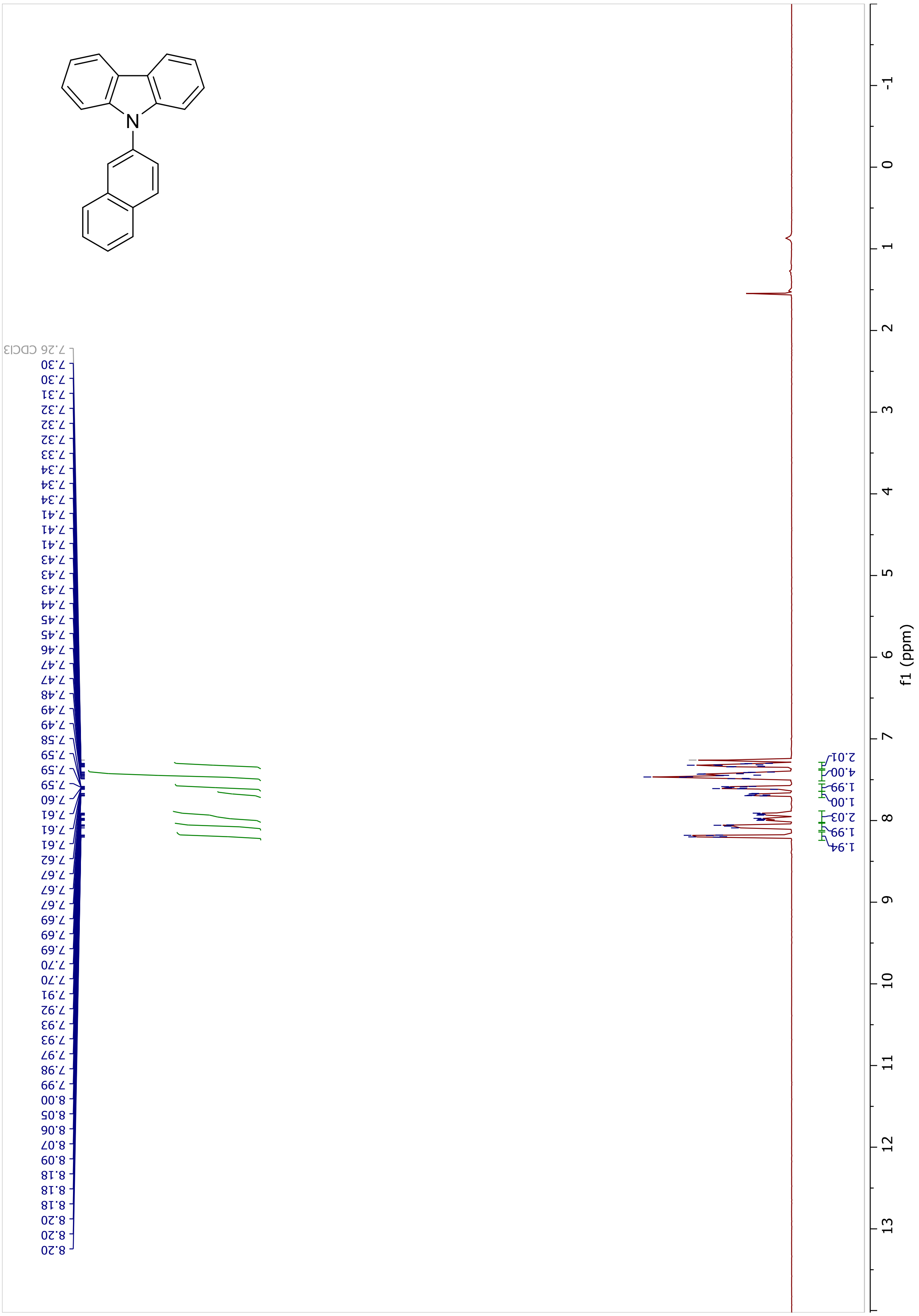

^{1}H NMR 400 MHz **17e**

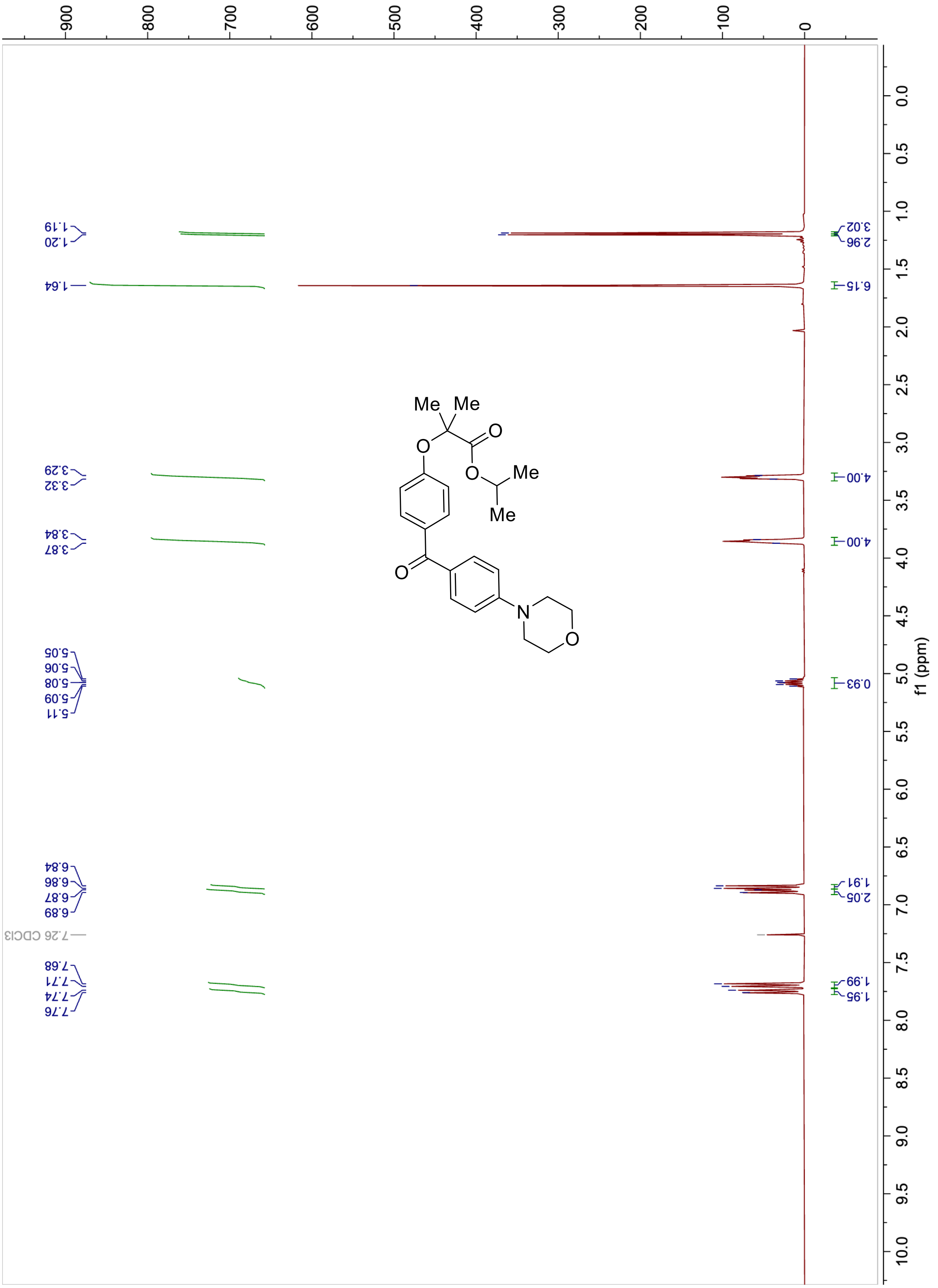

$^{13}C\{^1H\}$ NMR 101 MHz **17e**

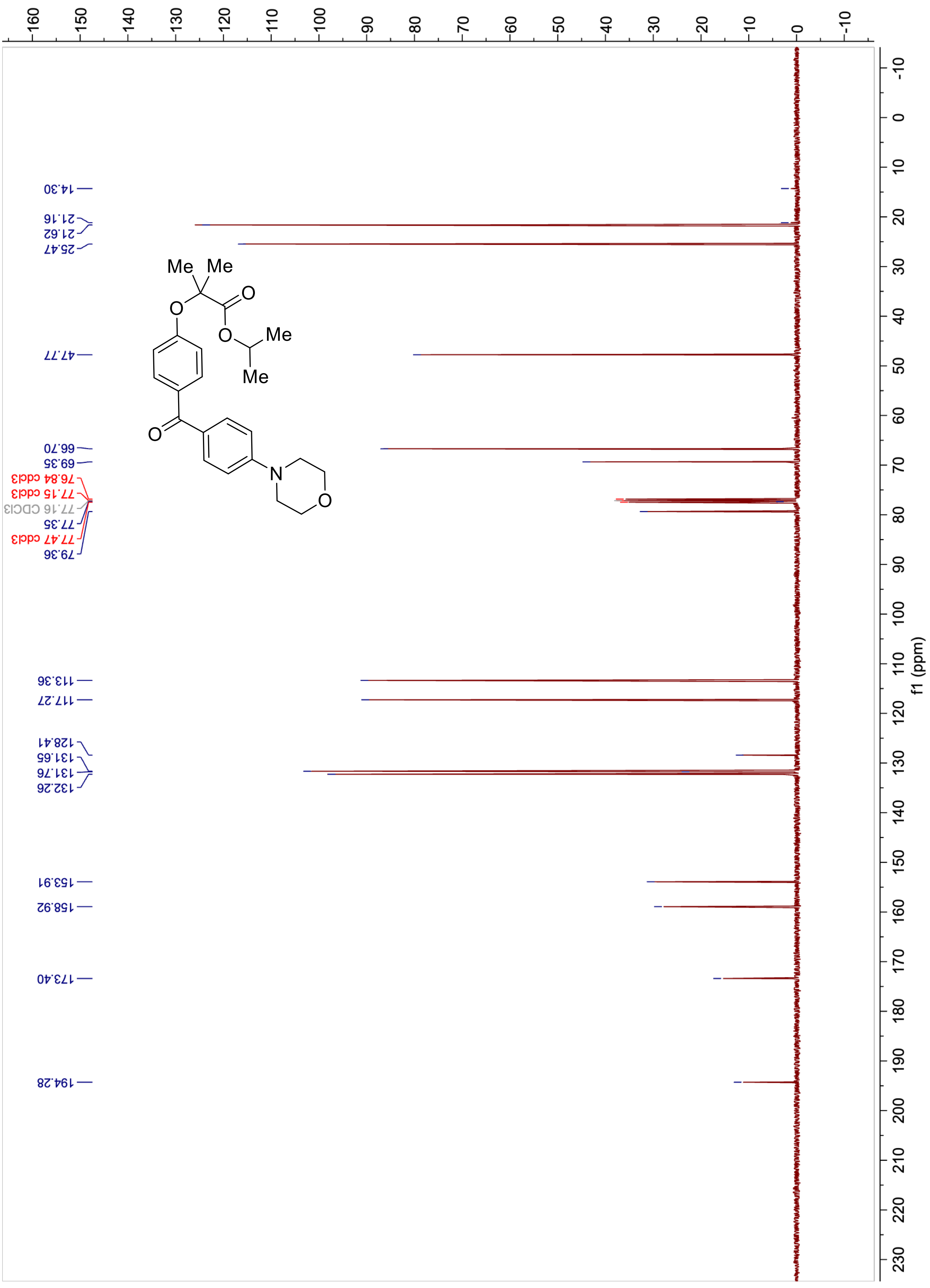

Supplement: Supplementary file 1 [file jo5c00109_si_001.pdf]
